# Leukocyte Motility Models Assessed through Simulation and Multi-objective Optimization-Based Model Selection

**DOI:** 10.1371/journal.pcbi.1005082

**Published:** 2016-09-02

**Authors:** Mark N. Read, Jacqueline Bailey, Jon Timmis, Tatyana Chtanova

**Affiliations:** 1 School of Life and Environmental Sciences, The University of Sydney, Sydney, New South Wales, Australia; 2 The Charles Perkins Centre, The University of Sydney, Sydney, New South Wales, Australia; 3 The Garvan Institute of Medical Research, Darlinghurst, New South Wales, Australia; 4 Department of Electronics, The University of York, York, United Kingdom; 5 St. Vincent’s Clinical School, Faculty of Medicine, University of New South Wales, Darlinghurst, New South Wales, Australia; Cornell University, UNITED STATES

## Abstract

The advent of two-photon microscopy now reveals unprecedented, detailed spatio-temporal data on cellular motility and interactions *in vivo*. Understanding cellular motility patterns is key to gaining insight into the development and possible manipulation of the immune response. Computational simulation has become an established technique for understanding immune processes and evaluating hypotheses in the context of experimental data, and there is clear scope to integrate microscopy-informed motility dynamics. However, determining which motility model best reflects *in vivo* motility is non-trivial: 3D motility is an intricate process requiring several metrics to characterize. This complicates model selection and parameterization, which must be performed against several metrics simultaneously. Here we evaluate Brownian motion, Lévy walk and several correlated random walks (CRWs) against the motility dynamics of neutrophils and lymph node T cells under inflammatory conditions by simultaneously considering cellular translational and turn speeds, and meandering indices. Heterogeneous cells exhibiting a continuum of inherent translational speeds and directionalities comprise both datasets, a feature significantly improving capture of *in vivo* motility when simulated as a CRW. Furthermore, translational and turn speeds are inversely correlated, and the corresponding CRW simulation again improves capture of our *in vivo* data, albeit to a lesser extent. In contrast, Brownian motion poorly reflects our data. Lévy walk is competitive in capturing some aspects of neutrophil motility, but T cell directional persistence only, therein highlighting the importance of evaluating models against several motility metrics simultaneously. This we achieve through novel application of multi-objective optimization, wherein each model is independently implemented and then parameterized to identify optimal trade-offs in performance against each metric. The resultant Pareto fronts of optimal solutions are directly contrasted to identify models best capturing *in vivo* dynamics, a technique that can aid model selection more generally. Our technique robustly determines our cell populations’ motility strategies, and paves the way for simulations that incorporate accurate immune cell motility dynamics.

## Author Summary

Advances in imaging technology allow investigators to monitor the movements and interactions of immune cells in a live animal, processes essential to understanding and manipulating how an immune response is generated. T cells in the brains of *Toxoplasma gondii*-infected mice have previously been described as performing a Lévy walk, an optimal strategy for locating sparsely, randomly distributed targets. Determining which motility model best characterizes a population of cells is problematic; multiple metrics are required to specify as intricate and nuanced a process as 3D motility, and the tools to evaluate model-parameter combinations have been lacking. We have developed a novel framework to perform this model evaluation through simulation, a popular tool for exploring complex immune system phenomena. We find that Lévy walk offers an inferior capture of our data to another class of motility model, the correlated random walk, and this determination was possible because we are able to explicitly evaluate several motility metrics simultaneously. Further, we find evidence that leukocytes differ in their inherent translational and rotational speeds. These findings facilitate more accurate immune system simulations aimed at unravelling the processes underpinning this critical biological function.

## Introduction

Cellular motility and interactions underlie many processes in the immune response, including lymphocyte recirculation through blood and lymphoid organs, their interactions with cells presenting specific antigen, and relocation to the specific tissues where they engage in protective immunity [1]. In the last decade, two-photon microscopy has provided unprecedented insight into how immune cells move and interact *in vivo* [1, 2]. Parallel to this, computational modeling and simulation techniques have been applied to exploring hypotheses of immune system function [3, 4], even simulating the effects of interventions [5, 6].

Agent-based simulations (ABS), wherein individual immune cells are simulated as discrete entities with their own state in a spatially explicit environment, have found widespread application in immunology, with far-ranging applications including: understanding granuloma development [7], Payers patch development [8], the search efficiency of lymphocytes in the lymph node [9, 10], the establishment and subsequent recovery from autoimmune disease [5], and the mechanisms underlying cancer [11]. There is clear scope to combine detailed spatio-temporal two-photon microscopy data with spatially-explicit agent-based simulation to further understanding of how cellular motility integrates with other immune processes to impact health.

An established body of research in ecology has demonstrated, however, the complexities of determining which models of motility best characterize a given dataset. In the Lévy walk model, an agent’s motility is described by a sequence of randomly oriented straight line movements drawn from a power-law, long-tailed distribution [12]. Hence, agent motilities are characterized by many relatively short movements punctuated by rare, very long movements. A diverse range of organisms have been described as exhibiting Lévy walk motility, including bacteria, honey bees, fruit flies, albatrosses, spider monkeys, and sharks [13, 14]. T cells in the brains of *Toxoplasma gondii*-infected mice have also been shown to perform a Lévy walk [15]. Interest in the Lévy walk is in part due to theoretical work demonstrating it an optimal strategy for finding sparsely, randomly distributed targets [16, 17]. However, subsequent work has cast doubt on Lévy walk’s apparent pervasiveness in nature, owing to methodological discrepancies in its identification [18, 19].

The spatial motility of agents in both two- and three-dimensions is an intricate and nuanced phenomenon that cannot be well specified using only one metric. The mean squared displacement over time metric is frequently used to differentiate Lévy walk and Brownian motion characteristics in a dataset, yet models differing in key aspects of motility can produce identical measures [20, 21], e.g., slow moving directionally persistent cells, or fast moving less-directional cells. To accurately simulate the motility dynamics of a biological dataset requires an appropriate motility model assigned appropriate parameter values, and evaluating putative parameter values requires simultaneous consideration of several complementary motility metrics.

Here we evaluate several random walk models’, including Brownian motion, Lévy walk, and several correlated random walks, capacities to capture the motility dynamics of lymph node T cells responding to inflammation and neutrophils responding to sterile laser injury of the ear pinnae. Each model is independently simulated, and those model parameter values that best align simulation and *in vivo* motility dynamics are determined through novel application of a multi-objective optimization (MOO) algorithm: NSGA-II [22]. Parameter estimation is performed through simultaneous consideration of three metrics of cell population motility: the distributions of translational and turn speeds observed across the population, and the distribution of meandering indices. The differences between simulation and *in vivo* distributions generated under each metric form objectives for the MOO algorithm. The resulting Pareto fronts generated under each model, representing parameter values delivering optimal trade-offs in performance against each metric, are contrasted to ascertain which model best captures the biology.

Our random walk models are designed following a detailed analysis of which statistical distributions best fit a cellular population’s translational and turn speed data. Such assessment is complicated by inherent biases in imaging experiments, wherein fast moving and directionally persistent cells rapidly leave the imaging volume. Hence, slower, less directional cells are over-represented in *in vivo* datasets. It is unclear whether cells observed to differ in directional persistence and translational speed are a result of these biases, or whether these observations represent fundamental differences in cellular motilities. Our novel analytical approach fits a given statistical distribution to a population’s pooled translational (or turn) speeds, whilst segregating observations drawn from the distribution into groups that correspond to tracks in the *in vivo* dataset. This segregation reproduces the imaging experiment biases, therein discounting their confounding influence on the analysis. We find that cells comprising our *in vivo* datasets are genuinely heterogeneous, differing in their inherent translational speed and directionality. This finding could reflect intrinsic cellular characteristics, or may arise as features of the environment through which they migrate. In subsequent analysis, we find that translational and turn speeds in both *in vivo* populations are significantly negatively correlated, indicating that cells do not simultaneously perform very fast translational movements and turns. To investigate the significance of these two observations on leukocyte motility we designed four correlated random walk models that differentially include (or exclude) each. We then simulate each to evaluate the integrative impact of these features on overall motility dynamics. We determine that Brownian motion poorly reflects both our datasets. Lévy walk competitively captures directional persistence, but performs poorly on translational and turn speed metrics, underscoring the value of considering several motility metrics simultaneously. Interestingly, for neutrophils Lévy walk provides the most even balance of metric trade-offs of any model examined. Both T cell and neutrophil motility dynamics were better captured by CRWs simulating cells as heterogeneous, rather than homogeneous, populations. Capture of T cell dynamics was further improved by negatively correlating simulated cell translational and turn speeds, however this was not as evident for neutrophil data.

We have provided here evidence, for the first time, that cells within both T cell and neutrophil populations exhibit a continuum of inherent directionalities and translational speeds. Further, we have shown that cells do not simultaneously perform very fast translational and turn movements. We have developed a novel framework to fit statistical distributions to cell translation and turn speeds whilst accounting for experimental bias. Thereafter, the manner in which these two components of motility combine to impact overall spatial exploration is analysed through a novel coupling of 3D agent-based simulation with multi-objective optimization. This latter framework for the first time calibrates and assesses putative motility models through simultaneous consideration of several motility metrics, accounting for trade-offs in performance against each. These frameworks provide the means to robustly analyse and accurately reproduce cellular motility patterns, as they explicitly reflect the constraints of *in vivo* data.

## Results

Our analysis and reproduction of leukocyte motility is performed in two stages. First, we analyse a given dataset’s cellular translation and turn speed dynamics separately. This stage does not attempt to reproduce cellular motility, which is performed later. Instead, it determines the extent to which observed heterogeneity in a cellular population, evidenced through tracks differing substantially in their median translation and turn speeds, is explained by imaging experiment bias, and which statistical distributions best fit this data. In the second stage we construct random walk models based on these distributions, and assess their capacity to reproduce leukocyte motility dynamics through agent-based simulation. *In vivo* data were obtained through two-photon microscopy of mouse lymphoid T cells in explanted lymph nodes in response to challenge and neutrophils in the mouse ear following sterile injury. The motility dynamics of our leukocyte datasets are characterised in S1 and S2 Figs.

### Statistically Heterogeneous Cell Populations with Inversely Correlated Translation and Turn Speeds

**Fig 1:**
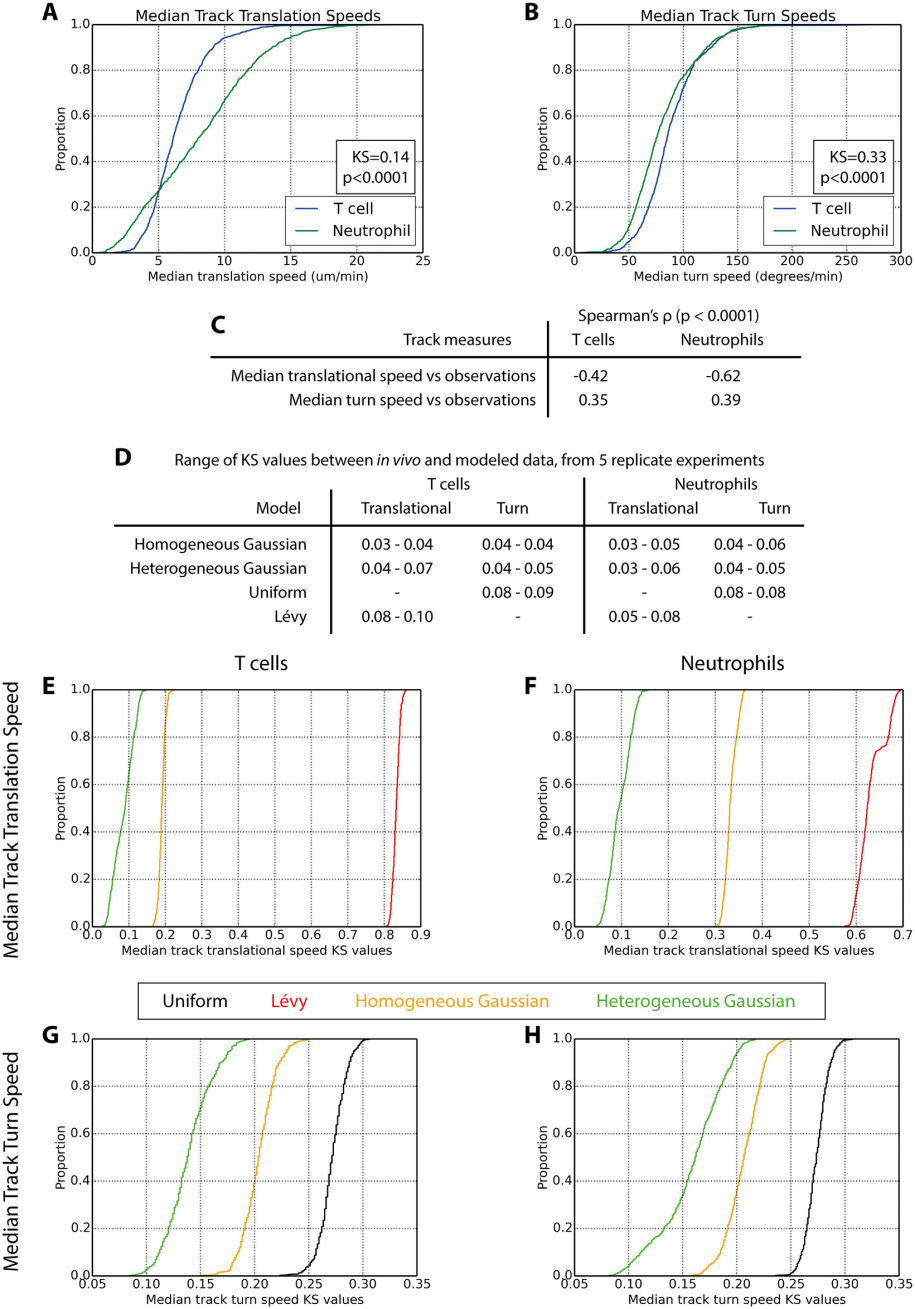
Both T cell and neutrophil populations are statistically heterogeneous: cells comprising each population differ in their inherent speed and directionality. Distributions of varying median track translational (A) and turn (B) speeds were observed in both T cell and neutrophil populations, and plotted as cumulative distribution functions. (C) Spearman’s rank correlation coefficients demonstrate that faster and directionally persistent cells are observed a fewer number of times, as they more rapidly leave the imaging volume. We fitted a variety of statistical distributions to pooled cell translational speed distributions (shown in S1A Fig), and analysed how these individual speeds would need to co-occur in specific tracks to reproduce the median track dynamics observed (A) given imaging experiment biases (C). We investigated ‘homogeneous’ Gaussian, Uniform and Lévy distributions, which assume that the speeds comprising all tracks are drawn from the same distribution, and a ‘heterogeneous’ Gaussian distribution which assumes each track’s speeds are drawn from a bespoke sub-distribution. (D) Each distribution was fitted against *in vivo* translational (and separately, turn) speed distributions pooled from all cells in each dataset 5 times; Kolmogorov-Smirnov (KS) statistic values quantify the quality of fit. Following each fitting exercise 100 modeled datasets were produced, constituting 500 for each statistical distribution. Cumulative distribution plots show the range of KS statistics describing each of these 500 datasets’ alignment with T cell median track translation (E) and turn (G) speeds. Similar plots are shown for neutrophil median track translation (F) and turn (H) speeds.

We hypothesized that our T cell and neutrophil cellular populations were statistically heterogeneous, comprising cells differing in their inherent directionalities and translational speeds. Accordingly, we observed varying median track translational and turn speeds within both cellular populations, Fig 1A and 1B. These distributions could reflect genuinely heterogeneous features between cells, or could represent statistical sampling artifacts arising from finite cellular observation durations within a finite spatial volume. We quantified this experimental bias, Fig 1C, S3 and S1F Figs. Median track translation speed was strongly negatively correlated with the number of times the cell was observed in the imaging volume, and median track turn speed was strongly positively correlated with number of observations. Together these data indicate that fast cells moving in a highly directional fashion quickly left the imaging volume.

We sought to establish whether the perceived heterogeneous cellular characteristics (Fig 1A and 1B) represent a genuinely heterogeneous population, or arise from experimental bias, and which statistical distributions best describe these data. We devised a novel statistical approach to address this question (S4 Fig, Methods and S1 Algorithm), wherein observations are drawn from given statistical distribution and grouped. The groups reflect the structure in which the translational (or turn) speeds observed in a cellular population come from specific tracks. Hence, we could analyse all observations as one pooled dataset or extract the median values across the groups. This structure exactly matches that of the *in vivo* dataset being analysed, with the number of groups matching the number of tracks, and the number of observations within each group matching that of each track. Further, we impose similar correlations between the number of observations in each group and the median observation value, therein reflecting the experimental biases present in the *in vivo* dataset. This is done by establishing the maximum number of observations of any track in the *in vivo* dataset, and initially populating each group with the same number of observations. Thereafter we iterate through each track in the *in vivo* dataset, and select a group from which to discard data such that track and group share the same number of observations, and the correlations between median observation data and number of observations are similar.

This procedure is used to assess how well a given statistical distribution captures cellular translational (or turn) speed data, despite the experimental biases inherent in obtaining it. A successful capture must reproduce both the distribution of all translational (or turn) speeds pooled from all tracks (S1A and S1B Fig), and how these are allocated into tracks yielding the distribution of median track characteristics (Fig 1A and 1B). We assess a variety of statistical distributions, depicted in S5 Fig, including uniform, Lévy and Gaussian; these are termed ‘homogeneous’ as the same parameterized distribution is used to populate all groups with observations. We also assess a ‘heterogeneous’ Gaussian, wherein each group is populated by a bespoke Gaussian sub-distribution; hence, these groups are statistically heterogeneous, each is composed of observations drawn from a (potentially) unique Gaussian.

A given statistical distribution is first fitted to the *in vivo* dataset’s pooled translational speeds (or turn speeds, S1A and S1B Fig respectively), pooling all groups’ observations when performing the fitting. This is done 5 independent times for statistical rigour. We use each fitted solution to generate 100 datasets using the procedure outlined above, giving 500 datasets in total. For each of these, we contrast the groups’ median observation values with the tracks’ median translational (or turn) speed values using the Kolmogorov-Smirnov (KS) statistic. This yields 500 KS values for each statistical distribution we examine. The statistical distribution yielding lowest KS values best reflects the *in vivo* translational (or turn) speed dynamics; these are graphed as cumulative distribution functions in Fig 1E to 1H, explored below. Cellular turn dynamics are analysed using the same procedure, but additionally accounting for the maximum discernible rotational velocities for each track as determined by imaging experiment temporal resolution (Methods, S1 Algorithm and S1 Table).

T cell and neutrophil translational dynamics are better captured with a statistically heterogeneous Gaussian distribution than a homogeneous Gaussian distribution. When fitting distribution parameters against pooled *in vivo* translational speed data both statistical distributions performed well, Fig 1D, S6 and S7 Figs: KS values differentiating modeled and *in vivo* pooled translational speed data were low. However, median track translational speed data were better captured by the heterogeneous Gaussian distribution, Fig 1E and 1F, and S8 Fig. We also evaluated the capacity for Lévy distributions, the foundation of the Lévy walk, to reproduce *in vivo* translational dynamics. The Lévy distribution was competitive with the Gaussian distributions in capturing pooled translational speed data, Fig 1D, but was inferior in its capture of median track translational speed data, Fig 1E and 1F and S8 Fig.

We determined that homogeneous and heterogeneous Gaussian distributions both accurately capture pooled turn speed data, Fig 1D, S9 and S10 Figs, but the heterogeneous Gaussian proved superior in reproducing *in vivo* median track turn speed distributions, Fig 1G and 1H and S8 Fig. We additionally evaluated a uniform distribution’s capture of turn speed dnymaics, which corresponds with Brownian motion and Lévy walk motility models where successive trajectories are uncorrelated. We determined that the uniform distribution provided a competitive reflection of *in vivo* pooled turn speed distributions, but was the worst of the three models in reproducing median track turn speed dynamics.

**Fig 2:**
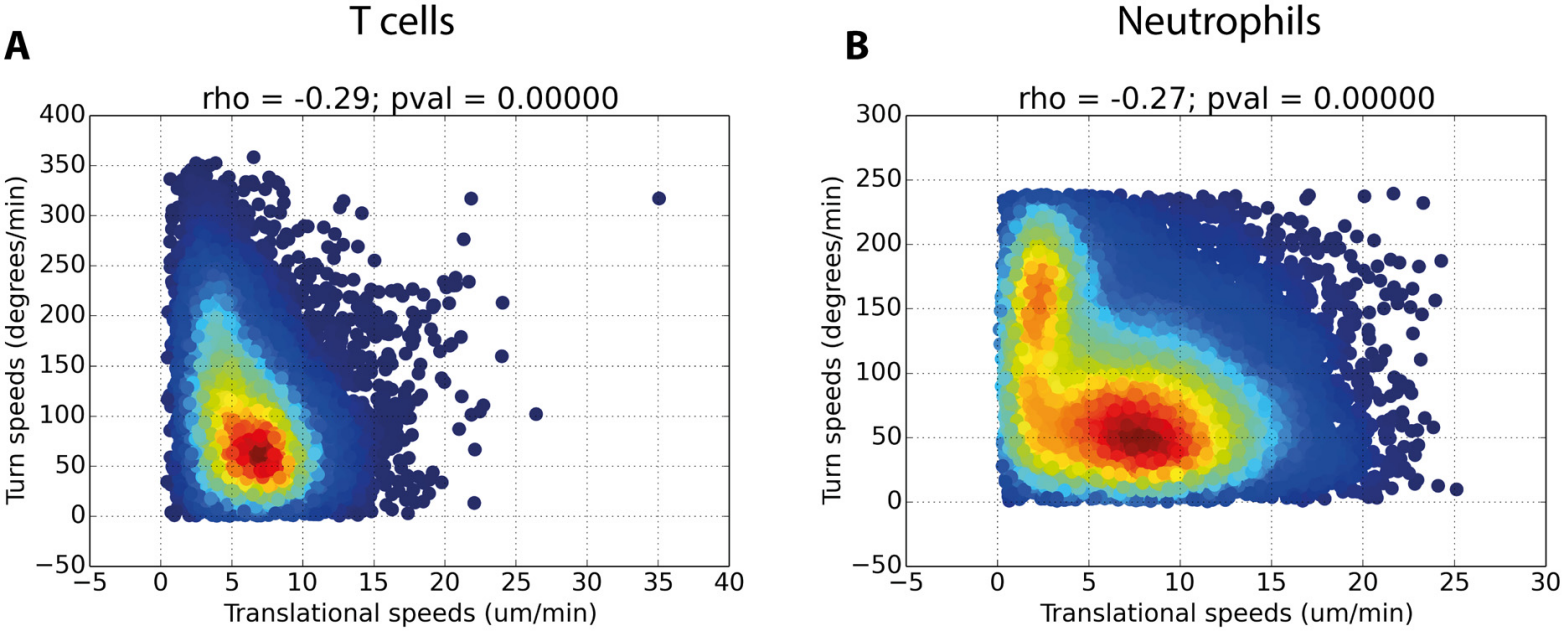
Density scatter plots of cell turn and translational speeds for T cell (A) and neutrophil (B) datasets. Hotter colors indicate a higher density of points. Spearman’s rank correlation coefficients (rho) and p-value are shown. In both cases the p value is smaller than the maximum precision of the test, hence recording 0.

We hypothesized that owing to physical constraints on rates of cytoskeletal remodeling cells are unable to perform both very fast translational movements and turns simultaneously. We confirmed this in both our datasets, Fig 2. The Spearman’s correlation coefficient between cell turn speed and the median of the translational speeds recorded immediately before and after the turn was -0.29 and -0.27 for T cell and neutrophil datasets respectively.

Collectively these data suggest that cells in both our T cell and neutrophil datasets are statistically heterogeneous: the distributions of varying median track translational and turn speeds reflect inherent differences in cellular speed and directionality, rather than sampling artifacts. Further, they suggest a trade-off between fast translational movement and large directional alterations. We next sought to investigate the significance of these observations by designing several correlated random walk models around the statistical distributions explored here, and evaluating their capture of our leukocytes’ spatial exploration through simulation.

### Evaluating Random Walk Models through Calibrated Simulations

Through agent-based simulation we have assessed the ability of six random walk models to reproduce the motility dynamics of our T cell and neutrophil datasets (full details in Methods): Brownian motion, Lévy walk and four correlated random walks (CRW). Table 1 details how these random walk models are designed around the statistical distributions explored in the previous section (see S5 Fig). The HomoCRW and IHomoCRW both represent cellular populations as statistically homogeneous: all cells draw translational speeds from the same homogeneous Gaussian distribution, and similarly for turn speeds. The HeteroCRW and IHeteroCRW models instead define bespoke, potentially unique distributions for each individual cell, rendering them statistically heterogeneous in inherent translational speed and directionality. The IHomoCRW and IHeteroCRW models impose an inverse correlation between translational and turn speeds, whereas HomoCRW and HeteroCRW do not.

**Table 1:** Descriptions of how translational and orientation adjustments are made under each random walk model, and the statistical distributions used therein. See S5 Fig for a graphical illustration of statistical distributions, and the Methods for further details.

Random walk model	Translational speed	Reorientation
Brownian motion	All cells draw speeds from the same zero-mean homogeneous Gaussian distribution.	Each cell selects a new orientation at each time step; all orientations are equally probable.
Lévy walk	All cells draw speeds from the same homogeneous Lévy distribution. A cell will move in its current direction for a period of time, also drawn from a homogeneous Lévy distribution.	If the period of directional persistence has elapsed, a cell selects a new orientation; all orientations are equally probable.
Homogeneous CRW (HomoCRW)	All cells draw speeds from the same homogeneous Gaussian distribution	At each time step all cells select new orientations based on their previous orientations. Each cell draws a turn speed from the same homogeneous Gaussian distribution, which together with the timestep duration determines the adjustment angle from the previous orientation. The plane through which this turn is made is defined by the previous orientation and a second perpendicular vector drawn from a uniform distribution.
Heterogeneous CRW (HeteroCRW)	Identical to HomoCRW, but uses a heterogeneous Gaussian distribution; each cell has a bespoke Gaussian distribution from which speeds are drawn.	Identical to HomoCRW, but uses a heterogeneous Gaussian distribution; each cell has a bespoke Gaussian distribution from which turn speeds are drawn.
Inverse homogeneous CRW (IHomoCRW)	Identical to HomoCRW.	Identical to HomoCRW, but the the turn speed is reduced by a factor proportional to the current translational speed (see Methods). Hence, translational and turn speeds are negatively correlated.
Inverse heterogeneous CRW (IHeterCRW)	Identical to HeteroCRW	Identical to HomoCRW, but the the turn speed is reduced by a factor proportional to the current translational speed (see Methods). Hence, translational and turn speeds are negatively correlated.

Each model was independently implemented in a 3D simulation, and subsequent calibration identified parameter values that align simulation with *in vivo* motility dynamics. Calibration was performed through multi-objective optimization [22], therein simultaneously considering several metrics (*‘objectives’*) of cellular motility. A multi-objective approach is necessary as no single metric can fully specify the complexities of 3D motility. Three objectives are employed, each quantifying a specific difference between the motility profiles of the target *in vivo* dataset and a given model-parameter set simulation dataset respectively. A motility profile constitutes: the distribution of translational speeds observed across all cells at all time points pooled together; similarly for turn speeds; and the distribution of cell meandering indices, defined as a cell’s net displacement divided by its total distance traveled. These distributions are contrasted using the Kolmogorov-Smirnov (KS) statistic, therein forming the three calibration objectives. The meandering index was selected over alternatives such as mean squared displacement (MSD) for it’s ability to capture a distribution of heterogeneous cellular directionalities, which MSD does not; this choice is revisited in the Discussion.

A calibration exercise yields a three-dimensional Pareto front comprising sets of putative model parameter values (*‘solutions’*, S11 Fig). These solutions are Pareto-equivalent: no solutions offer an improvement in any objective without a worsening in another. We evaluate which models best reproduce *in vivo* motility by contrasting their respective Pareto fronts through three complementary analyses (additional details in Methods). Firstly, the proportion of each Pareto front that is non-dominated by each of the others is ascertained (S11 Fig); a solution is dominated if there exists another with at least equal performance on all objectives and superior performance on at least one. Secondly, the best (lowest) 30 Λ values of each front are contrasted; a low Λ value reflects a solution with low mean and variance in its 3 objective KS scores. The best 30 Λ values represent those in the centre of the front, providing good performances on all objectives simultaneously. Lastly, the distribution of KS scores represented across each Pareto front for each objective are directly contrasted. Each model is independently calibrated three times against each *in vivo* dataset, the best solutions of which form a Pareto front for subsequent evaluation. Calibration is performed using NSGA-II [22] for 40 generations, comprising between 20 and 100 candidates per generation, a reflection of the number of model parameters and hence the difficulty of the calibration exercise (see Methods). The performances of the very best solutions found for each motility model, that with the lowest Λ value, against *in vivo* data are shown in S12 to S23 Figs.

### Brownian Motion Poorly Reflects Leukocyte Motility, Lévy Walk Competitively Captures Aspects of Neutrophil Motility

**Fig 3:**
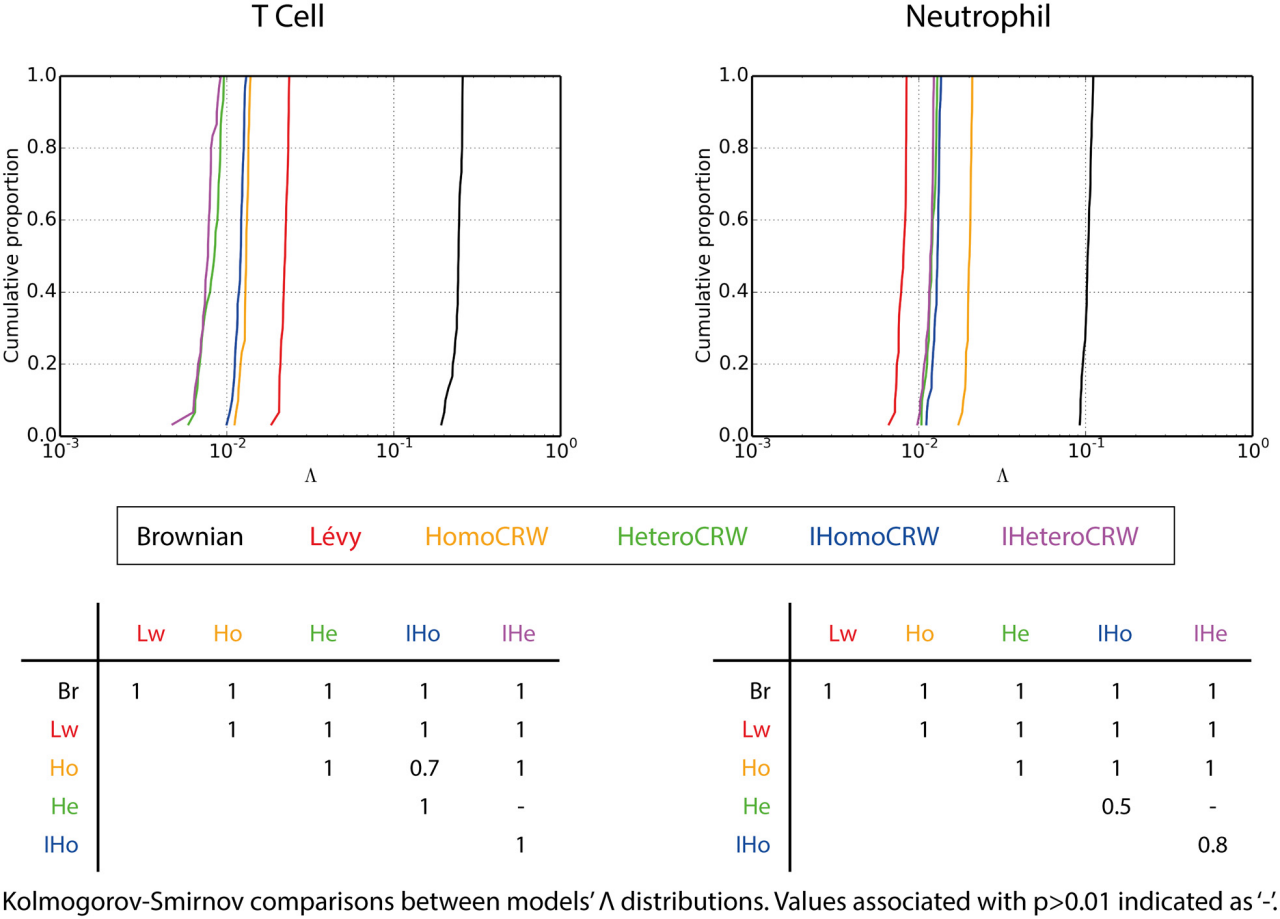
Cumulative distribution plots of models’ 30 best Pareto front members, indicated by low log-transformed Λ values. Tables show Kolmogorov-Smirnov statistic values for comparisons between models; values where p>0.01 indicated as ‘-’.

The Pareto fronts of best calibrated solutions for Lévy walk outperform those of Brownian motion. All Lévy walk solutions are non-dominated by Brownian motion solutions in both datasets, Table 2. For the T cell dataset all Brownian motion solutions are dominated by those of Lévy walk, and for neutrophil data only 7% of solutions are non-dominated. These patterns are reflected in the superior Λ values that Lévy walk solutions’ offer over those of Brownian motion (Fig 3). Brownian motion constitutes a particularly poor reflection of our T cell data, with only 7 tracks in the best Λ value solution remaining after applying a 27*μ*m net displacement filter (applied to reflect *in vivo* data preprocessing to remove anomalous imaging artifacts, see Methods), S12 Fig.

**Table 2:** Comparison matrices showing model superiorities over one another, for both data sets. Superiority is defined as the proportion of one model’s calibration Pareto front (rows) non-dominated by the other’s (columns, see S11 Fig). Each model is represented by a Pareto front representing the pooled Pareto-optimal solutions arising from three independent calibrations. Values in parentheses represent Pareto front sizes.

**T cell dataset**							
		Non-dominated by					
		Brownian	Lévy	HomoCRW	HeteroCRW	IHomoCRW	IHeteroCRW
		(65)	(196)	(248)	(152)	(298)	(104)
Brownian	(65)	-	0	0	0	0	0
Lévy	(196)	100	-	1.0	0.5	0.5	1.0
HomoCRW	(248)	100	100	-	4.4	27.0	2.4
HeteroCRW	(152)	100	100	98.0	-	98.0	9.9
IHomoCRW	(298)	100	100	84.9	6.4	-	5.0
IHeteroCRW	(104)	100	100	99.0	95.2	99.0	-
**Neutrophil dataset**							
		Non-dominated by					
		Brownian	Lévy	HomoCRW	HeteroCRW	IHomoCRW	IHeteroCRW
		(124)	(343)	(458)	(239)	(386)	(139)
Brownian	(124)	-	7.3	0	0	0	0
Lévy	(343)	100	-	72.3	31.5	43.7	45.2
HomoCRW	(458)	100	30.3	-	7.2	8.5	17.9
HeteroCRW	(239)	100	82.0	94.1	-	88.3	67.4
IHomoCRW	(386)	100	56.7	97.7	39.6	-	42.0
IHeteroCRW	(139)	100	75.5	100	50.4	91.4	-

Brownian motion and Lévy walk are inferior to all the CRW models in capturing T cell motility; ≥99% of their solutions are dominated in all cases (Table 2), and they provides the poorest Λ values found in any model (Fig 3).

For the neutrophil dataset, Brownian motion is again suboptimal compared to all CRWs, in terms of both Λ values and non-domination. In contrast to T cell capture, however, Lévy walk solutions are not universally dominated by those of the CRWs, with as much as 72% of the Lévy walk Pareto front being non-dominated by that of HomoCRW. The HeteroCRW and IHeteroCRW models fare better, with >76% of their Pareto fronts non-dominated by that of Lévy walk, versus 32% and 45% *vice versa*. In terms of Λ value performance Lévy walk completely dominates all other models, Fig 3. This is somewhat surprising given the competitive non-domination performances, and likely reflects how performances against each objective, explored below, are balanced in Lévy walk solutions; solutions with equal KS measures on each objective score lower Λ values (Methods).

**Fig 4:**
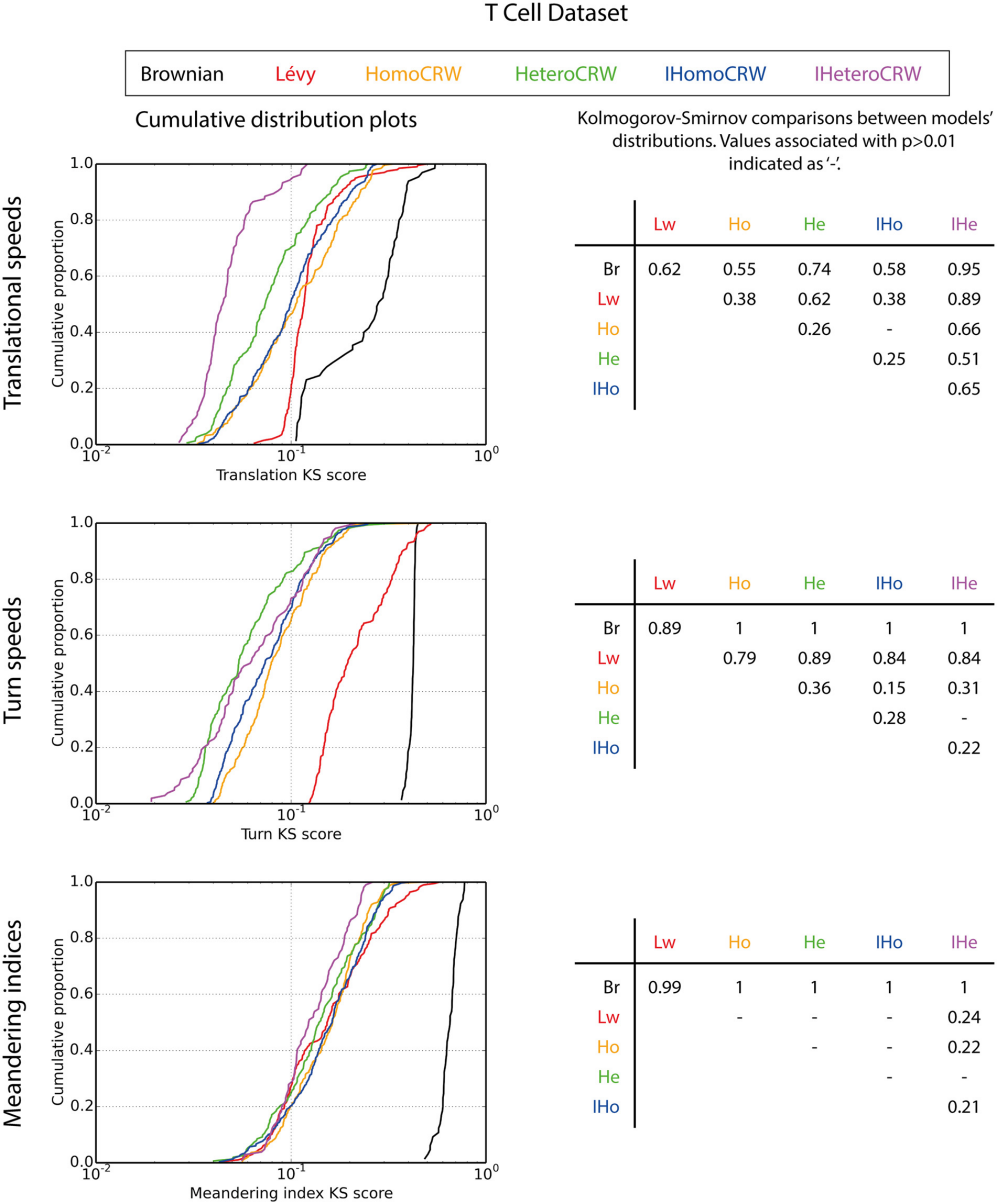
Cumulative distribution plots of log-transformed objective values for each models’ Pareto front when calibrated against T cell data. Tables show Kolmogorov-Smirnov statistic values for comparisons between models; values corresponding to p>0.01 indicated as ‘-’.

**Fig 5:**
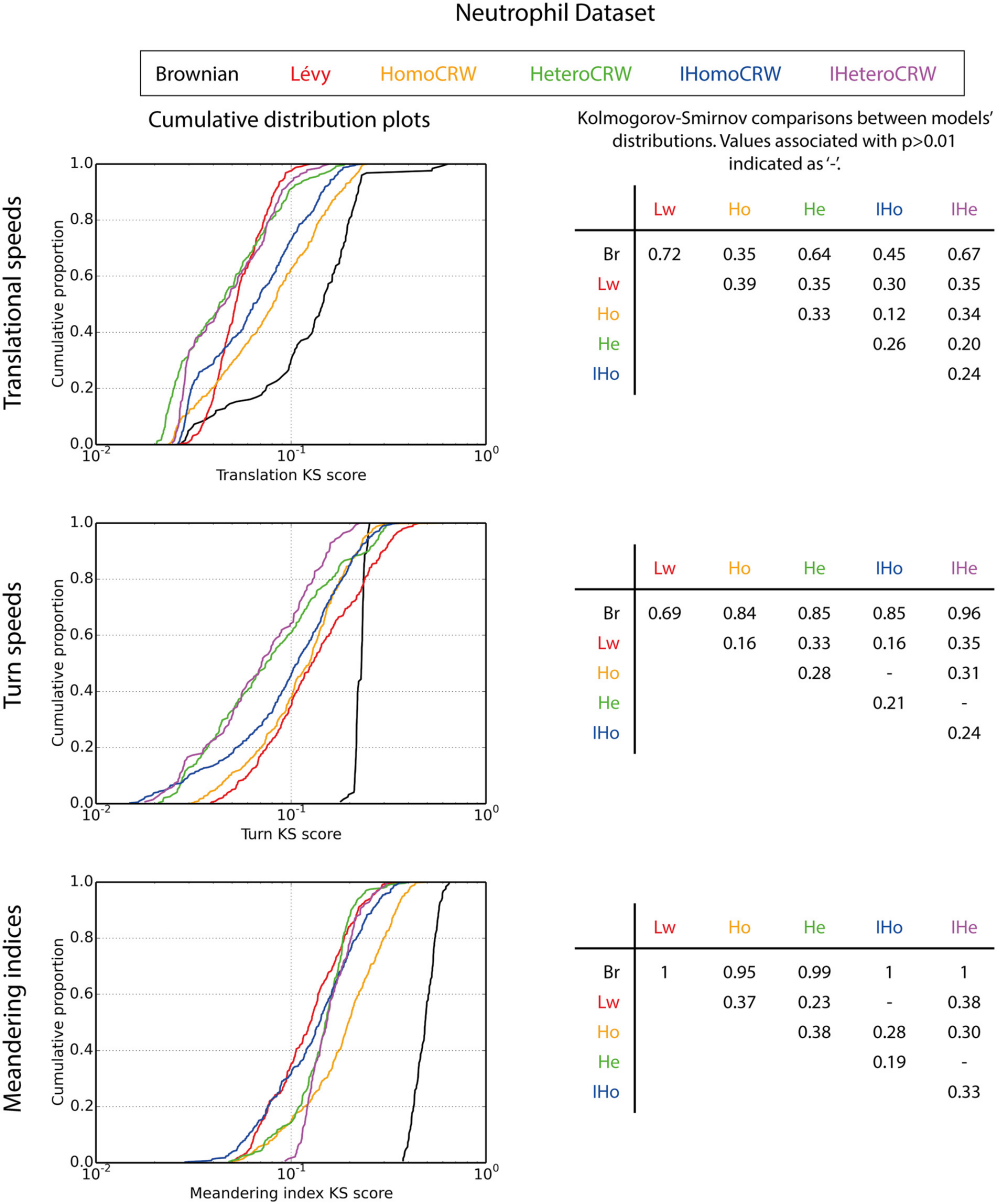
Cumulative distribution plots of log-transformed objective values for each models’ Pareto front when calibrated against neutrophil data. Tables show Kolmogorov-Smirnov statistic values for comparisons between models; values corresponding to p>0.01 indicated as ‘-’.

Lévy walk accurately and competitively captures the directional persistence of *in vivo* data, but Brownian motion does not. Brownian motion delivers a narrow distribution of meandering index KS values, far inferior to other models’ values (Figs 4 and 5 for T cells and neutrophils respectively). Lévy walk mirrors the performance of the best CRW model in capturing neutrophil meandering indices, Fig 5, and is statistically indistinguishable from all CRWs in capturing T cell meandering indices except the IHeteroCRW which offers the best performance, Fig 4.

The meandering index reflects the interplay between cellular translation and orientation adjustments, and it is notable that Lévy walk does not perform particularly well in either of these, despite offering such competitive performance in capturing meandering indices. For turn speed KS values, Lévy walk and Brownian motion represent the poorest fits. Lévy walk offers a poor fit to T cell translational speeds, but exhibits a strangely narrow distribution of KS values for neutrophils; as shown in Fig 5, Lévy walk’s worst translational KS values are better than the worst of the CRWs, however its best are worse than those of CRWs. Brownian motion poorly reflects the *in vivo* translational speed dynamics of both datasets.

The previous section’s exploration of leukocyte heterogeneity found the Lévy distribution to poorly fit to leukocyte median translational speed data, and similarly, the uniform distribution to poorly fit median turn speed data; it is through these distributions that Lévy walk translational and reorientation adjustments are drawn. We analysed the present Lévy walk simulation’s capture of leukocyte heterogeneity, S24 Fig. Overall, Lévy walk performance is better than might be expected given the previous section’s results. It offers competitive or improved capture when contrasted with the HomoCRW and IHomoCRWs, but generally inferior to the HeteroCRW and IHeteroCRW, with the exception of median neutrophil turn speed data.

In summary, Brownian motion is universally poor in capturing both T cell and neutrophil motility. Lévy walk is similarly poor in capturing T cell performance, but for neutrophils the situation is more complex. Though uncompetitive in turn speed capture, moderately so in translational speed capture, and being largely Pareto-dominated by the IHeteroCRW’s Pareto front, Lévy walk offers by far the best performance on Λ values. We theorise that there exists a portion of the neutrophil Lévy walk Pareto front comprising solutions with similar, low mean objective KS values, as this would yield low Λ values despite not dominating in any particular objective alone.

### Heterogeneous- Outperform Homogeneous-CRW Models

We find that CRW models accommodating heterogeneous characteristics between cells better reflect *in vivo* data (Table 2). HeteroCRW Pareto fronts for both datasets are almost entirely non-dominated by the HomoCRW Pareto fronts, versus <7% vice versa. A similar trend is found when comparing the inverse CRW class of models, where IHeteroCRW solutions were largely non-dominated by IHomoCRW models. Here, however, the IHomoCRW was was 42% non-dominated on the neutrophil dataset, substantially higher than the 5% of the T cell dataset.

The superiority of heterogeneous over homogeneous CRW models was also reflected through the best 30 Λ value distributions, Fig 3. We find no overlap in Λ value distributions between HeteroCRW and HomoCRW models on either dataset, with the former providing superior values. On the T cell dataset IHeteroCRW is similarly superior, however neutrophil dataset IHeteroCRW and IHomoCRW Λ distributions overlap, with the former providing marginally superior values. To provide an intuition into the magnitude of the separation between heterogeneous and homogeneous CRW models’ Pareto fronts, S25 and S26 Figs provide three-dimensional plots of Pareto front solutions against each objective KS score. The separation between Pareto fronts is particularly large for HeteroCRW and HomoCRW on the neutrophil dataset, and IHeteroCRW and IHomoCRW on the T cell dataset.

The better fit of heterogeneous over homogeneous models of cellular populations is reflected in performance on each objective (Figs 4 and 5). HeteroCRW yields a distribution of KS values statistically significantly lower than HomoCRW models for all three measures of cellular motility on both datasets, with the exception of T cell meandering indices where no statistically significant difference is observed (Fig 4). We similarly find IHeteroCRW to yield statistically significantly lower values than IHomoCRW on both datasets, with the exception of neutrophil meandering indices where IHomoCRW provides lower values (Fig 5).

Our simulation studies further support our determination that our *in vivo* cellular populations are statistically heterogeneous, and that observed distributions of median track translational and turn speeds (Fig 1A and 1B) are not sampling artifacts. Our simulations impose the same experimental constraints as are present *in vivo*: finite observations of cells within a finite imaging volume. Despite not being used as criteria for model calibration, the heterogeneous CRW models best captured median track translation and turn characteristics, with the exception of neutrophil median track turn speeds (S24 Fig).

### Inverse CRW Formulation Improves Capture of T Cell, But Not Neutrophil, Motility

Analysis of both *in vivo* datasets revealed significant negative correlations between cellular translation and turn speeds (Fig 2). This correlation could impact cellular directionality, and hence meandering indexes, as subsequent fast translational movements would be directionally persistent. As such, we examined whether the inverse CRW formulation, which imposes this quality on simulated cells (see S27 Fig), better reflects the *in vivo* data than the standard formulation.

Inverse CRW models better capture T cell motility dynamics than the standard formulations, but the difference is moderate. 85% of IHomoCRW solutions are non-dominated by HomoCRW models, in contrast to 27% vice versa (Table 2). A larger disparity is found for IHeteroCRW and HeteroCRW models, with values of 95% and 10% respectively. Fig 3 reveals, however, that the magnitude of this dominance is marginal: the Kolmogorov-Smirnov statistic reveals a difference of 0.7 between HomoCRW and and IHomoCRW Λ value distributions, and no statistically significant difference between HeteroCRW and IHeteroCRW models. Given the Pareto-dominance of IHeteroCRW over HeteroCRW models this Λ value finding is surprising, and suggests that the dominance occurs on the periphery of the Pareto fronts; Λ values focus on the centre only. Corresponding analysis of model performances’ on each objective highlights translational speeds as the objective where IHeteroCRW outperforms HeteroCRW; no significant difference is observed on other objectives (Fig 4). IHomoCRW’s capture of T cell turn speeds is significantly better than HomoCRW’s, but we find no other statistically significant differences across objectives. The lack of statistically significant differences between inverse and standard model formulations on meandering index performance is surprising, given that it was specifically this objective that the inverse formulation was hypothesized to offer improvement in. Rather, the inverse formulation facilitates performance improvement on other objectives whilst maintaining a similar meandering index profile. S28 and S29 Figs provide three-dimensional plots of Pareto front solutions against each objective KS scores for standard versus inverse model formulations.

IHomoCRW better captures neutrophil dynamics than HomoCRW, but this finding does not extend to heterogeneous CRW formulations. 98% of IHomoCRW solutions are non-dominated by the HomoCRW model, and 9% vice versa. Conversely, IHeteroCRW and HeteroCRW are largely Pareto-equivalent, where 67% of HeteroCRW solutions are non-dominated in contrast to only 50% of IHeteroCRW models. These findings are supported by Λ value distributions, where IHomoCRW yields substantially better values then HomoCRW, yet no significant difference is found between IHeteroCRW and HeteroCRW models (Fig 3). We find that the IHomoCRW models offers significantly better meandering index and translational speed values than the HomoCRW model, Fig 5. HeteroCRW provides superior translational speeds to IHeteroCRW, but otherwise these two models are statistically indistinguishable.

## Discussion

The advent of two-photon *in vivo* cellular imaging techniques facilitates detailed examination of cellular motility and interaction. The resultant data permits identification of cellular motility strategies, which can be incorporated into broader immune simulations to understand the development and potential manipulation of the immune response. Determining which motility model best fits a biological dataset requires simultaneous consideration of several metrics of motility; three dimensional motility is too intricate a phenomenon to be fully specified in only one metric. Here we have evaluated the capacity of six random walk models, including Brownian motion, Lévy walk and four correlated random walks, to reproduce the motility dynamics of lymph node T cells and neutrophil datasets under inflammatory conditions. Our evaluation is made possible through the development of a novel simulation calibration methodology, where multi-objective optimization identifies parameter values that provide optimal trade-offs for a given model against several metrics of motility.

We found that Lévy walk, an optimal strategy for finding sparsely randomly distributed targets [16, 17], and identified as the motility pattern of CD8+ T cells in *Toxoplasma gondii*-infected mouse brains [15], does not optimally capture our T cell motility dataset. Its performance in capturing neutrophil motility was competitive with other models’, performing well in some motility measures but poorly in others. We attribute our finding to the simultaneous consideration of multiple motility metrics. Lévy walk’s best meandering index performance is competitive with other models’, and as such optimization on that metric alone might highlight Lévy walk as an optimal model (Figs 4 and 5). It is only when performance against this metric balanced with others that Lévy walk’s quality of capture diminishes. It is the micro-level details of leukocyte motility that Lévy walk fails to capture, given their straight-line directional persistence punctuated by uniformly random reorientations of direction. This is supported by our fitting of statistical distributions to cell translational and turn speed data, where Lévy and distributions and uniform distributions (corresponding with uncorrelated cellular trajectories) poorly captured the data. We do not discount the possibility that hybrid strategies, where micro-level correlated random walks are subject to macro-level directional persistence captured by Lévy walks [16], might better reflect *in vivo* data.

We determined our T cell and neutrophil populations to be statistically heterogeneous in their inherent translational speed and directional persistence. We devised a novel approach for fitting statistical distributions to either translational or turn speed data whilst accounting for imaging experiment bias. Our approach ruled out the possibility that these heterogeneous qualities arise as sampling artifacts from observing cells for finite durations within a finite imaging volume; cells that simply happen to be moving fast in a persistent direction as they crossed the imaging volume would give the illusion of being statistically distinct from cells that happened to be moving slowly with little directional persistence at time of observation. We quantified this bias, and found strong negative correlations between median cell track translational speed and observational duration in both datasets. Likewise, we found strong positive correlations between median turn speeds and observational duration. Our statistical distribution fitting approach uses a given distribution to reproduce *in vivo* data, capturing the same number of tracks, the same observations per track, and imposing similar correlations between median track feature and number of observations. A heterogeneous statistical distribution, wherein each track’s data is generated from a bespoke, potentially unique, Gaussian distribution best reflected our *in vivo* data in all cases. Homogeneous distributions, wherein the same parameterized distribution was used to model all tracks’ data could not reproduce the heterogeneity observed *in vivo*, despite accounting for the experimental biases.

We confirmed the significance of this cellular heterogeneity through agent-based simulation, which, rather than separately exploring translation and turn dynamics, integrates them to produce 3D tracks. CRW models representing a continuum of heterogeneous qualities within a cellular population proved superior to treating all cells as statistically equivalent. This finding supports the conclusion that leukocytes differ in their inherent rotational and translational speeds. We discount the alternative conclusion that these more complex models capture nuances (rather than general qualities) of the training data set, as levels of over-fitting were monitored and deemed acceptable (see Methods). The large sizes of our datasets, 751 T cells & 1017 neutrophils (see Methods), further suggest that these heterogeneous qualities do not result from small sample sizes. Banigan *et al.* first described a heterogeneous population of CD8^+^ T cells in uninflamed lymph nodes, characterizing them as two distinct homogeneous sub-populations, 30% of which perform Brownian motion and the remainder a persistent random walk, all of them drawing velocities from the same distribution [21]. In contrast, here we identified an entire continuum of inherent cellular translation and turn characteristics, in both neutrophils in the mouse ear pinnae, and lymph node T cells, both under inflammatory conditions.

Analysis of both our T cell and neutrophil datasets revealed strong inverse correlations between cell translational and turn speeds: cells do not simultaneously perform fast translational movements and large reorientations. This has been shown previously for neutrophils [23], but we are unaware of any such finding in T cells. We again used simulation to evaluate the impact of this characteristic on overall motility, devising CRWs that impose this negative correlation (‘inverse’ CRW) and contrasting their capture of *in vivo* dynamics with those that do not. We found inverse CRWs to better capture T cell data than standard formulations, in particular improving capture of translational speeds when coupled heterogeneous qualities. In neutrophil data, an inverse homogeneous CRW substantially improves upon standard homogeneous CRW performance, yet inverse and standard heterogeneous CRW models are indistinguishable. This finding could originate from constraints on the cytoskeleton remodeling processes [24]. Alternatively, cellular dynamics can be explained through the configuration of obstacles in the environment [25]; our findings might represent features of the environment rather than the cell, where cells must slow in order to move around an obstacle. We conclude that the inverse heterogeneous CRW models best capture leukocyte motility: their corresponding Pareto fronts are non-dominated by any other model (Table 2), with one exception where IHeteroCRW and HeteroCRW were indistinguishable.

Previous lymphocyte modeling efforts have incorporated explicit cellular arrest phases between periods of fixed speed, straight-line motility [15, 26]. Our *in vivo* datasets do not record cells as being stationary, or moving in straight lines (S1A and S1B Fig). As such, we have explored CRW models that explicitly capture distributions of translational and turn speeds. Other work has focused on modeling lymphocytes as point-processes confined to the lymph node reticular network [27], explicitly modeling cellular morphology [25, 28], and conceptualizing cell trajectories as features of environmental obstacles [25]. The possibility of calibrating the configuration of an environment by proxy of the resultant cellular motility is intriguing. Our multi-objective optimization framework is independent of the motility paradigm and could be more broadly applied in these contexts.

We opted to employ three objectives in our multi-objective approach, based on the pooled translational speeds of all cells across all time points into a single distribution, similarly for turn speeds, and track meandering indices. We consider this the minimum required to accurately specify motility, capturing how cells move translationally through space, how subsequent trajectories are correlated, and how these two aspects integrate to define overall spatial coverage. Multi-objective optimisation can accommodate more objectives, and hence additional motility metrics could be incorporated (or substituted). In particular, we believe there is merit in studying how recent, more sophisticated motility metrics might be incorporated into our framework [20, 21]. It is practical, rather than technical, considerations that limit the number of objectives one can use: in our experience the number of Pareto front members tends to increase with each additional objective, and more objectives constitute a more complex problem which can require greater computational effort to solve to a similar extent (e.g., as measured through objective KS values). Convention in multi-objective optimisation dictates that one choose objectives which are not correlated with one another; to do so increases the complexity of the optimisation problem whilst providing little benefit in capturing better quality solutions. Candidates for additional objectives might include the median track translational or turn speed distributions, however we note that for our favoured motility model, the inverse heterogeneous CRW (IHeteroCRW), these characteristics are well captured despite not being explicit criteria in model calibration (S17B, S17C, S23B and S23C Figs).

We consider it essential to include an objective capturing how translational and turn characteristics integrate to dictate spatial coverage. In this regard we employ the meandering index, but possible alternatives include mean squared displacement (MSD) or spatial volume explored. Each of these introduces some bias, and hence the decision is somewhat arbitrary. For instance, meandering indices tend towards 1 for short tracks; S2A and S2B Fig quantify this for our *in vivo* datasets. We note, however, that our simulation approach imposes the same experimental constraints as exist *in vivo*, and all our data are processed through the exact same analytical pipeline (see Methods). As such the same biases arise in all our data, providing a fair comparison between *in vivo* and simulation experiments. It is notable that similar correlations and scatter plots occur between track duration and meandering index for our simulation and *in vivo* datasets (T cells: S2A, S12H to S17H Figs; neutrophils: S1C Fig) MSD has been used extensively to discriminate between motility models, however, in addition to the known issues with this metric [20, 21], our characterisation of statistically heterogeneous populations prompted our choice of the meandering index, which neatly captures the distribution of cellular directional persistencies and which the MSD does not. We have performed a pilot study substituting MSD in place of the meandering index, calibrating the IHeteroCRW model against neutrophil data (details in Methods). Capture of pooled translational and turn speed data formed the remaining two objectives. S30 Fig contrasts IHeteroCRW’s capture of neutrophil motility under each calibration scenario. As to be expected, the meandering index and MSD metrics were best aligned when used directly as a calibration objective. Pooled translational speed data was best captured using the meandering index, and turn data capture was statistically indistinguishable. Interestingly, median track translational speeds were better captured using the meandering index, and median track turn speeds through MSD (neither were used in driving calibration). The best single solution arising from the MSD calibration is shown in S31 Fig, and can be contrasted with that of meandering index calibration, S23 Fig. Again, the results are remarkably similar, with the exception that using the meandering index better captures median track translational speeds and correctly captures the *in vivo* MSD, whilst calibrating with MSD poorly captures *in vivo* meandering indices. The similarities in these data support our belief that both meandering index and MSD capture similar aspects of motility when coupled with metrics of pooled translational and turn speed data, as in the current context. Banigan *et al.* have proposed metrics capturing displacement probability densities, and displacement autocorrelations for given time intervals [21]. We consider these metrics more statistically robust than either the meandering index or MSD, and have calculated displacement autocorrelation measures for our leukocyte and modeled datasets (S32 and S33 Figs). However, given our focus on cellular heterogeneity, captured in both the data spread at each time interval and how individual cells perform across intervals, it is not clear how to integrate such high dimensional data into an objective to be used in the present calibration framework. This we highlight as meritorious further work. We note that the IHeteroCRW model generally deemed superior by our present methodology also provides a close qualitative alignment with *in vivo* displacement autocorrelation data.

Our novel method for contrasting putative models, and therein parameterizing them, has a valuable role to play in the development of biological simulations. The construction of simulations which demonstrably capture biological systems has received recent attention [29]. This resulted in a process through which assumptions underpinning the abstraction of key biological components and processes into a conceptual model and thereafter a software implementation are explicitly captured [30]. A complementary technique, borrowing from safety critical systems engineering, decomposes a claim such as “This simulation is an adequate representation of the biology” into sub-claims against which evidence is cited [31]. Additionally, statistical analyses quantifying the impact of biological uncertainty on simulation results by highlighting critical parameters and pathways have been developed [32, 33]. Together these techniques support the development and interpretation of biologically meaningful simulations. The novel multi-objective optimization approach developed here is complementary in helping select between competing abstractions of the biology by providing numerical evidence of improved capture. Whilst there exist established model selection techniques such as the Akaike Information Criterion and Schwarz criterion [34], it is unclear how to apply them over multiple metrics of biological capture, as in the present case. A strength of both the Akaike Information Criterion and the Schwarz criterion is their consideration of model parameter number when determining the most appropriate model. This feature is currently lacking from our multi-objective approach, and we see value in further work investigating how to reconcile these approaches. In the context of our present simulation work, the model with the most parameters (inverse heterogeneous CRW) yielded either the outright or joint best capture of the biology. We note, however, that this model’s parameters and the features they represent are not arbitrary, but are instead biological driven: they were found to be present in both our *in vivo* datasets.

Simulation parameterization presents another challenge in biological simulation. The required biological data do not always exist as the corresponding experiments either have not or cannot be performed, and simulation’s abstractive nature complicates their adoption. Existing parameterization approaches include exhaustive search of all possible parameter value combinations [35, 36], maximum-likelihood estimation [15], various forms of regression [37], and genetic algorithms [38]. These techniques do not always scale to simulations with many parameters, and none accommodate the simultaneous consideration of several metrics of simulation’s capture of the biology as our present MOO-based approach does.

We have developed and demonstrated a technology that more robustly determines which motility strategies best characterize a given biological dataset. Furthermore, it can implicitly embed these motility dynamics in a simulation, therein enabling more accurate simulations of immune response development. The intricate and nuanced motility patterns that our method reproduces are important, as it is at this scale that two nearby cells either contact or not, and these interactions can have a profound downstream influence on the immune response. Our approach can be used to characterise and quantify, in detail, how various factors impact and manipulate cellular motility, such as was done through inhibition of LFA-1 affinity and avidity regulation in T cells [39].

## Materials and Methods

### Ethics Statement

All procedures involving mice were reviewed and approved by the Garvan/St Vincents Animal Ethics Committee (AEC). The AEC fulfills all the requirements of the National Health and Medical Research Council (NHMRC) and the NSW State Government of Australia.

### *In Vivo* Imaging of T Cells and Neutrophils

Neutrophil data was obtained using *in vivo* two-photon microscopy of ear pinnae in anesthetized C57/BL6 mice. Neutrophils were recruited in response to sterile needle injury and neutrophil migration was recorded and analyzed following the induction of a small sterile laser injury as was described previously [40]. Neutrophils were visualized with the aid of Lysozyme M fluorescent reporter.

The analysis of lymphocyte motility fluorescent lymphoid cells were adoptively transferred and cell migration was visualized 24 hours later in explanted cervical lymph nodes perfused with warmed and oxygenated medium. Inflammation was induced using either *S. aureus* bioparticles or ovalbumin in Sigma adjuvant.

Two-photon imaging was performed using an upright Zeiss 7MP two-photon microscope (Carl Zeiss) with a W Plan-Apochromat 20′/1.0 DIC (UV) Vis-IR water immersion objective. High repetition rate femtosecond pulsed excitation was provided by a Chameleon Vision II Ti:Sa laser (Coherent Scientific) with 690-1064nm tuning range. We acquired 3*μ*m z-steps at 512×512 pixels and resolution 0.83*μ*m/pixel at a frame rate of 10 fps and dwell time of 1.27*μ*s/pixel using bidirectional scanning. Neutrophil dataset z-depths were 180*μ*m, and T cell dataset z-depths ranged from 150 to 220*μ*m. Both datasets were cropped using Imaris software to correct for tissue drift as needed.

Raw image files were processed using Imaris (Bitplane) software. A Gaussian filter was applied to reduce background noise. Tracking was performed using Imaris spot detection function to locate the centroid of cells and x,y and z coordinates of each spot were exported together with track ID and time interval information.

The T cell calibration data is pooled from 9 individual imaging datasets, comprising a total of 751 cells tracked for a total of 20424 spots, yielding a mean of 27 spots per track. The neutrophil dataset comprises data pooled from 6 individual imaging datasets, totaling 1017 cells encompassing 24619 spots, a mean of 24 spots per track. The T cell experiments were conducted for around 30 min with time-series data recorded every 35 seconds, and for 45 min with time samples every 45 seconds for neutrophil data; exact figures are given in S1 Table.

### Fitting Statistical Distributions to Cell Translational and Turn Speeds

Several statistical distributions, graphically depicted in S5 Fig, were independently fitted to a given dataset: either cellular translational or turn speed data. This was performed for both our T cell and neutrophil datasets independently of one another. A graphical overview of our method is given in S4 Fig.

We obtain a Lévy distributed random variable as follows, adapted from [41]:

$$L\left(\alpha ,\beta \right)=\beta \left|\frac{\sin(\alpha X)}{\cos{\left(X\right)}^{1/\alpha }}{\left(\frac{\cos((1-\alpha )X)}{Y}\right)}^{(1-\alpha )/\alpha }\right|$$ (1)

Where random variable *X* has uniform density on the interval [−*π*/2, *π*/2]; *Y* has unit exponential density, generated as *Y* = − ln*Z* where *Z* is uniformly distributed over [0, 1]; and *β* is a scaling factor. *L* is symmetrical around 0 and hence we take the absolute value, represented as |*x*|.

A ‘homogeneous Gaussian’ distributed variable *G*(*μ*, *σ*^2^) has mean *μ* and standard deviation *σ*^2^. It is homogeneous in that the same parameterized Gaussian is used to represent all cells’ translational (or turn) values. In contrast, a ‘heterogeneous Gaussian’ distribution comprises a bespoke Gaussian $G_{i}\left(\mu_{i},\sigma_{i}^{2}\right)$ for each cell *i* in the dataset. The mean *μ*_*i*_ and standard deviation $\sigma_{i}^{2}$ of *G*_*i*_ are themselves drawn from Gaussian distributions; this is done once at *G*_*i*_’s creation, and the values are maintained throughout *G*_*i*_’s use thereafter. Hence, a heterogeneous Gaussian is formulated as $G_{i}\left(\mu_{i}=G\left(\mu_{M},\sigma_{M}^{2}\right),\sigma_{i}^{2}=G\left(\mu_{S},\sigma_{S}^{2}\right)\right)$, and has parameters *μ*_*M*_, $\sigma_{M}^{2}$, *μ*_*S*_ and $\sigma_{S}^{2}$.

*U*(*λ*) represents a uniformly distributed random variable over the range (0, *λ*].

The parameters describing each statistical distribution are shown in Table 3:

**Table 3:** The parameters characterising each statistical distribution used in this manuscript.

Statistical distribution	Parameters
Homogeneous Gaussian	*μ*, *σ*^2^
Heterogeneous Gaussian	*μ*_*M*_, $\sigma_{M}^{2}$, *μ*_*S*_, $\sigma_{S}^{2}$
Lévy	*α*, *β*
Uniform	*λ*

To evaluate the capacity for a given statistical distribution, *D*, to reproduce an *in vivo* dataset’s translational data we create an artificial dataset of similar structure. Values are drawn from *D*, and allocated into groups. There is one group for each track in the *in vivo* dataset, and initially each group contains as many observations drawn from *D* as the maximum number of observations found in any *in vivo* track. S1 Algorithm, in the supplementary data, discards observations from each group such that the number of observations in each group exactly matches the number of observations in a specific *in vivo* track. The observations to be discarded from each group are chosen such that the correlation between the number of observations in groups and the median observation values of those groups align with the correlations found for *in vivo* tracks. In this manner, the artificial dataset generated by *D* reflects the experimental bias inherent in the *in vivo* data. The pooled observational data, and the median observation values amongst the groups are then extracted, and contrasted with *in vivo* translation or turn data being analysed as follows.

Let *T* represent the target data, be it either translational or turn speed data from one of our datasets, to which a given statistical distribution is to be fitted. First *D* is fitted against the pooled data *T*, that is, all the translation/turn observations pooled into one distribution. Fitting is performed using the python *scipy.optimize.minimize* method, using the ‘Powell’ solver, on the basis of minimizing the Kolmogorov-Smirnov (KS) statistic between pooled *T* data and pooled data generated using *D* in S1 Algorithm. This is performed 5 independent times, the results of which are shown in S6, S7, S9 and S10 Figs. Upon the conclusion of each fitting exercise, 100 further datasets are generated using the fitted *D*. We quantify how well each dataset captures the median track data in *T* using the KS statistic, yielding a total of 500 KS values for each *D*. Contrasting these 500 KS values reveals which statistical distribution best captures *T*, with low values indicating a better capture. The best alignment for each model on each *in vivo* dataset is shown in S8 Fig.

We highlight that this procedure does not attempt to reproduce cellular motility in space, which is an emergent product of how translational and turn movements are integrated. Rather, it determines which distributions best capture translational and turn data independently of one another, and assess whether cells are heterogeneous in these characteristics. We design several random walk models based the distributions investigated here, and assess their capture of cellular motility in space through 3D agent-based simulation, as detailed in the Sections that follow.

### Leukocyte Random Walk Models

The six random walk models explored in this paper are detailed below. The models are constructed around the statistical distributions described above, and illustrated in S5 Fig. Table 1 summarizes which statistical distributions are employed in each random walk model, and how. The random walk models are simulated over time, and as we adopt the notion *D*_*t*_ to indicate a value drawn from randomly distributed variable *D* at time *t*.

The random walk models are implemented in a discretized time, three dimensional continuous space agent-based simulation wherein cells are implemented spheres that cannot overlap. Only cells residing within a 412×412×100*μ*m volume are tracked, replicating *in vivo* experimental conditions. T cell simulation state was updated and recorded for downstream analysis every 30s, and simulation were executed for 30min of simulated time. The corresponding neutrophil figures are 45s and 50min. These values were selected to broadly mirror *in vivo* experiments, as described in S1 Table. Note that Lévy walk simulation states were updated every 3s instead, owing to the variable cell run-durations of this model, however simulation state was still recorded every 30s and 45s as with other models.

#### Brownian motion

Here a cell’s speed along each axis is drawn from a zero-mean Gaussian distribution. This is implemented as a cell selecting a new orientation in 3D from a uniform spherical random distribution, i.e., all orientations in 3D are equally probable. The cell then moves forward in that direction with speed |*G*_*t*_(0, *σ*^2^)|, where |*x*| represents absolute value (cells do not move backwards).

#### Lévy walk

Here cellular motility dynamics are built around Lévy distributions. In the present model cells move in a constant direction at speed *s* for duration *d*, which are selected from Lévy distributions *L*_*t*_(*α*_*T*_, *β*_*T*_) and *L*_*t*_(*α*_*D*_, *β*_*D*_) respectively. When the duration has elapsed, cells select a new orientation from a uniform distribution; all orientations are equally probable.

#### Homogeneous correlated random walk (HomoCRW)

Consecutive movements in this model are correlated; a movement in a particular direction will likely be followed by another in a similar direction. At each time iteration *t*, the cell changes its orientation (its heading) at rotational speed *ϕ*_*t*_. *ϕ*_*t*_ and the timestep duration together define the angle of orientation change, which is made through a plane drawn from a uniform distribution but that the previous orientation lies along; the planes of successive orientation change are uncorrelated. Hereafter the cell moves forward with speed *ζ*_*t*_. *ϕ*_*t*_ and *ζ*_*t*_ are selected from homogeneous Gaussian distributions, common to all cells:

$$\phi_{t}=G_{t}\left(\mu_{P},\sigma_{P}^{2}\right)$$ (2)

$$\zeta_{t}=\left|G_{t}\left(\mu_{T},\sigma_{T}^{2}\right)\right|$$ (3)

#### Heterogeneous correlated random walk (HeteroCRW)

This random walk differs from HomoCRW in that the distributions underlying *ϕ*_*t*_ and *ζ*_*t*_ are unique to each individual cell, thereby permitting a heterogeneously motile population of cells. For a given cell, these distributions are defined as:

$$\phi_{t}=G_{t}\left(G_{0}\left(\mu_{PM},\sigma_{PM}^{2}\right),G_{0}\left(\mu_{PS},\sigma_{PS}^{2}\right)\right)$$ (4)

$$\zeta_{t}=\left|G_{t}\left(G_{0}\left(\mu_{TM},\sigma_{TM}^{2}\right),G_{0}\left(\mu_{TS},\sigma_{TS}^{2}\right)\right)\right|$$ (5)

Hence, the translational and turn speeds for each cell at each time step *t* are drawn from Gaussian distributions, indicated by *G*_*t*_, the mean and standard deviations of which were themselves drawn from Gaussian distributions once only at time zero, indicated by *G*_0_, and then maintained for each cell throughout the simulation.

#### Inverse homogeneous- and heterogeneous-correlated random walk (IHomoCRW and IHeteroCRW)

These random walks differ from HomoCRW and HeteroCRW (respectively) in that the turn speed magnitude is inversely correlated with the translational speed as follows:

$$\zeta_{t}=\left|G_{t}\left(\_,\_\right)\right|$$ (6)

$$\phi_{t}=G_{t}\left(\_,\_\right)\cdot \left(\frac{\left(\zeta_{max}^{\beta }-\zeta_{t}^{\beta }\right)}{\zeta_{max}^{\beta }}\right)$$ (7)

Where ‘_’ indicates no change from the previous HomoCRW/HeteroCRW model formulations. *ζ*_*max*_ represents the maximum translational speed, determined empirically *in vivo* as 25*μ*m/minute. As a cell’s translational speed (*ζ*_*t*_) approaches *ζ*_*max*_, the turn speed (*ϕ*_*t*_) is multiplied by a factor approaching zero. Conversely, a cell that is translationally stationary will see no reduction in its turn speed. *β* describes this relationship, see supplementary S34 Fig. For *β* = 1, pitch speeds are scaled linearly with translation speeds. For values of *β* >1, higher translational speeds can be accommodated before substantial reductions in pitch speed occur, and vice versa for *β* <1.

### Cellular Motility Profiling

Both simulated and *in vivo* data undergo the same motility analysis, based on time series tracked cell spatial locations sampled every Δ*t* seconds. For each time point *t*_*i*_, the vector describing the movement of a cell to its current location is calculated, and termed *d*_*i*_. The displacement and translational speed over vector *d*_*i*_ are calculated. A cell’s turn speed at time *t*_*i*_ is calculated as the angle between vectors *d*_*i*+1_ and *d*_*i*_ divided by Δ*t*.

The largest measurable turn angle is 180°, and conversion into turn speeds (°/min) depends on the time step. Simulation time steps, 30s for T cells and 45s for neutrophils, correspond with maximum turn speeds of 360 and 240°/min respectively. These figures match the maximum discernible turn speeds for the *in vivo* datasets. However, the maximum discernible turn speed for each experiment within a dataset will differ with the time step (see S1 Table), and this could represent an artifact for our calibration experiments. Given the majority of recorded turn speeds lie well below the maximum values (S1B Fig) we believe the influence of this discrepancy on calibration experiments to be minor, however we acknowledge its existence.

A cell’s meandering index is defined as the net displacement from its first to last observed locations divided by its total distance traveled. This yields a value between 0 and 1, respectively indicating the extremes of a cell finishing where it started or traveling in a straight line. Cells with total displacements <27*μ*m are excluded from the analysis to avoid artifacts introduced by the sessile contaminating cell types such as dendritic cells, or cells that are dead or dying. This same displacement threshold is also applied to simulation data to ensure fair comparisons. The figure of 27*μ*m was derived empirically using Imaris software, and represents an optimal trade-off for removing unwanted artifacts whilst minimizing the exclusion of motile T cells and neutrophils.

The motility profile for a dataset, which typically constitutes several replicate experiments, comprises the following metrics. All translational speeds for all cells are pooled together to form one distribution. A similar pool of all cell turn speeds is constructed. All cell meandering indexes are pooled together into one distribution. Only these three metrics are used in simulation-based motility model calibration and evaluation.

The following additional metrics are also derived, but not used in calibration or evaluation. We construct distributions of median track translation and turn speeds. Mean squared displacement (MSD) over time interval plots are produced. Displacement data for a given time interval is extracted from anywhere in the time-series, i.e., time intervals are not absolute from time zero. Time intervals of 0 to 25% of the maximum track length are investigated. Slopes for MSD plots are calculated using linear regression. Displacement autocorrelation was calculated as in [21].

### Calibration Process and Preventing Over-Fitting

Each of the six models is implemented in simulation in turn, and then independently calibrated against each of the *in vivo* datasets. Calibration is performed using NSGA-II [22], a multi-objective optimization algorithm based on a genetic algorithm that uses Pareto fronts to track candidate solutions representing the best trade-offs found to date with respect to each objective. NSGA-II is an elitist algorithm, meaning that a subsequent generation’s population is composed of the best solutions found to date: the solutions comprising the Pareto front. If the Pareto front comprises more members than the population size, a subset composed of those Pareto members having the largest fitness differences between their immediate neighbours summed for all objectives is selected, a strategy intended to promote full coverage of the Pareto front. If the Pareto front comprises fewer members than the population size then members of the next front (those dominated by only one other solution) are selected in the same manner, and so on until the entire population has been selected. New solutions are generated through blended crossover of their two parents, coupled with Gaussian mutation using the standard normal distribution. These evolutionary operators correspond to the *Inspyred* python package implementation of NSGA-II. For further details on NSGA-II we refer to the reader to [22].

Candidate solutions represent putative model parameters. Evaluation of a solution entails executing ten replicate simulations with the parameters it represents, and generating a motility profile from the pooled results. This motility profile is contrasted with that of the *in vivo* dataset: the Kolmogorov-Smirnov (KS) difference between the motility profiles’ distributions of cell translation (S1A Fig) and turn speeds (S1B Fig), and meandering indices (S1C Fig) together form three objectives. A perfect simulation representation of an *in vivo* data set would yield a KS value of 0 for each objective. In reality, no random walk model, by virtue of being an abstract model, will likely achieve this. Instead, some disparity in at least one metric will exist. The use of Pareto fronts accommodates trade-offs between metrics; two solutions are Pareto-equivalent if neither provides better alignments with *in vivo* data across all measures.

An individual calibration is performed for a maximum of 40 generations of the genetic algorithm, for all models. Calibration is terminated before 40 generations only if over-fitting, as described below, is detected. The number of candidates in each generation is scaled with the number of model parameters, thereby reflecting the complexity of the problem, as shown in Table 4:

**Table 4:** The number of parameters in each motility model, and number of candidates maintained in each NSGA-II generation whilst calibrating them.

Model	Parameters	Candidates per generation
Brownian Motion	1	20
Lévy Walk	4	50
HomoCRW	4	50
HeteroCRW	8	80
IHomoCRW	5	60
IHeteroCRW	9	100

We avoid over-fitting models, wherein calibrated solutions represent the nuanced stochastic-sampling-derived features of the data rather than its general qualities, by dividing *in vivo* datasets into training (70% of cell tracks) and validation sets (30%), as is standard machine learning practice [37]. Each putative model parameter set is independently evaluated against both training and validation datasets, and two Pareto fronts, representing the best solutions found with respect to each, are maintained throughout calibration. Progression of candidate solutions through subsequent generations is determined through performance against the training dataset alone. The over-fittedness of the population is defined as the proportion of training dataset Pareto front solutions that are not also members of the validation dataset Pareto front. Calibration is stopped when either the maximum number of generations have been run, or the over-fitted metric >0.8.

The model assessments reported here are made on the basis of validation dataset Pareto front solutions. We note that in no cases were any calibration efforts terminated prematurely on the basis of over-fitting, but over-fitted scores of around 0.6 were not uncommon.

### Contrasting Random Walk Models

Calibration produces a Pareto front comprising those parameter values yielding the best reflections of the *in vivo* dataset. By contrasting Pareto fronts produced by two different models, that which is most capable of reproducing the motility of *in vivo* cells is ascertained. For a given model and *in vivo* dataset (T cell or neutrophil), calibration is performed three times. One overarching Pareto front is then generated from the best solutions generated under each exercise, and is used in model evaluation.

Three complementary analyses are performed when contrasting two models. First, the proportion of each models’ front that is non-Pareto-dominated by the other is calculated. If two models are exactly equal in their capture of the biology across all objectives, then these values should be 100% for each. If the two values are equal, but not 100%, then the models are still considered equal reflections of the biology overall, but they differ in how well they reflect particular objectives. Pareto front sizes are reported alongside these proportions, to highlight where high or low values simply reflect fronts containing few or many solutions.

Second, we contrast the best (lowest) 30 Λ values found within a Pareto front using the Kolmogorov-Smirnov statistic (Fig 3). The Λ function, defined below for a candidate *m*, delivers low values to solutions having low mean objective KS values with small variance. Hence, it selects those solutions that perform well, and equally well, on all objectives.

$$\Lambda \left(m\right)=\alpha \cdot \bar{KS}{\left(m\right)}^{2}+\sum_{o\in \Omega }{\left(KS_{o}\left(c\right)-\bar{KS}\left(m\right)\right)}^{2}$$ (8)

$\bar{KS}\left(m\right)$ represents the mean objective KS score for member *m*, Ω represents the set of objectives and *KS*_*o*_(*m*) represents the KS scores for member *m* against objective *o*. The coefficient *α* can be used to prioritize mean or variance terms, a problem specific decision; a value of *α* = 1 is used throughout this manuscript. S35 Fig depicts how Λ values vary in a hypothetical scenario comprising two objectives, under different values of *α*.

Lastly, the distribution of scores for each objective generated under each Pareto front are contrasted, thereby highlighting how well each model captures each motility characteristic. These are shown in Figs 4 and 5. The distributions are plotted on the left of these figures and are statistically contrasted using the Kolmogorov-Smirnov statistic, the values of which are given in the tables on the right of these figures.

### Calibrating IHeteroCRW with MSD

Experiments where the meandering index was replaced with mean squared displacement (MSD) as an objective for multi-objective optimisation used the same experimental setup as reported above. The MSD calibration objective operates by taking the absolute difference between the MSD linear regression slopes generated for candidate solution and neutrophil dataset as reported above. Two remaining calibration objectives are constructed from KS statistics applied to pooled translational and turn speed data, as reported above. Calibration was performed three independent time using 100 candidates for 40 generations, with an overfitting termination threshold of 0.8.

The best solution from the MSD-based calibration exercise, reported in S31 Fig, is that with the lowest sum of objective values. The Λ function described above is inappropriate in this context, as the MSD objective is not based on the KS statistic. Hence, is it nonsensical to take their mean value.

### Software

The 3-dimensional continuous space simulation is written in Java, using the MASON simulation framework library [42]. We use the *Inspyred* implementation of NSGA-II, written in Python, to perform calibration. Kolmogorov-Smirnov statistics, and their associated p-values, are determined using Python’s *scipy.stats.ks_2samp* module. The statistical modeling of cellular translation and turn speed dynamics was performed using python, and its *numpy* and *scipy* packages. The 3D agent-based simulation and multi-objective optimisation software we developed for this manuscript is distributed under version 3 of the GNU General Public License in the S1 Software ZIP file (the third party libraries we employ will need to be acquired separately from their respective sources for licensing reasons).

## Supporting Information

S1 Table The durations and time-intervals in time-series data of *in vivo* T cell and neutrophil experiments against which calibration is performed. (PNG)

S1 Fig Characterization and comparison of T cell and neutrophil datasets. (A) All cellular translational speeds across all time points in all imaging experiments pooled together. (B) Similarly for turn speeds. Mean squared displacement (MSD) over time plots, on log-log axes, for T cells (C) and neutrophils (D). The time axis represents a given duration occurring anywhere across the temporal domain (not absolute time since t0). Grey lines represent MSD plots for each individual imaging experiment. Red lines indicate the gradient resulting from linear regression on all data from all imaging experiments. (E) Cell meandering indices. (F) The number of recorded positions (number of observations) for each track comprising each dataset. A, B, E and F are presented as cumulative distribution plots, wherein the y-axis describes the proportion of data less than or equal to the corresponding x-axis value. Kolmogorov-Smirnov (KS) values are given, as are their associated p-values. Only the metrics depicted in panels A, B and E are used as objectives in simulation-based motility model assessment experiments.
(PNG)

S2 Fig Further characterisation of T cell and neutrophil datasets. Scatter plots showing track meandering indexes against track durations, for T cells (A) and neutrophils (B). There exists a bias for higher meandering indexes in shorter duration tracks; this has been quantified using Spearmans’ rank correlation coefficient (rho). Representative tracks are shown for T cell (C) and neutrophil (D) datasets. Fourty tracks in each are selected to sample at regular intervals the full distribution of track displacements. Track positions relative to starting points are shown.
(PNG)

S3 Fig Faster, more directional cells are observed a fewer number of times, as they more rapidly leave the imaging volume. Scatter plots of T cell median track translation (A) and turn (C) speeds against the number of times each track was observed. Similar plots for neutrophils are shown in (B) and (D) respectively. Spearman’s rank correlation coefficients (rho) and associated p-values are given.
(PNG)

S4 Fig Graphical overview of our method for fitting statistical distributions to track translation and turn speed dynamics. For brevity, the method is described as applying to translational speed data, however the same method is separately applied to turn speed data also.
(PNG)

S5 Fig Overview of homogeneous and heterogeneous statistical distributions. These distributions are fitted to *in vivo* cell translational and turn speed data to ascertain that cells are inherently heterogeneous in their motility characteristics. They are also used in designing random walk models subject to 3D agent-based simulation.
(PNG)

S6 Fig Fitting statistical distributions to *in vivo* T cell pooled translational speeds. Each distribution was fitted to *in vivo* data five independent times, the resultant distribution parameters are given.
(PNG)

S7 Fig Fitting statistical distributions to *in vivo* neutrophil pooled translational speeds. Each distribution was fitted to *in vivo* data five independent times, the resultant distribution parameters are given.
(PNG)

S8 Fig The best alignments of *in vivo* and statistically modeled median track translation and turn speed distributions. Each statistical distribution was fitted 5 independent times against pooled translational (or turn) speed distributions. Thereafter, each fitted distribution was used to generate 100 additional datasets using the method summarized in S4 Fig, constituting 500 for each model. Each of these 500 datasets’ median track characteristics were then contrasted with corresponding *in vivo* data. The best of those 500 alignments, as measured through the Kolmogorov-Smirnov statistic, are shown.
(PNG)

S9 Fig Fitting statistical distributions to *in vivo* T cell pooled turn speeds. Each distribution was fitted to *in vivo* data five independent times, the resultant distribution parameters are given.
(PNG)

S10 Fig Fitting statistical distributions to *in vivo* neutrophil pooled turn speeds. Each distribution was fitted to *in vivo* data five independent times, the resultant distribution parameters are given.
(PNG)

S11 Fig Summary of how Pareto fronts are used in assessing and contrasting putative random walk models. (PNG)

S12 Fig Alignment of best simulated Brownian motion solution with T cell data. The best solution is that with the lowest Λ value. Pooled (A) and median track (B) translational speed distributions are shown as cumulative distribution plots. Similar plots, (C) and (D), depict turn speed data. (E) Cumulative distribution plot of track meandering index distributions. (F) Mean squared displacements for given durations (anywhere in the temporal domain, not from time zero only) plotted on log-log axes. The gradients of linear regression fitted models are given. (G) X and Y coordinates relative to starting positions of 7 tracks. (H) Scatter plot of track meandering indices against duration, Spearman’s rank correlation coefficient is given. The model’s parameter value is given. Brownian motion is a poor reflection of T cell motility dynamics: following removal of tracks <27*μ*m net displacement, which is applied to *in vivo* data also, only 7 simulated tracks remain. We note that model calibration was performed using metrics of panels A, C and E only.
(PNG)

S13 Fig Alignment of best simulated Lévy walk solution with T cell data. The best solution is that with the lowest Λ value. Pooled (A) and median track (B) translational speed distributions are shown as cumulative distribution plots. Similar plots, (C) and (D), depict turn speed data. (E) Cumulative distribution plot of track meandering index distributions. (F) Mean squared displacements for given durations (anywhere in the temporal domain, not from time zero only) plotted on log-log axes. The gradients of linear regression fitted models are given. (G) X and Y coordinates relative to starting positions of 40 tracks, selected to capture the entire range of net displacements. (H) Scatter plot of track meandering indices against duration, Spearman’s rank correlation coefficient is given. The model’s parameter values are given. We note that model calibration was performed using metrics of panels A, C and E only.
(PNG)

S14 Fig Alignment of best simulated homogeneous CRW solution with T cell data. The best solution is that with the lowest Λ value. Pooled (A) and median track (B) translational speed distributions are shown as cumulative distribution plots. Similar plots, (C) and (D), depict turn speed data. (E) Cumulative distribution plot of track meandering index distributions. (F) Mean squared displacements for given durations (anywhere in the temporal domain, not from time zero only) plotted on log-log axes. The gradients of linear regression fitted models are given. (G) X and Y coordinates relative to starting positions of 40 tracks, selected to capture the entire range of net displacements. (H) Scatter plot of track meandering indices against duration, Spearman’s rank correlation coefficient is given. The model’s parameter values are given. We note that model calibration was performed using metrics of panels A, C and E only.
(PNG)

S15 Fig Alignment of best simulated heterogeneous CRW solution with T cell data. The best solution is that with the lowest Λ value. Pooled (A) and median track (B) translational speed distributions are shown as cumulative distribution plots. Similar plots, (C) and (D), depict turn speed data. (E) Cumulative distribution plot of track meandering index distributions. (F) Mean squared displacements for given durations (anywhere in the temporal domain, not from time zero only) plotted on log-log axes. The gradients of linear regression fitted models are given. (G) X and Y coordinates relative to starting positions of 40 tracks, selected to capture the entire range of net displacements. (H) Scatter plot of track meandering indices against duration, Spearman’s rank correlation coefficient is given. The model’s parameter values are given. We note that model calibration was performed using metrics of panels A, C and E only.
(PNG)

S16 Fig Alignment of best simulated inverse homogeneous CRW solution with T cell data. The best solution is that with the lowest Λ value. Pooled (A) and median track (B) translational speed distributions are shown as cumulative distribution plots. Similar plots, (C) and (D), depict turn speed data. (E) Cumulative distribution plot of track meandering index distributions. (F) Mean squared displacements for given durations (anywhere in the temporal domain, not from time zero only) plotted on log-log axes. The gradients of linear regression fitted models are given. (G) X and Y coordinates relative to starting positions of 40 tracks, selected to capture the entire range of net displacements. (H) Scatter plot of track meandering indices against duration, Spearman’s rank correlation coefficient is given. The model’s parameter values are given. We note that model calibration was performed using metrics of panels A, C and E only.
(PNG)

S17 Fig Alignment of best simulated inverse heterogeneous CRW solution with T cell data. The best solution is that with the lowest Λ value. Pooled (A) and median track (B) translational speed distributions are shown as cumulative distribution plots. Similar plots, (C) and (D), depict turn speed data. (E) Cumulative distribution plot of track meandering index distributions. (F) Mean squared displacements for given durations (anywhere in the temporal domain, not from time zero only) plotted on log-log axes. The gradients of linear regression fitted models are given. (G) X and Y coordinates relative to starting positions of 40 tracks, selected to capture the entire range of net displacements. (H) Scatter plot of track meandering indices against duration, Spearman’s rank correlation coefficient is given. The model’s parameter values are given. We note that model calibration was performed using metrics of panels A, C and E only.
(PNG)

S18 Fig Alignment of best simulated Brownian motion solution with neutrophil data. The best solution is that with the lowest Λ value. Pooled (A) and median track (B) translational speed distributions are shown as cumulative distribution plots. Similar plots, (C) and (D), depict turn speed data. (E) Cumulative distribution plot of track meandering index distributions. (F) Mean squared displacements for given durations (anywhere in the temporal domain, not from time zero only) plotted on log-log axes. The gradients of linear regression fitted models are given. (G) X and Y coordinates relative to starting positions of 40 tracks, selected to capture the entire range of net displacements. (H) Scatter plot of track meandering indices against duration, Spearman’s rank correlation coefficient is given. The model’s parameter values are given. We note that model calibration was performed using metrics of panels A, C and E only.
(PNG)

S19 Fig Alignment of best simulated Lévy walk solution with neutrophil data. The best solution is that with the lowest Λ value. Pooled (A) and median track (B) translational speed distributions are shown as cumulative distribution plots. Similar plots, (C) and (D), depict turn speed data. (E) Cumulative distribution plot of track meandering index distributions. (F) Mean squared displacements for given durations (anywhere in the temporal domain, not from time zero only) plotted on log-log axes. The gradients of linear regression fitted models are given. (G) X and Y coordinates relative to starting positions of 40 tracks, selected to capture the entire range of net displacements. (H) Scatter plot of track meandering indices against duration, Spearman’s rank correlation coefficient is given. The model’s parameter values are given. We note that model calibration was performed using metrics of panels A, C and E only.
(PNG)

S20 Fig Alignment of best simulated homogeneous CRW solution with neutrophil data. The best solution is that with the lowest Λ value. Pooled (A) and median track (B) translational speed distributions are shown as cumulative distribution plots. Similar plots, (C) and (D), depict turn speed data. (E) Cumulative distribution plot of track meandering index distributions. (F) Mean squared displacements for given durations (anywhere in the temporal domain, not from time zero only) plotted on log-log axes. The gradients of linear regression fitted models are given. (G) X and Y coordinates relative to starting positions of 40 tracks, selected to capture the entire range of net displacements. (H) Scatter plot of track meandering indices against duration, Spearman’s rank correlation coefficient is given. The model’s parameter values are given. We note that model calibration was performed using metrics of panels A, C and E only.
(PNG)

S21 Fig Alignment of best simulated heterogeneous CRW solution with neutrophil data. The best solution is that with the lowest Λ value. Pooled (A) and median track (B) translational speed distributions are shown as cumulative distribution plots. Similar plots, (C) and (D), depict turn speed data. (E) Cumulative distribution plot of track meandering index distributions. (F) Mean squared displacements for given durations (anywhere in the temporal domain, not from time zero only) plotted on log-log axes. The gradients of linear regression fitted models are given. (G) X and Y coordinates relative to starting positions of 40 tracks, selected to capture the entire range of net displacements. (H) Scatter plot of track meandering indices against duration, Spearman’s rank correlation coefficient is given. The model’s parameter values are given. We note that model calibration was performed using metrics of panels A, C and E only.
(PNG)

S22 Fig Alignment of best simulated inverse homogeneous CRW solution with neutrophil data. The best solution is that with the lowest Λ value. Pooled (A) and median track (B) translational speed distributions are shown as cumulative distribution plots. Similar plots, (C) and (D), depict turn speed data. (E) Cumulative distribution plot of track meandering index distributions. (F) Mean squared displacements for given durations (anywhere in the temporal domain, not from time zero only) plotted on log-log axes. The gradients of linear regression fitted models are given. (G) X and Y coordinates relative to starting positions of 40 tracks, selected to capture the entire range of net displacements. (H) Scatter plot of track meandering indices against duration, Spearman’s rank correlation coefficient is given. The model’s parameter values are given. We note that model calibration was performed using metrics of panels A, C and E only.
(PNG)

S23 Fig Alignment of best simulated inverse heterogeneous CRW solution with neutrophil data. The best solution is that with the lowest Λ value. Pooled (A) and median track (B) translational speed distributions are shown as cumulative distribution plots. Similar plots, (C) and (D), depict turn speed data. (E) Cumulative distribution plot of track meandering index distributions. (F) Mean squared displacements for given durations (anywhere in the temporal domain, not from time zero only) plotted on log-log axes. The gradients of linear regression fitted models are given. (G) X and Y coordinates relative to starting positions of 40 tracks, selected to capture the entire range of net displacements. (H) Scatter plot of track meandering indices against duration, Spearman’s rank correlation coefficient is given. The model’s parameter values are given. We note that model calibration was performed using metrics of panels A, C and E only.
(PNG)

S24 Fig Alignments of each simulated motility models’ Pareto front solutions’ median track translational and turn speed distributions with *in vivo* data. Each motility model was independently simulated and calibrated against both T cell and neutrophil data 3 times. Pareto fronts were compiled for each model from the solutions in all three calibrations. The alignment of each Pareto front solution median track translation/turn distribution with the corresponding *in vivo* data was assessed using the Kolmogorov-Smirnov (KS) statistic. Shown here are the distributions of KS values across all Pareto front solutions. These data broadly correspond with the independent statistical modeling of translation and turn speed dynamics, Fig 1. Heterogeneous CRW models better capture T cell and neutrophil dynamics than homogeneous CRW models, with the exception of neutrophil median track turn speeds, where no discernible difference is found.
(PNG)

S25 Fig HeteroCRW and HomoCRW Pareto fronts plotted against each objective KS value. Calibration was against T cell data (top), or neutrophil data (bottom). ‘Trans’, translation speed KS values; ‘Turn’, turn speed KS values; ‘MI’, meandering index KS values. The large arrow identifies the origin, which represent a perfect reproduction of *in vivo* motility dynamics. Faded color dots lie further from the viewer. Plots have been rotated to emphasize the separation between the two Pareto fronts.
(PNG)

S26 Fig IHeteroCRW and IHomoCRW Pareto fronts plotted against each objective KS value. Calibration was against T cell data (top), or neutrophil data (bottom). ‘Trans’, translation speed KS values; ‘Turn’, turn speed KS values; ‘MI’, meandering index KS values. The large arrow identifies the origin, which represent a perfect reproduction of *in vivo* motility dynamics. Plots have been rotated to emphasize the separation between the two Pareto fronts.
(PNG)

S27 Fig Correlations between translation and turn speeds for various CRW models. IHomoCRW and IHeteroCRW explicitly prescribe a negative correlation between translation and turn speeds, motivated by finding such a correlation in our *in vivo* data. Data shown represent the best solutions, as determined by lowest Λ value, for each model when calibrated against each dataset.
(PNG)

S28 Fig Homo and IHomoCRW Pareto fronts plotted against each objective KS value. Calibration was against T cell data (top), or neutrophil data (bottom). ‘Trans’, translation speed KS values; ‘Turn’, turn speed KS values; ‘MI’, meandering index KS values. The large arrow identifies the origin, which represent a perfect reproduction of *in vivo* motility dynamics. Faded color dots lie further from the viewer. Plots have been rotated to emphasize the separation between the two Pareto fronts.
(PNG)

S29 Fig HeteroCRW and IHeteroCRW Pareto fronts plotted against each objective KS value. Calibration was against T cell data (top), or neutrophil data (bottom). ‘Trans’, translation speed KS values; ‘Turn’, turn speed KS values; ‘MI’, meandering index KS values. The large arrow identifies the origin, which represent a perfect reproduction of *in vivo* motility dynamics. Plots have been rotated to emphasize the separation between the two Pareto fronts.
(PNG)

S30 Fig Comparison of using meandering index or mean squared displacement (MSD) as an objective. The IHeteroCRW model was calibrated against neutrophil data, using either the meandering index or the MSD as a calibration objective. The MSD objective comprises the absolute difference between linear-regression fitted gradients applied to neutrophil and IHeteroCRW candidate solution data. The pooled translational and turn speeds were used as the other two objectives. Calibration was performed three independent times in both cases, shown are the performances of Pareto front solutions of all three calibrations pooled together.
(PNG)

S31 Fig Alignment of best simulated inverse heterogeneous CRW solution with neutrophil data, when using MSD instead of the meandering index. The best solution is that with the lowest Λ value. Pooled (A) and median track (B) translational speed distributions are shown as cumulative distribution plots. Similar plots, (C) and (D), depict turn speed data. (E) Cumulative distribution plot of track meandering index distributions. (F) Mean squared displacements for given durations (anywhere in the temporal domain, not from time zero only) plotted on log-log axes. The gradients of linear regression fitted models are given. (G) Displacement autocorrelations for the given time intervals; dots represent median values with error bars covering data lying within the interquartile range at each time interval. (H) Scatter plot of track meandering indices against duration, Spearman’s rank correlation coefficient is given. The model’s parameter values are given. Note that model calibration was performed using metrics of panels A, B and F only. See Methods for more details.
(PNG)

S32 Fig Displacement autocorrelations for given time intervals, for T cell *in vivo* and modeled data. Dots represent median values, with error bars covering data lying within the interquartile range at each time interval. Irregular intervals for *in vivo* data reflect imaging experiments with differing time-steps. For methodological details see [21]. The CRW models best reflect the *in vivo* dynamics.
(PNG)

S33 Fig Displacement autocorrelations for given time intervals, for neutrophil *in vivo* and modeled data. Dots represent median values, with error bars covering data lying within the interquartile range at each time interval. Irregular intervals for *in vivo* data reflect imaging experiments with differing time-steps. For methodological details see [21]. The CRW models best reflect the *in vivo* dynamics.
(PNG)

S34 Fig *β* determines the relationship between the factor by which turn angles are scaled according to a cell’s translational speed. It is a parameter of the IHomoCRW and IHeteroCRW models.
(PNG)

S35 Fig Landscape of Λ values for two objectives. *α* values of (top) 0.5, (middle) 1.0 and (bottom) 5.0 are shown. The black line indicates *y* = *x*. Note that the range of Λ values changes with *α*, and hence comparisons between solution and Pareto front Λ values are valid only when generated using the same *α* value.
(PNG)

S1 Algorithm Pseudocode for the algorithm through which a given statistical model is used to produce a modeled cellular translation/turn dataset. The dataset produced contains the same number of data items, spread across the same number of tracks each with the same number of observations as the *in vivo* dataset. The algorithm accounts for the time-step duration of each *in vivo* track (which differed between imaging experiments) when adjusting the maximum recognizable turn speed (does not apply for translational data). The negative correlation between track duration and median track translational speed, and the positive correlation between track duration and median track turn speed, as found in the *in vivo* datasets are maintained.
(PNG)

S1 Software The 3D agent-based simulation, statistical distribution fitting and multi-objective optimisation software developed for this manuscript. (ZIP)
