# Supplementary figures and images for: Leukocyte Motility Models Assessed through Simulation and Multi-objective Optimization-Based Model Selection
Source: PLoS Comput Biol. 2016 Sep 2;12(9):e1005082. doi: 10.1371/journal.pcbi.1005082 (PMC5010290; doi:10.1371/journal.pcbi.1005082)

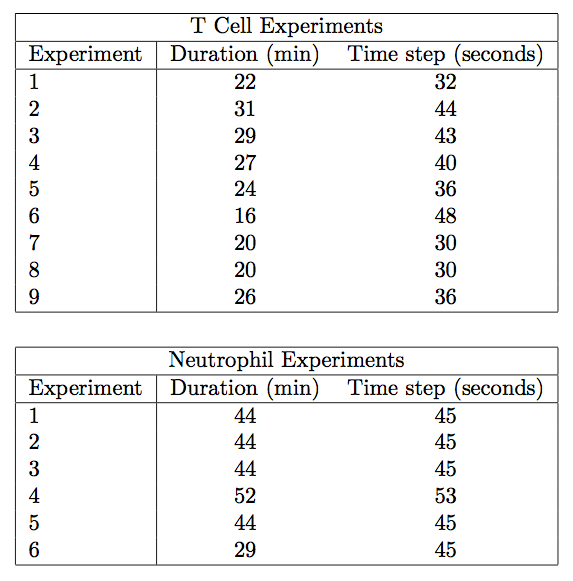

Supplement: S1 Table — (PNG) [file pcbi.1005082.s001.png]

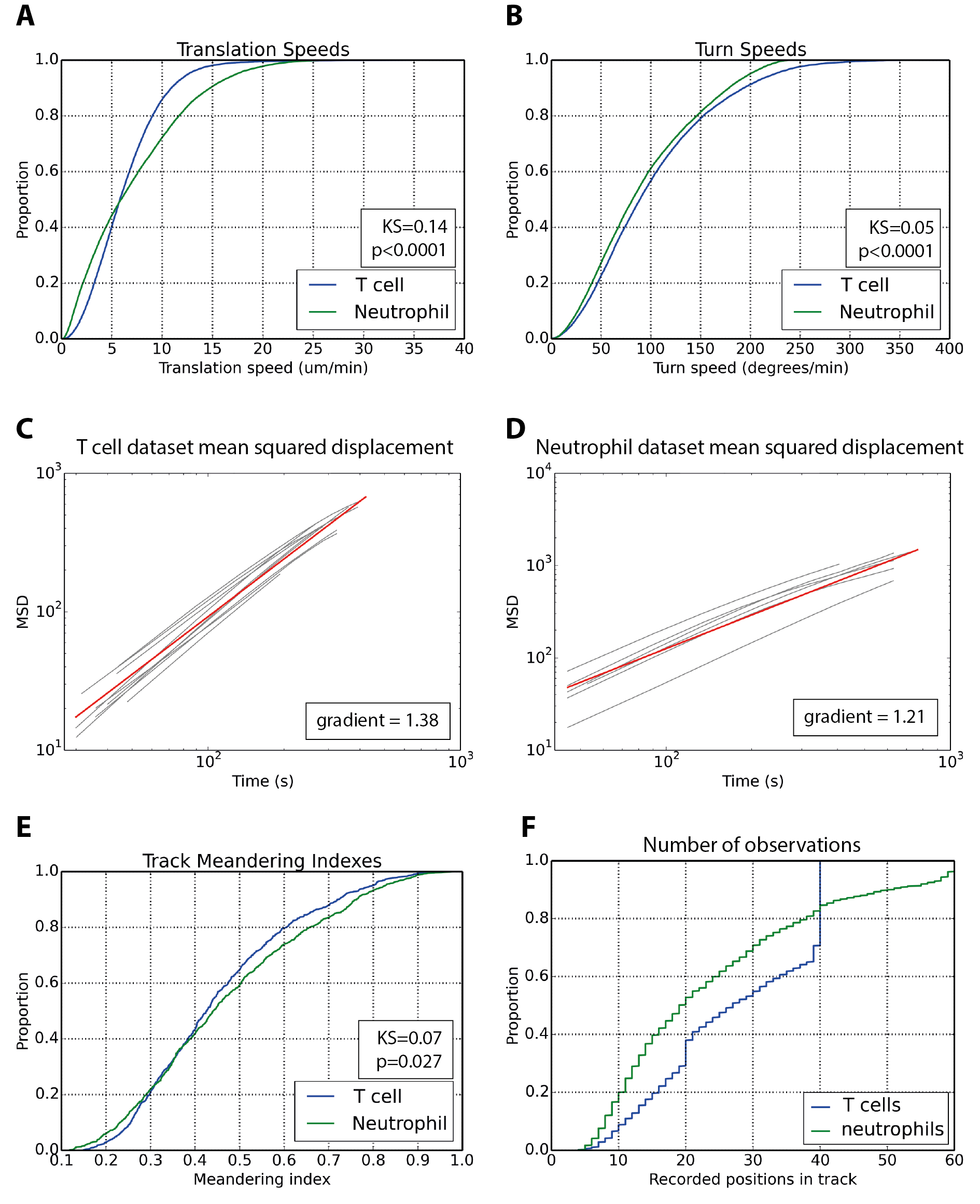

Supplement: S1 Fig — (A) All cellular translational speeds across all time points in all imaging experiments pooled together. (B) Similarly for turn speeds. Mean squared displacement (MSD) over time plots, on log-log axes, for T cells (C) and neutrophils (D). The time axis represents a given duration occurring anywhere across the temporal domain (not absolute time since t0). Grey lines represent MSD plots for each individual imaging experiment. Red lines indicate the gradient resulting from linear regression on all data from all imaging experiments. (E) Cell meandering indices. (F) The number of recorded positions (number of observations) for each track comprising each dataset. A, B, E and F are presented as cumulative distribution plots, wherein the y-axis describes the proportion of data less than or equal to the corresponding x-axis value. Kolmogorov-Smirnov (KS) values are given, as are their associated p-values. Only the metrics depicted in panels A, B and E are used as objectives in simulation-based motility model assessment experiments. (PNG) [file pcbi.1005082.s002.png]

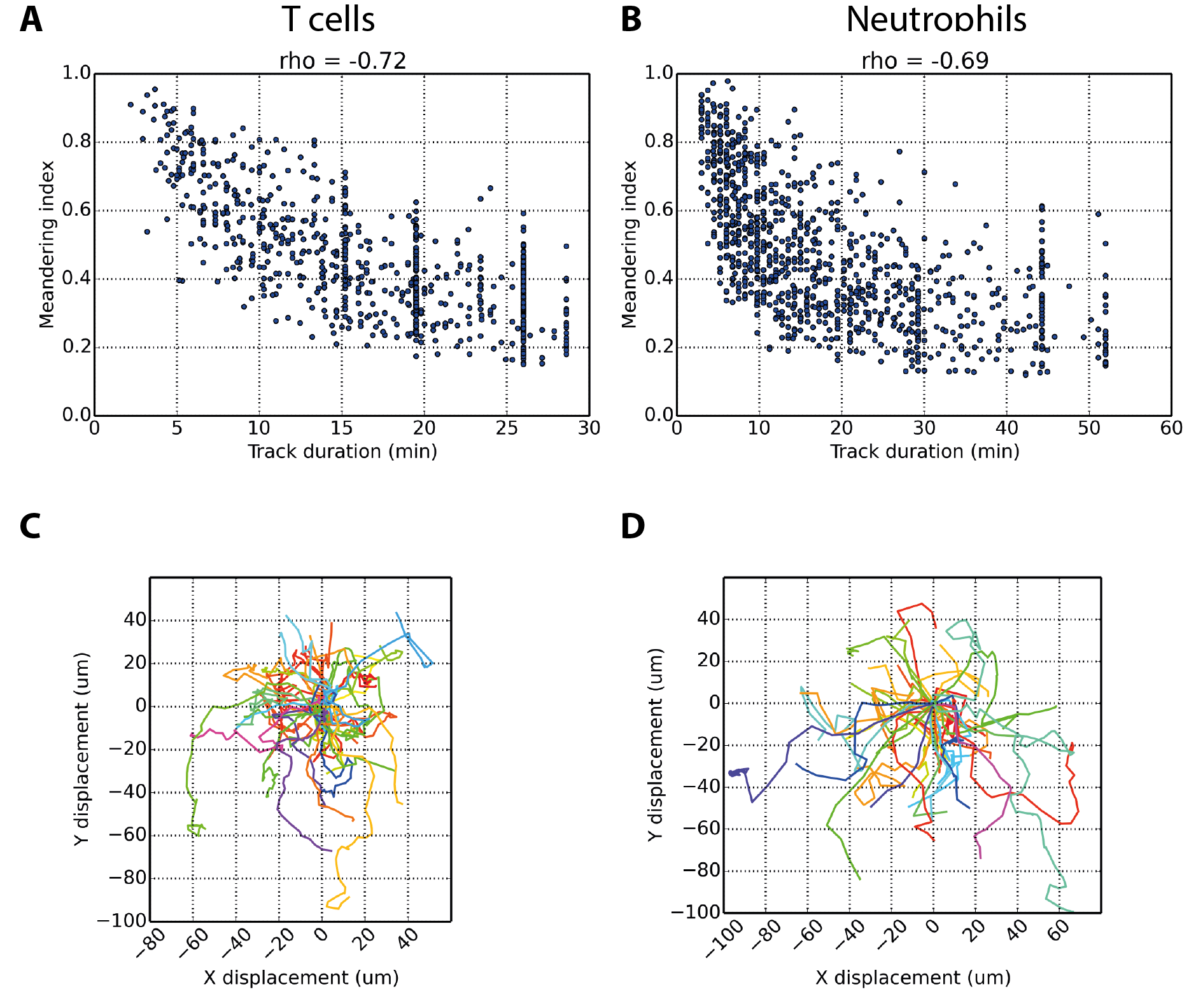

Supplement: S2 Fig — Scatter plots showing track meandering indexes against track durations, for T cells (A) and neutrophils (B). There exists a bias for higher meandering indexes in shorter duration tracks; this has been quantified using Spearmans’ rank correlation coefficient (rho). Representative tracks are shown for T cell (C) and neutrophil (D) datasets. Fourty tracks in each are selected to sample at regular intervals the full distribution of track displacements. Track positions relative to starting points are shown. (PNG) [file pcbi.1005082.s003.png]

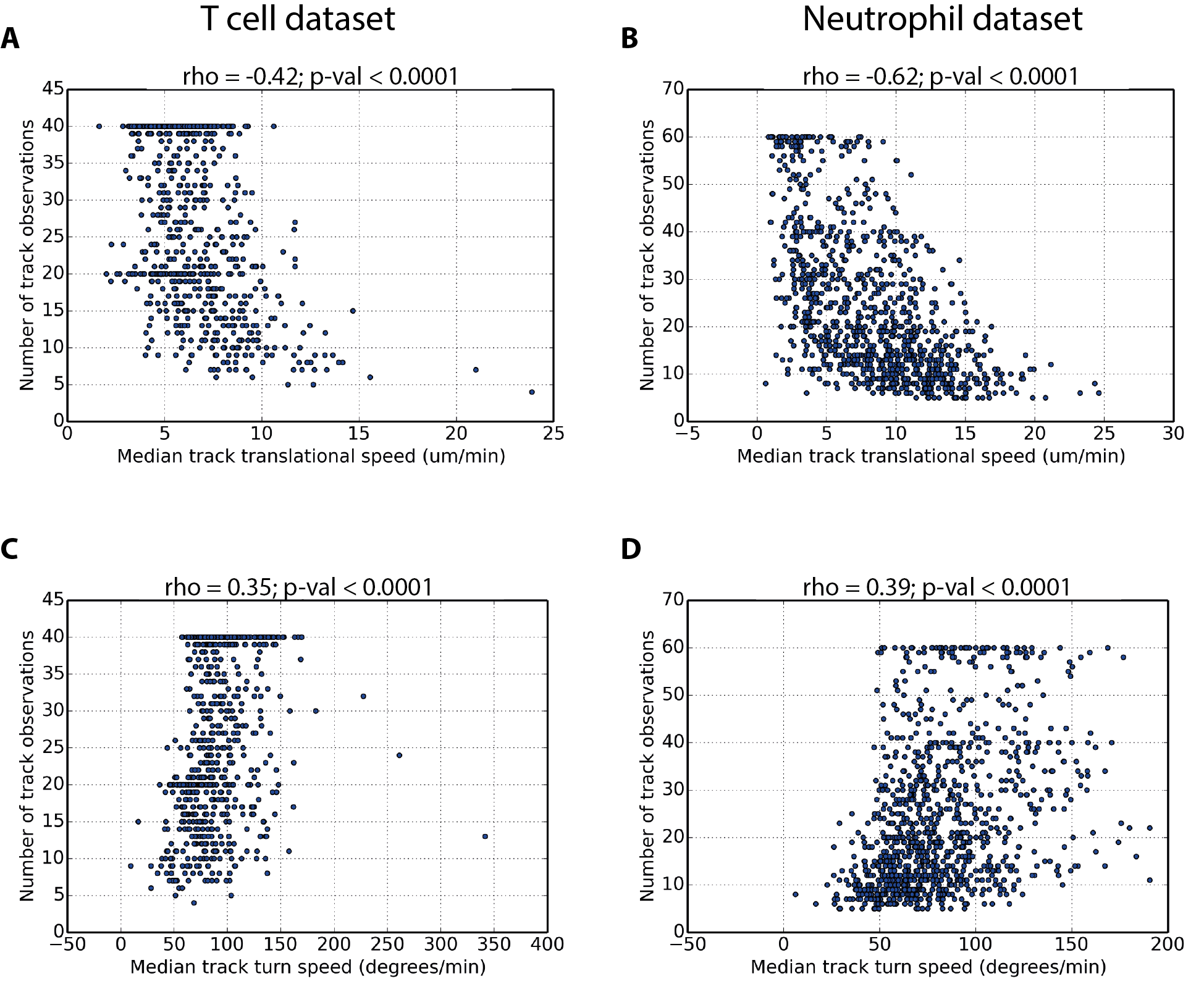

Supplement: S3 Fig — Scatter plots of T cell median track translation (A) and turn (C) speeds against the number of times each track was observed. Similar plots for neutrophils are shown in (B) and (D) respectively. Spearman’s rank correlation coefficients (rho) and associated p-values are given. (PNG) [file pcbi.1005082.s004.png]

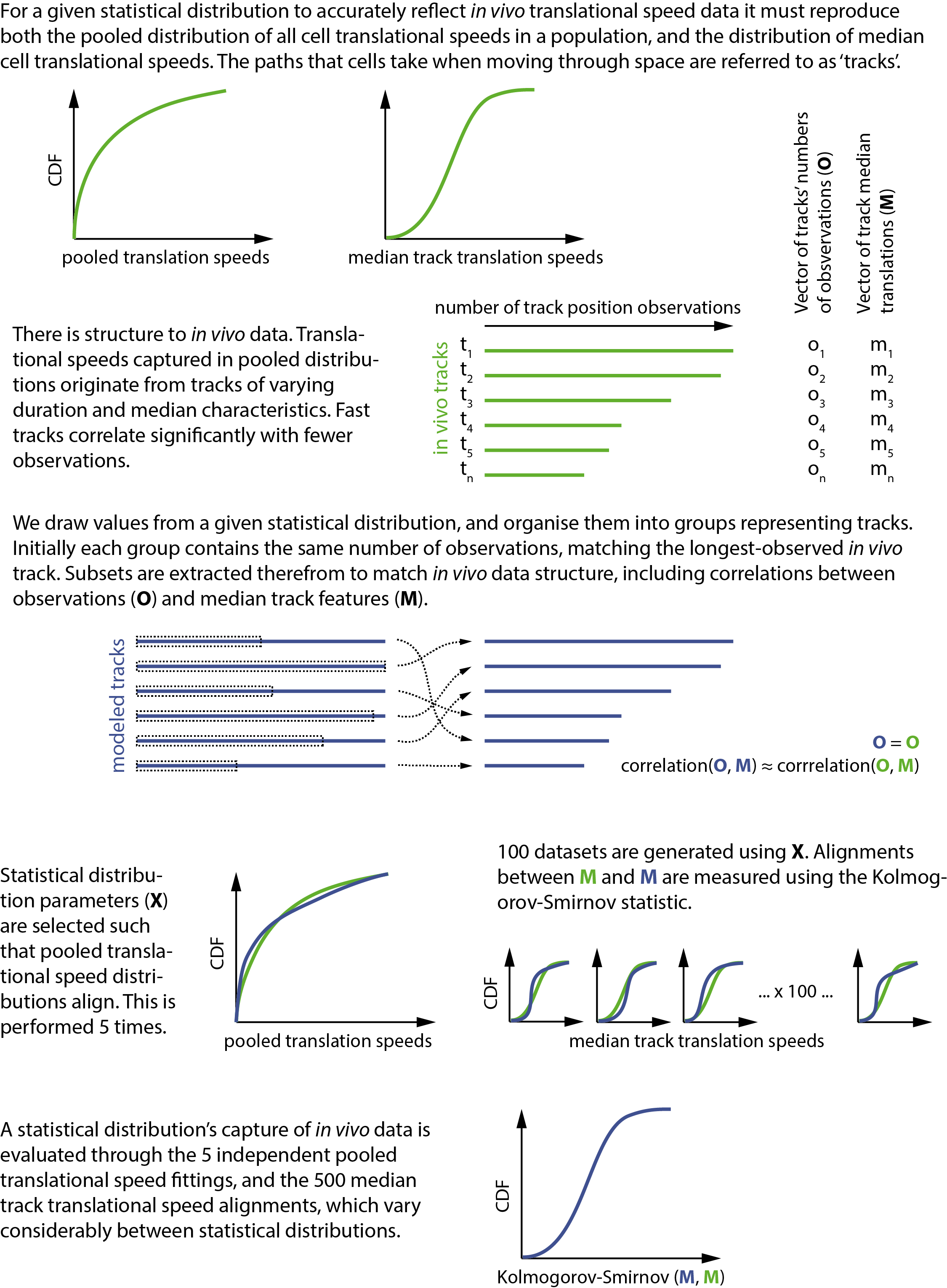

Supplement: S4 Fig — For brevity, the method is described as applying to translational speed data, however the same method is separately applied to turn speed data also. (PNG) [file pcbi.1005082.s005.png]

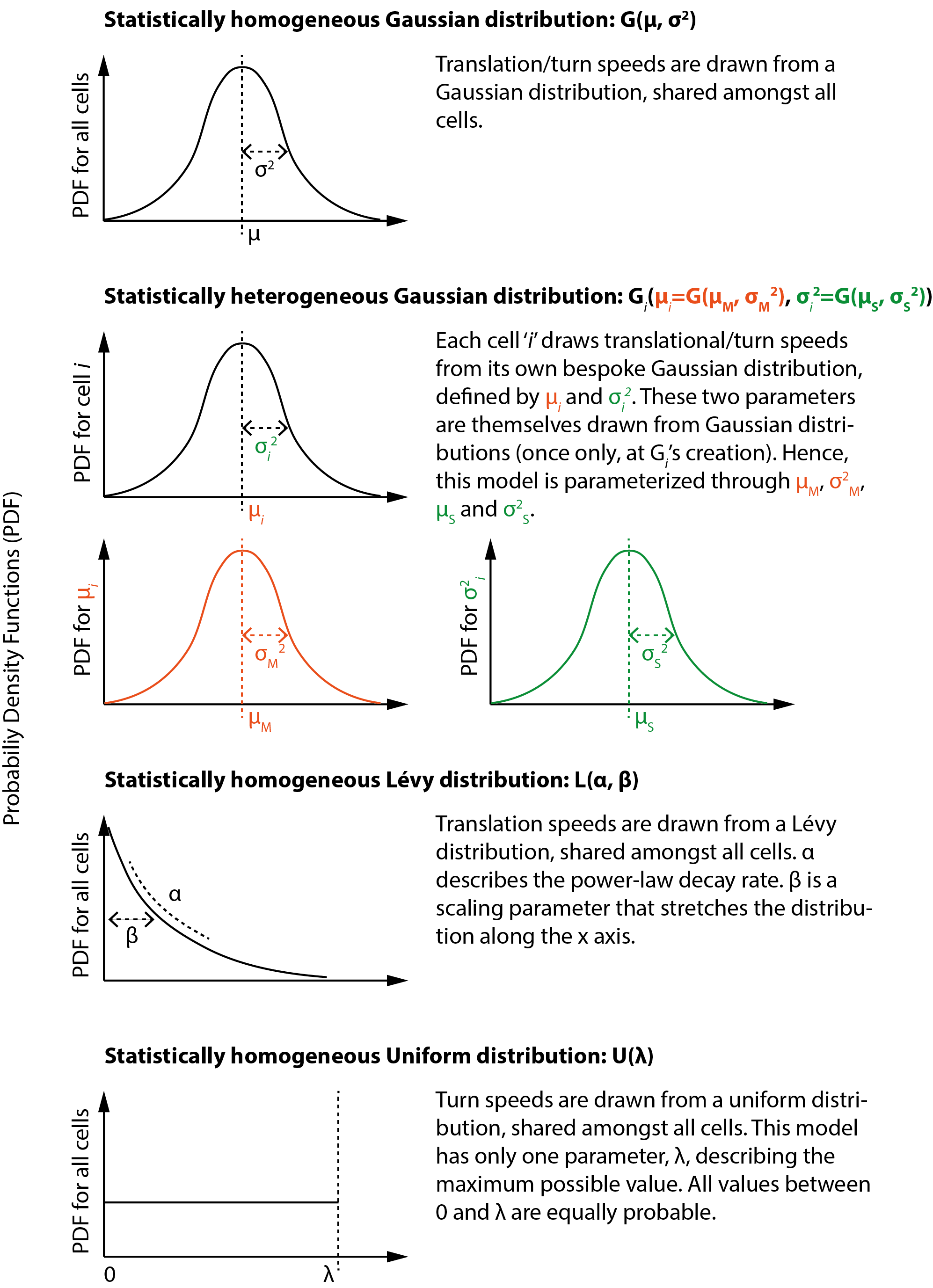

Supplement: S5 Fig — These distributions are fitted to in vivo cell translational and turn speed data to ascertain that cells are inherently heterogeneous in their motility characteristics. They are also used in designing random walk models subject to 3D agent-based simulation. (PNG) [file pcbi.1005082.s006.png]

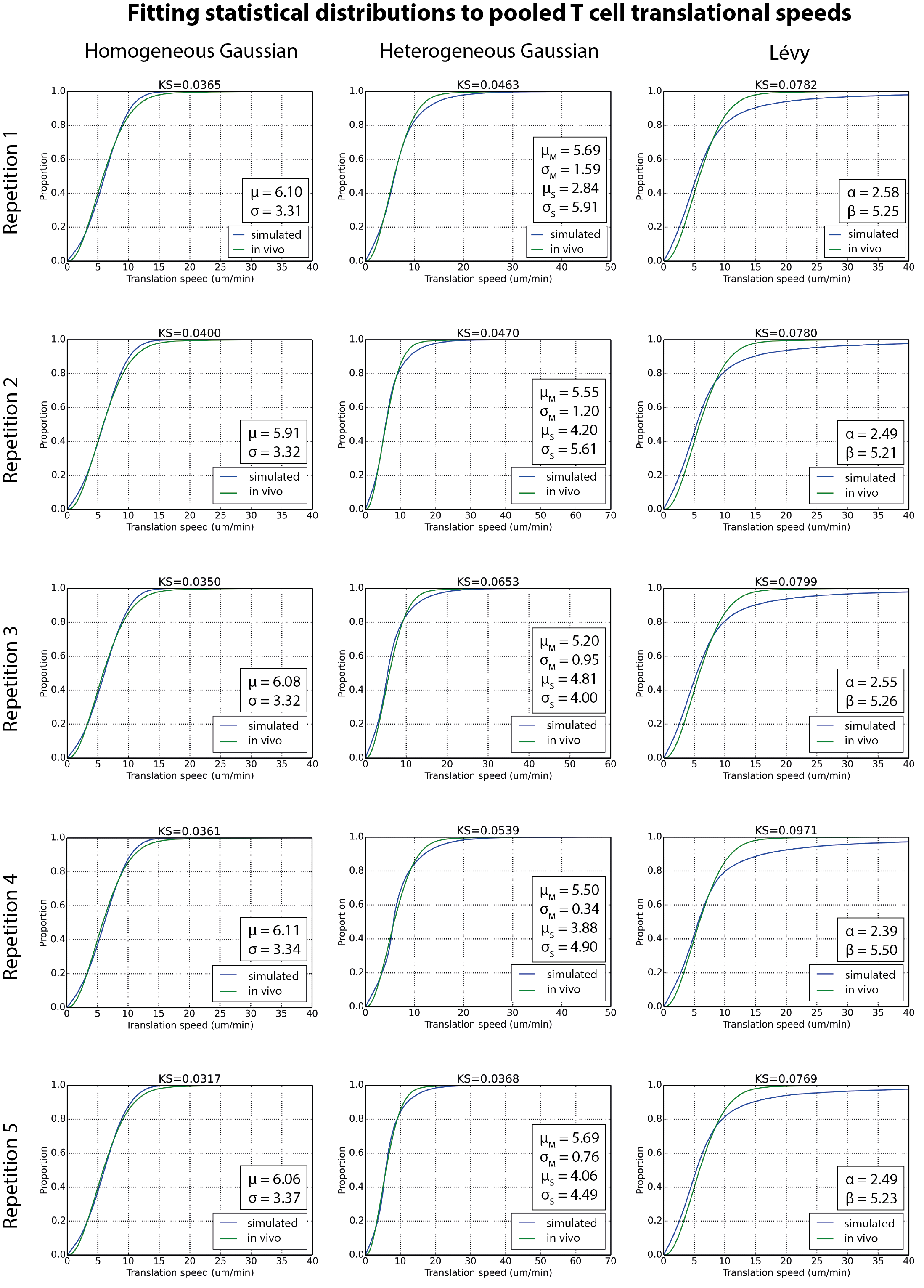

Supplement: S6 Fig — Each distribution was fitted to in vivo data five independent times, the resultant distribution parameters are given. (PNG) [file pcbi.1005082.s007.png]

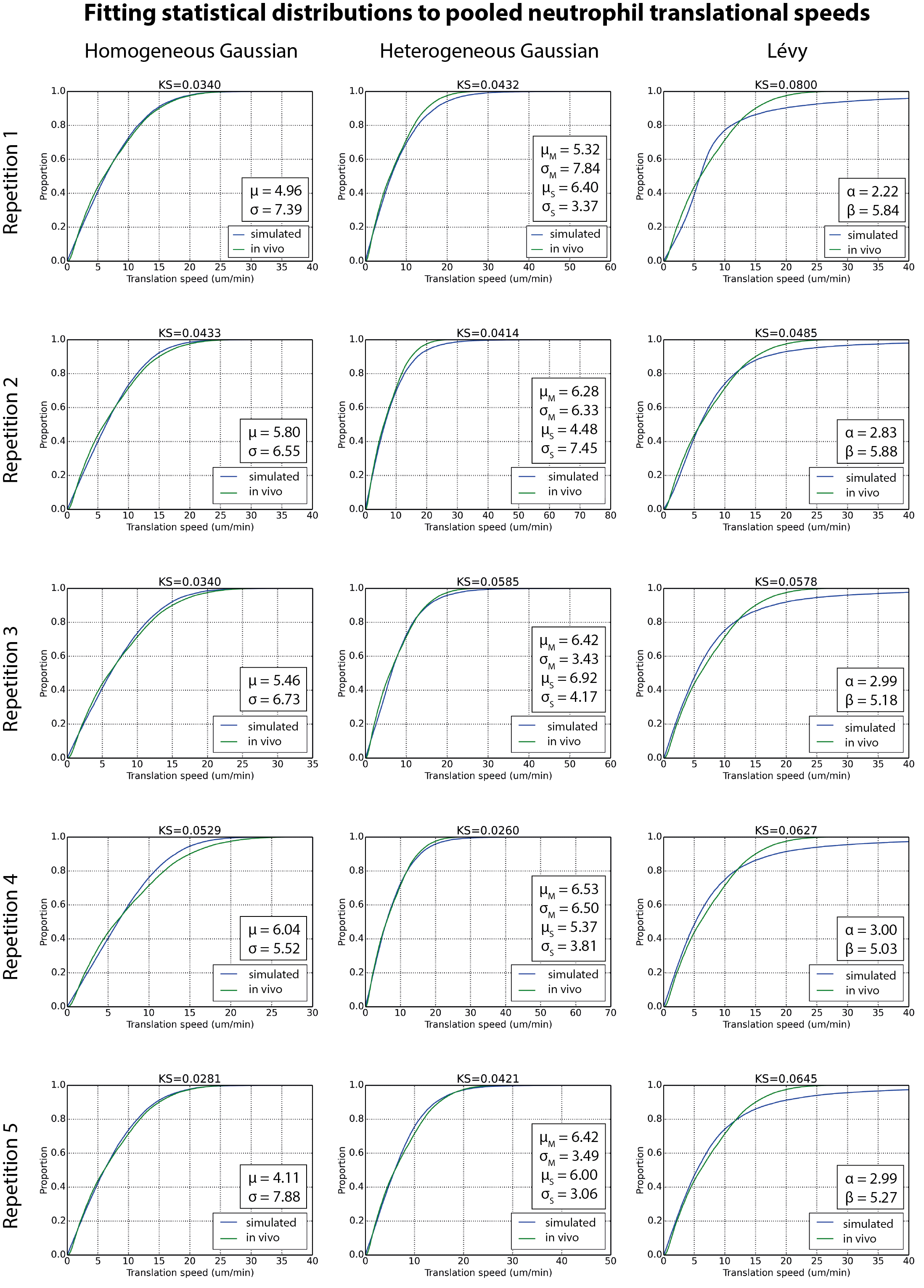

Supplement: S7 Fig — Each distribution was fitted to in vivo data five independent times, the resultant distribution parameters are given. (PNG) [file pcbi.1005082.s008.png]

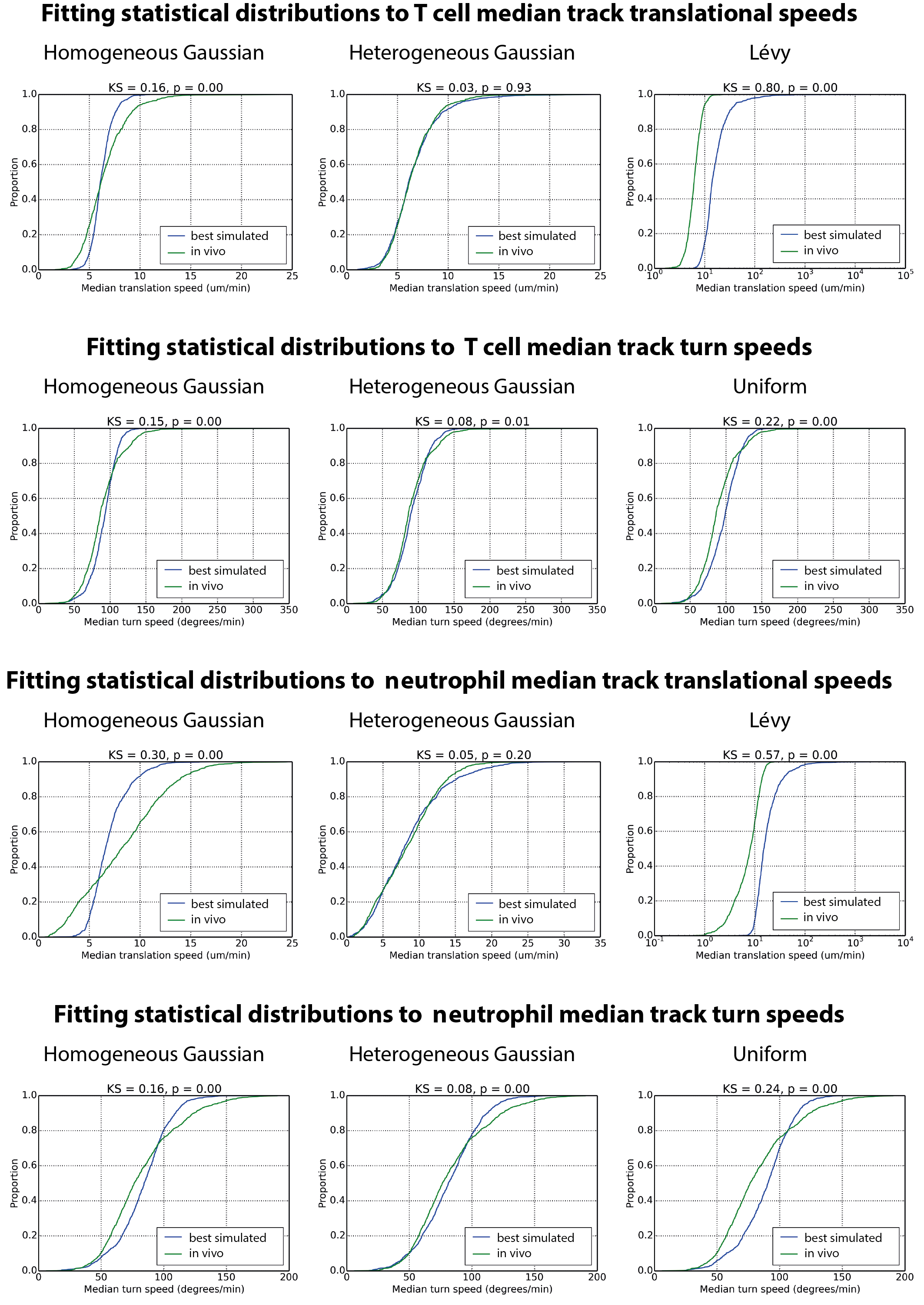

Supplement: S8 Fig — Each statistical distribution was fitted 5 independent times against pooled translational (or turn) speed distributions. Thereafter, each fitted distribution was used to generate 100 additional datasets using the method summarized in S4 Fig, constituting 500 for each model. Each of these 500 datasets’ median track characteristics were then contrasted with corresponding in vivo data. The best of those 500 alignments, as measured through the Kolmogorov-Smirnov statistic, are shown. (PNG) [file pcbi.1005082.s009.png]

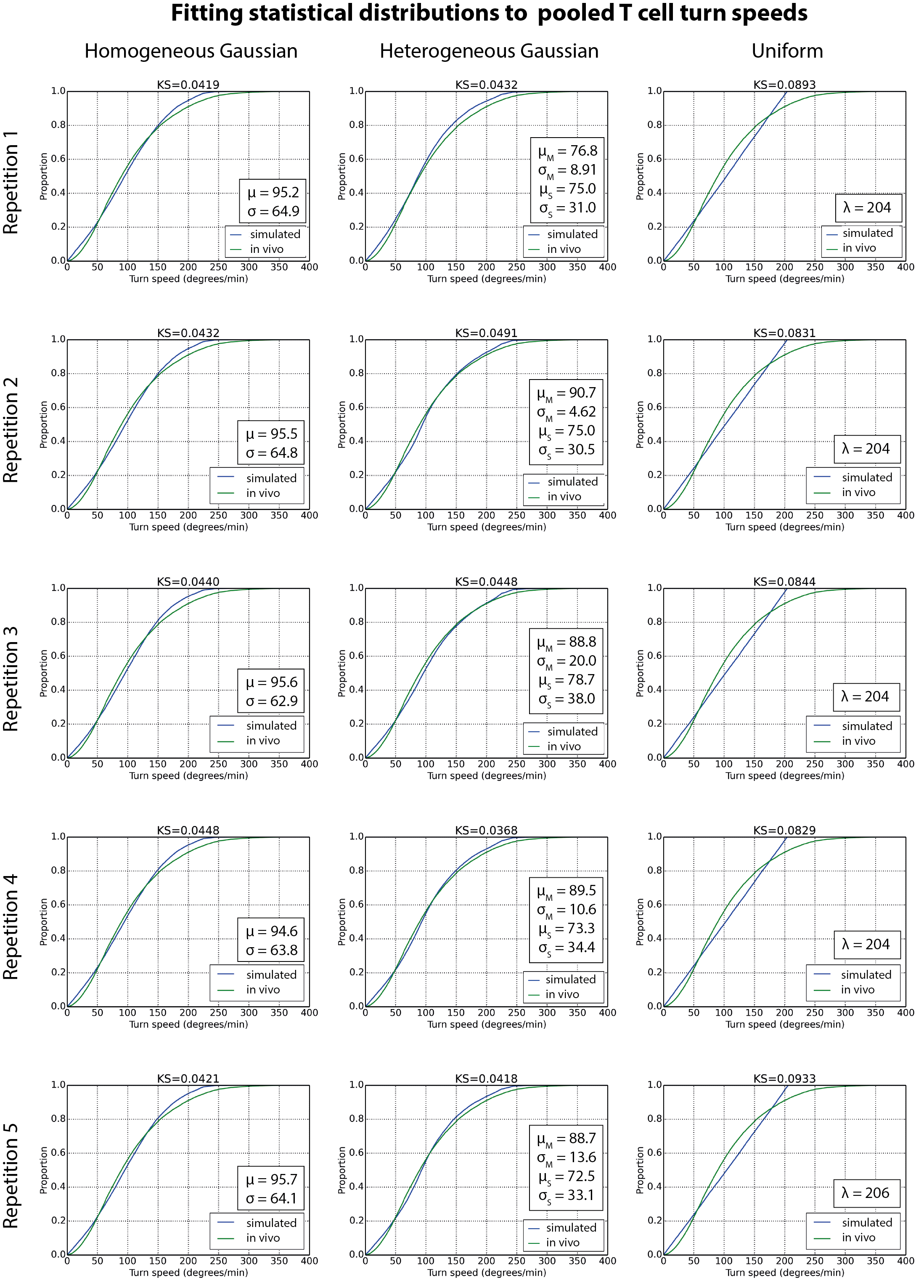

Supplement: S9 Fig — Each distribution was fitted to in vivo data five independent times, the resultant distribution parameters are given. (PNG) [file pcbi.1005082.s010.png]

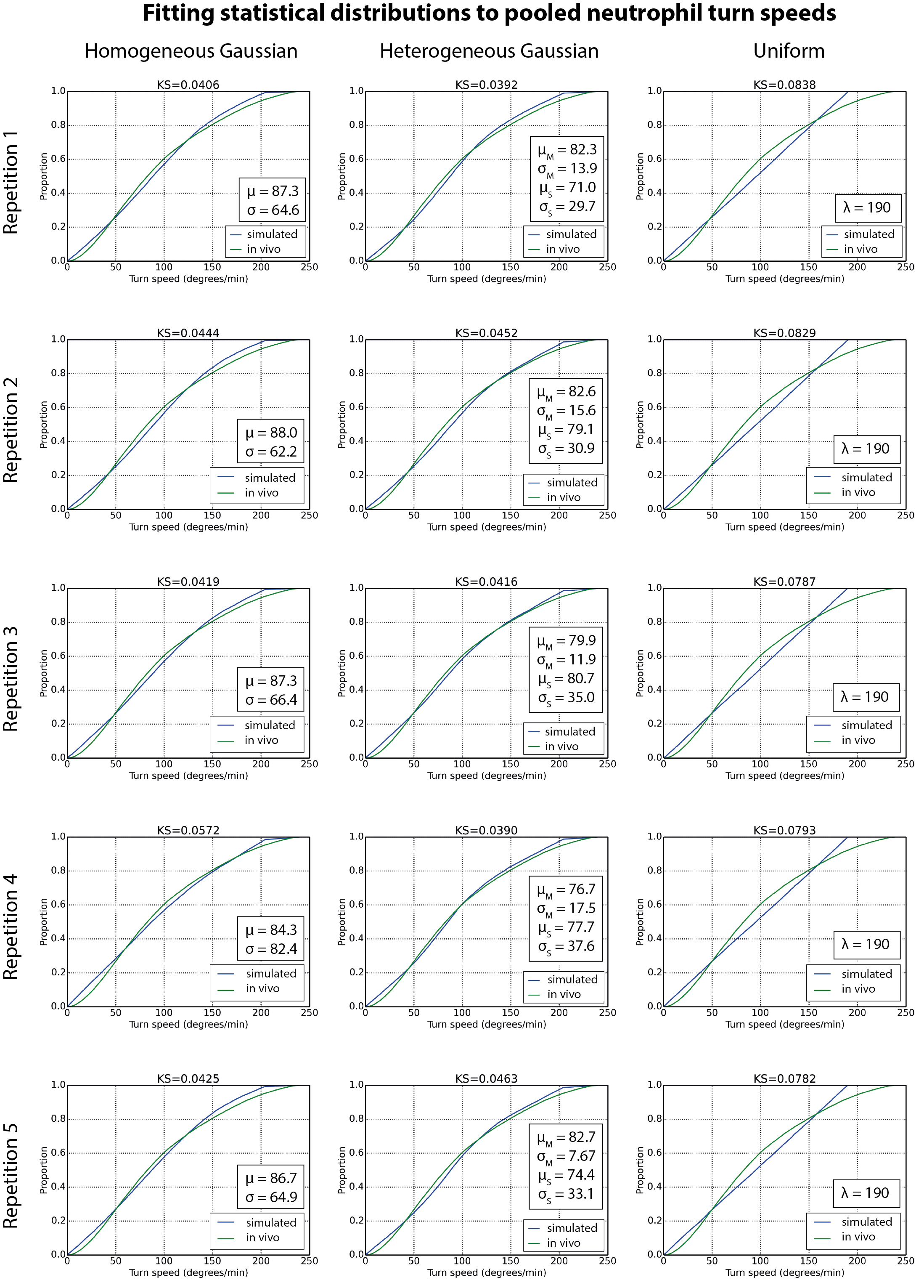

Supplement: S10 Fig — Each distribution was fitted to in vivo data five independent times, the resultant distribution parameters are given. (PNG) [file pcbi.1005082.s011.png]

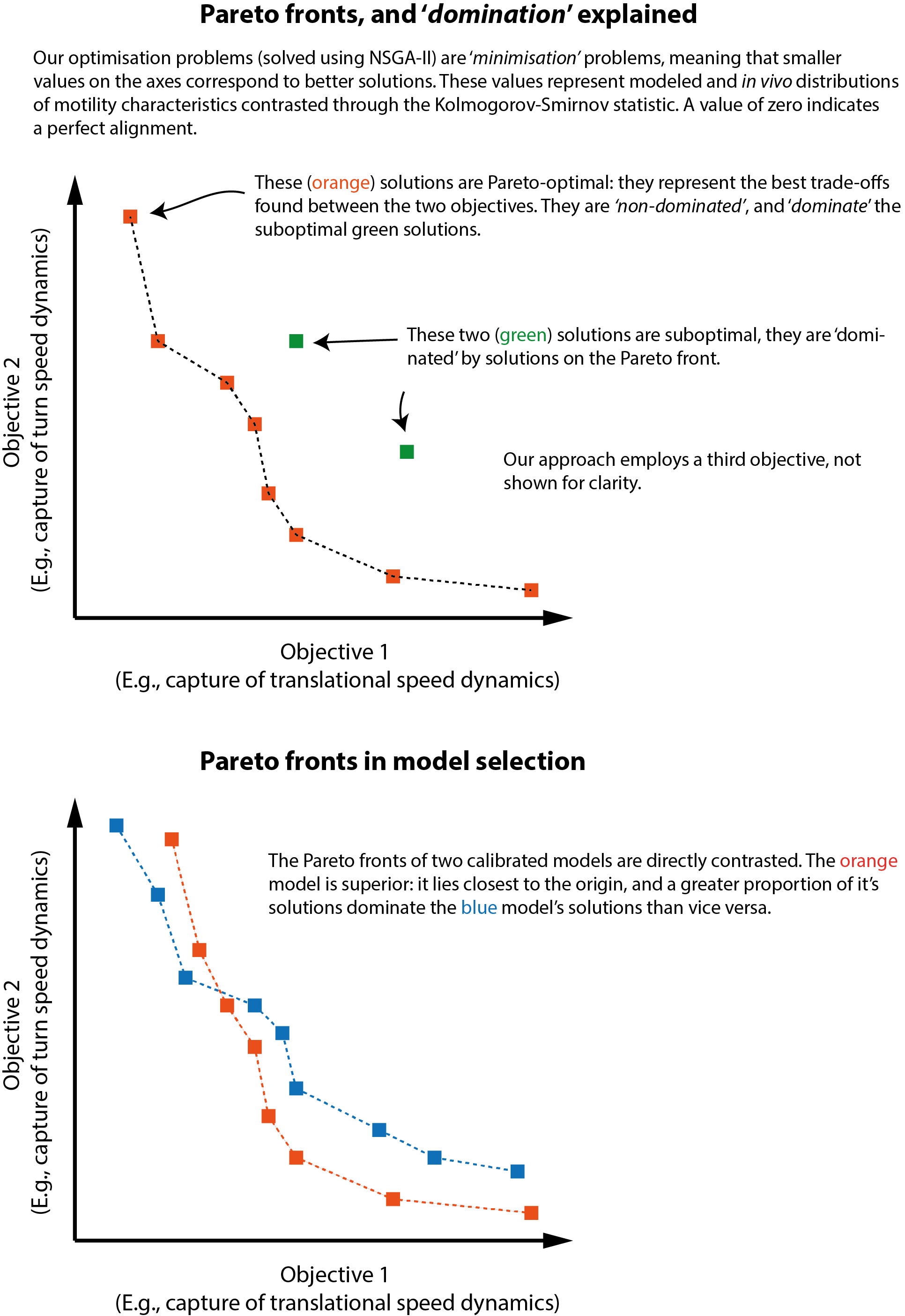

Supplement: S11 Fig — (PNG) [file pcbi.1005082.s012.png]

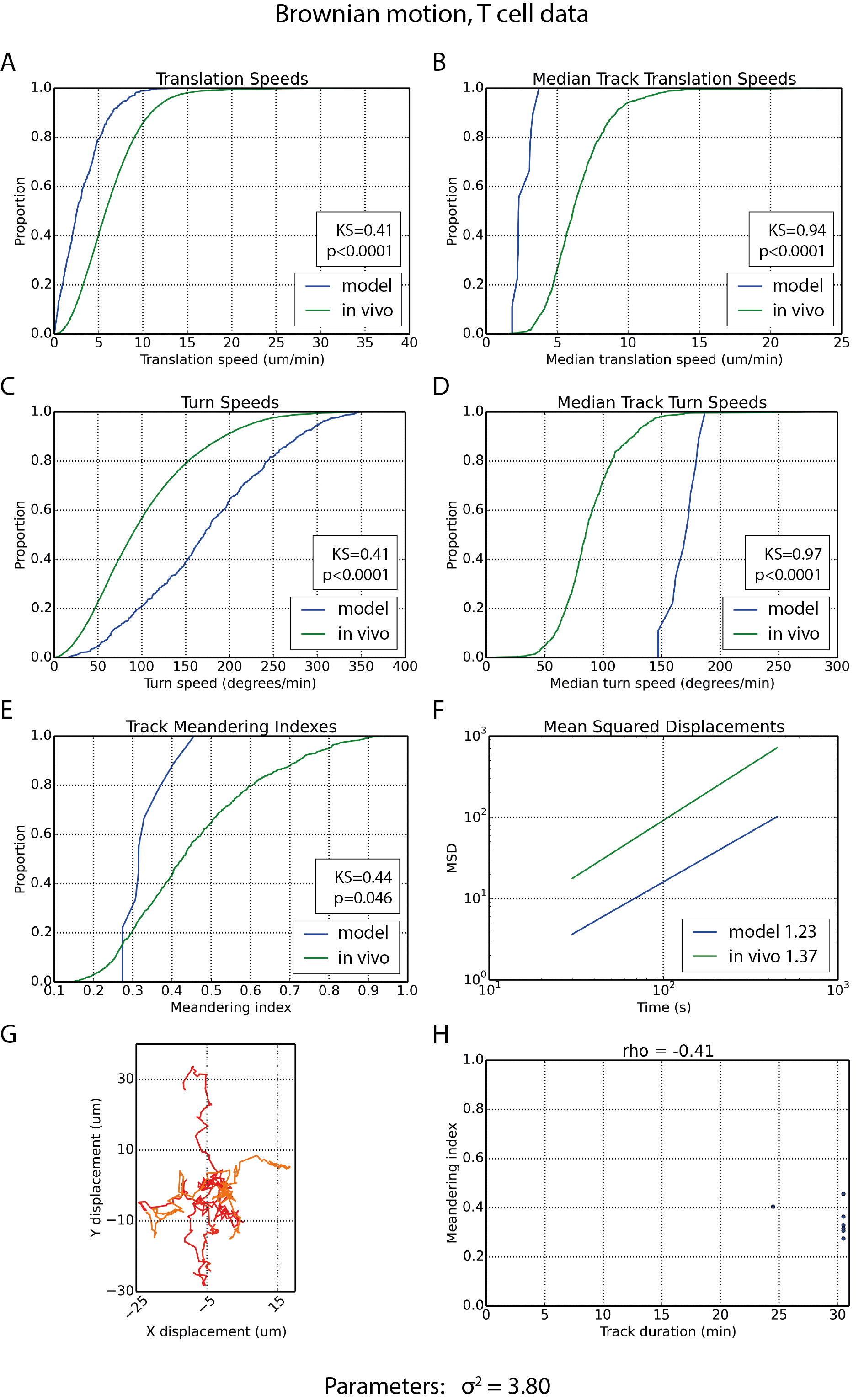

Supplement: S12 Fig — The best solution is that with the lowest Λ value. Pooled (A) and median track (B) translational speed distributions are shown as cumulative distribution plots. Similar plots, (C) and (D), depict turn speed data. (E) Cumulative distribution plot of track meandering index distributions. (F) Mean squared displacements for given durations (anywhere in the temporal domain, not from time zero only) plotted on log-log axes. The gradients of linear regression fitted models are given. (G) X and Y coordinates relative to starting positions of 7 tracks. (H) Scatter plot of track meandering indices against duration, Spearman’s rank correlation coefficient is given. The model’s parameter value is given. Brownian motion is a poor reflection of T cell motility dynamics: following removal of tracks <27μm net displacement, which is applied to in vivo data also, only 7 simulated tracks remain. We note that model calibration was performed using metrics of panels A, C and E only. (PNG) [file pcbi.1005082.s013.png]

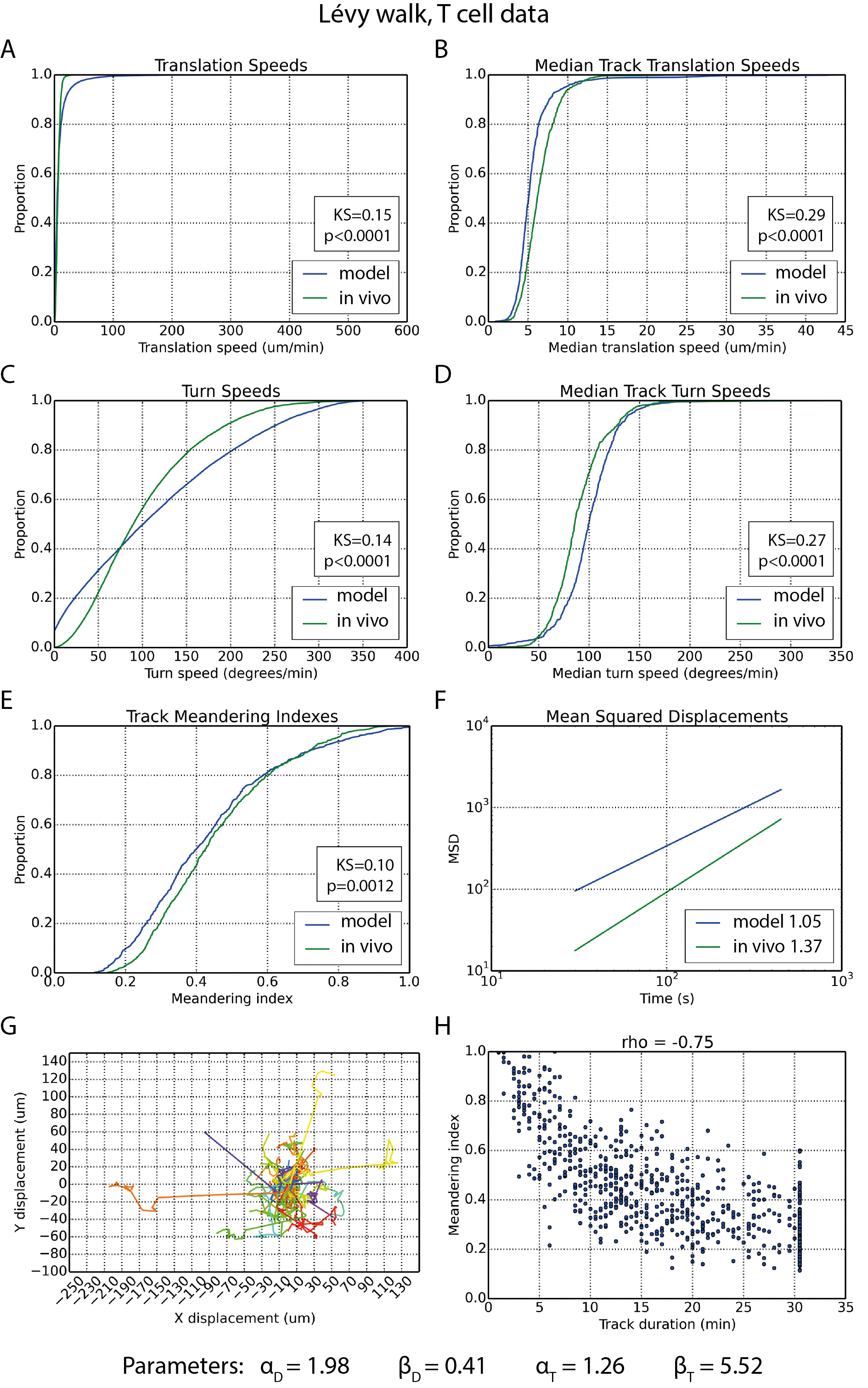

Supplement: S13 Fig — The best solution is that with the lowest Λ value. Pooled (A) and median track (B) translational speed distributions are shown as cumulative distribution plots. Similar plots, (C) and (D), depict turn speed data. (E) Cumulative distribution plot of track meandering index distributions. (F) Mean squared displacements for given durations (anywhere in the temporal domain, not from time zero only) plotted on log-log axes. The gradients of linear regression fitted models are given. (G) X and Y coordinates relative to starting positions of 40 tracks, selected to capture the entire range of net displacements. (H) Scatter plot of track meandering indices against duration, Spearman’s rank correlation coefficient is given. The model’s parameter values are given. We note that model calibration was performed using metrics of panels A, C and E only. (PNG) [file pcbi.1005082.s014.png]

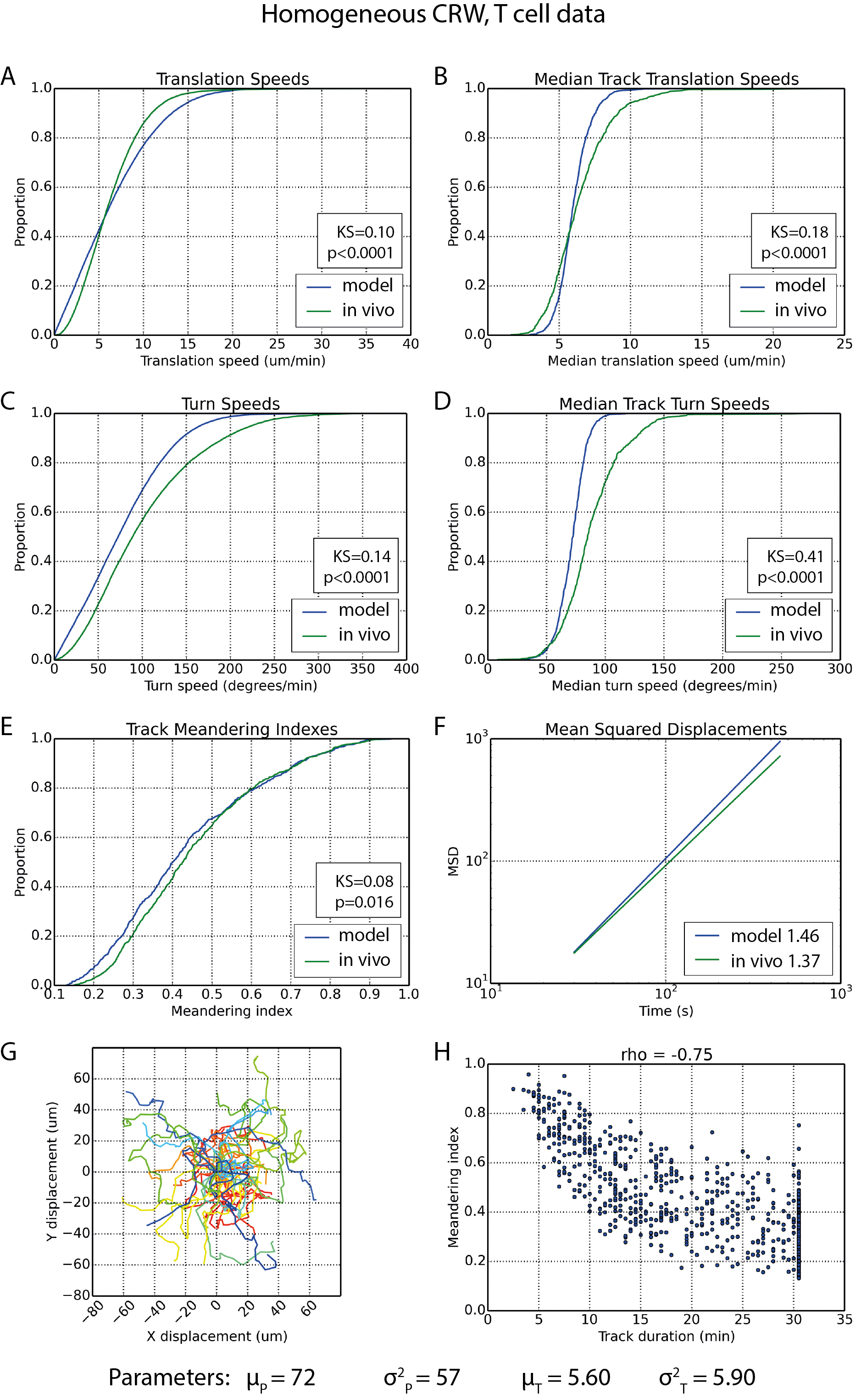

Supplement: S14 Fig — The best solution is that with the lowest Λ value. Pooled (A) and median track (B) translational speed distributions are shown as cumulative distribution plots. Similar plots, (C) and (D), depict turn speed data. (E) Cumulative distribution plot of track meandering index distributions. (F) Mean squared displacements for given durations (anywhere in the temporal domain, not from time zero only) plotted on log-log axes. The gradients of linear regression fitted models are given. (G) X and Y coordinates relative to starting positions of 40 tracks, selected to capture the entire range of net displacements. (H) Scatter plot of track meandering indices against duration, Spearman’s rank correlation coefficient is given. The model’s parameter values are given. We note that model calibration was performed using metrics of panels A, C and E only. (PNG) [file pcbi.1005082.s015.png]

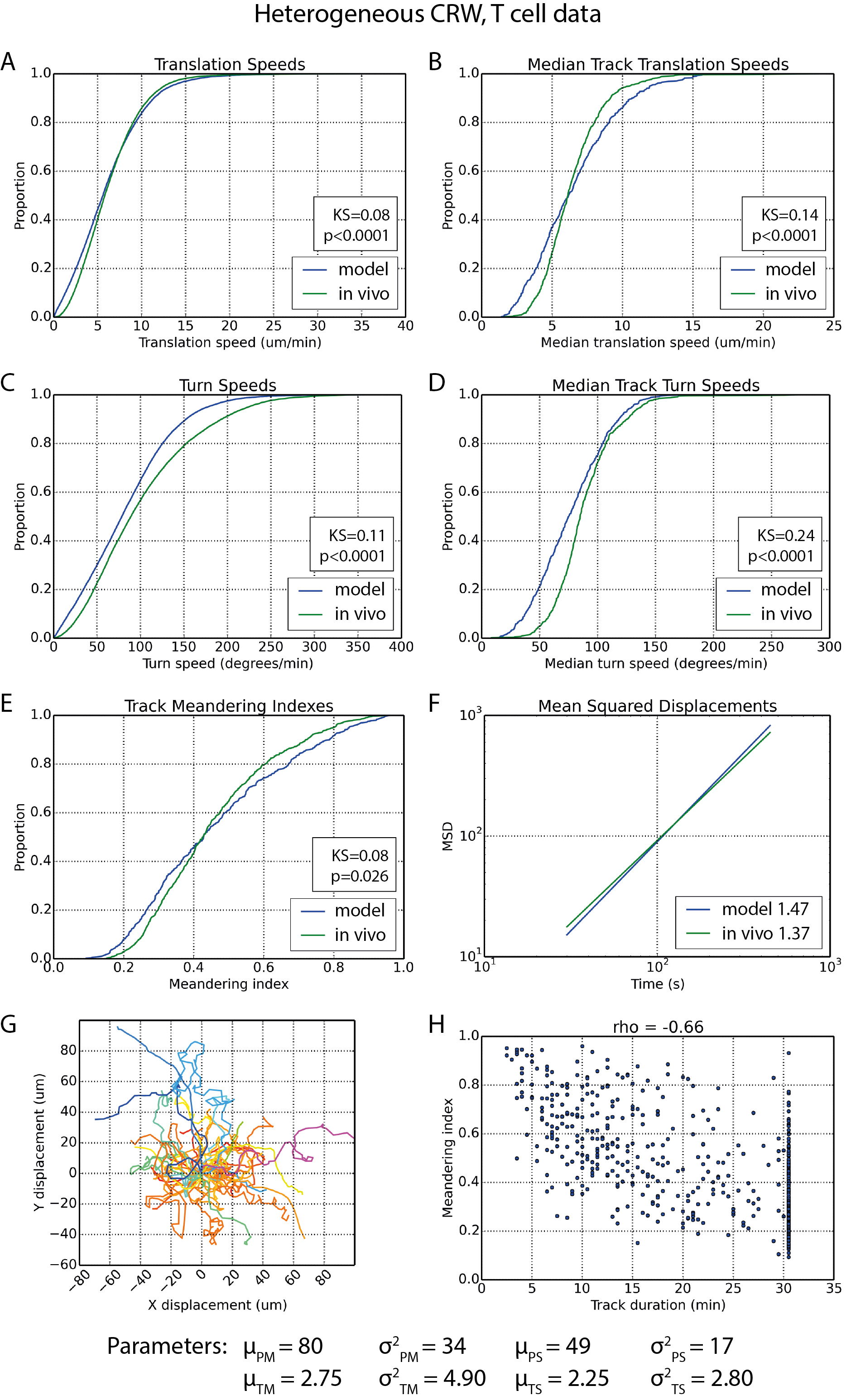

Supplement: S15 Fig — The best solution is that with the lowest Λ value. Pooled (A) and median track (B) translational speed distributions are shown as cumulative distribution plots. Similar plots, (C) and (D), depict turn speed data. (E) Cumulative distribution plot of track meandering index distributions. (F) Mean squared displacements for given durations (anywhere in the temporal domain, not from time zero only) plotted on log-log axes. The gradients of linear regression fitted models are given. (G) X and Y coordinates relative to starting positions of 40 tracks, selected to capture the entire range of net displacements. (H) Scatter plot of track meandering indices against duration, Spearman’s rank correlation coefficient is given. The model’s parameter values are given. We note that model calibration was performed using metrics of panels A, C and E only. (PNG) [file pcbi.1005082.s016.png]

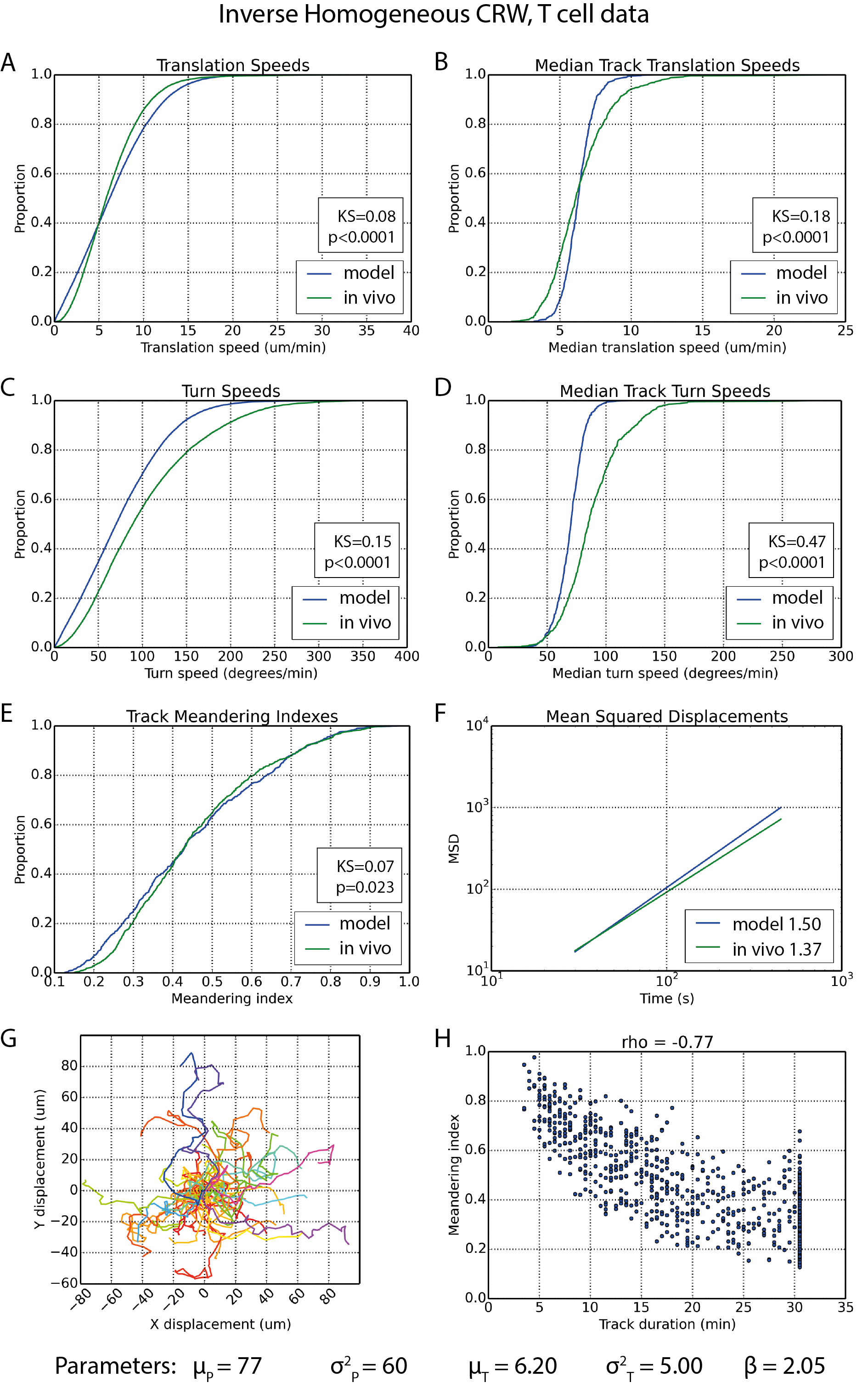

Supplement: S16 Fig — The best solution is that with the lowest Λ value. Pooled (A) and median track (B) translational speed distributions are shown as cumulative distribution plots. Similar plots, (C) and (D), depict turn speed data. (E) Cumulative distribution plot of track meandering index distributions. (F) Mean squared displacements for given durations (anywhere in the temporal domain, not from time zero only) plotted on log-log axes. The gradients of linear regression fitted models are given. (G) X and Y coordinates relative to starting positions of 40 tracks, selected to capture the entire range of net displacements. (H) Scatter plot of track meandering indices against duration, Spearman’s rank correlation coefficient is given. The model’s parameter values are given. We note that model calibration was performed using metrics of panels A, C and E only. (PNG) [file pcbi.1005082.s017.png]

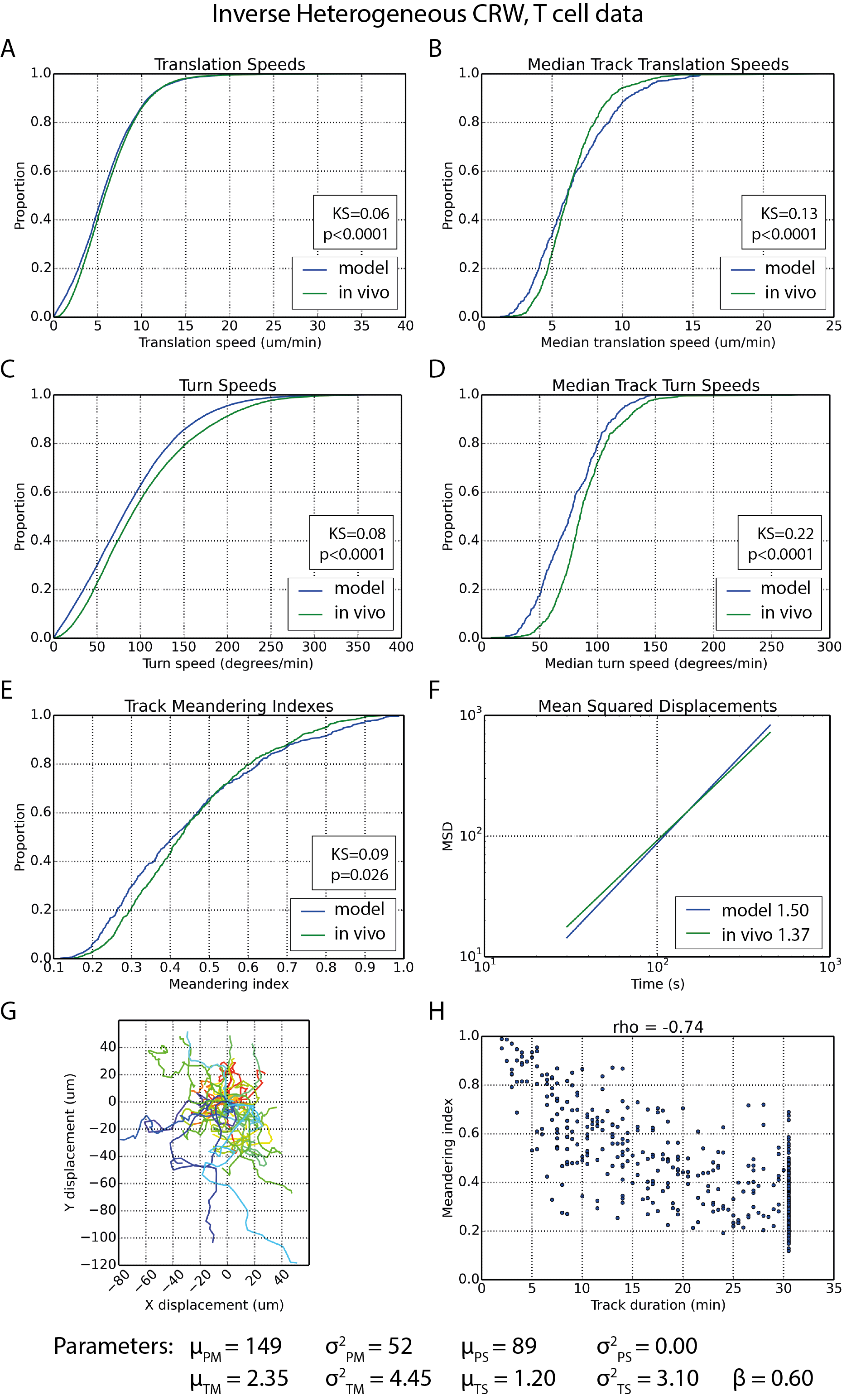

Supplement: S17 Fig — The best solution is that with the lowest Λ value. Pooled (A) and median track (B) translational speed distributions are shown as cumulative distribution plots. Similar plots, (C) and (D), depict turn speed data. (E) Cumulative distribution plot of track meandering index distributions. (F) Mean squared displacements for given durations (anywhere in the temporal domain, not from time zero only) plotted on log-log axes. The gradients of linear regression fitted models are given. (G) X and Y coordinates relative to starting positions of 40 tracks, selected to capture the entire range of net displacements. (H) Scatter plot of track meandering indices against duration, Spearman’s rank correlation coefficient is given. The model’s parameter values are given. We note that model calibration was performed using metrics of panels A, C and E only. (PNG) [file pcbi.1005082.s018.png]

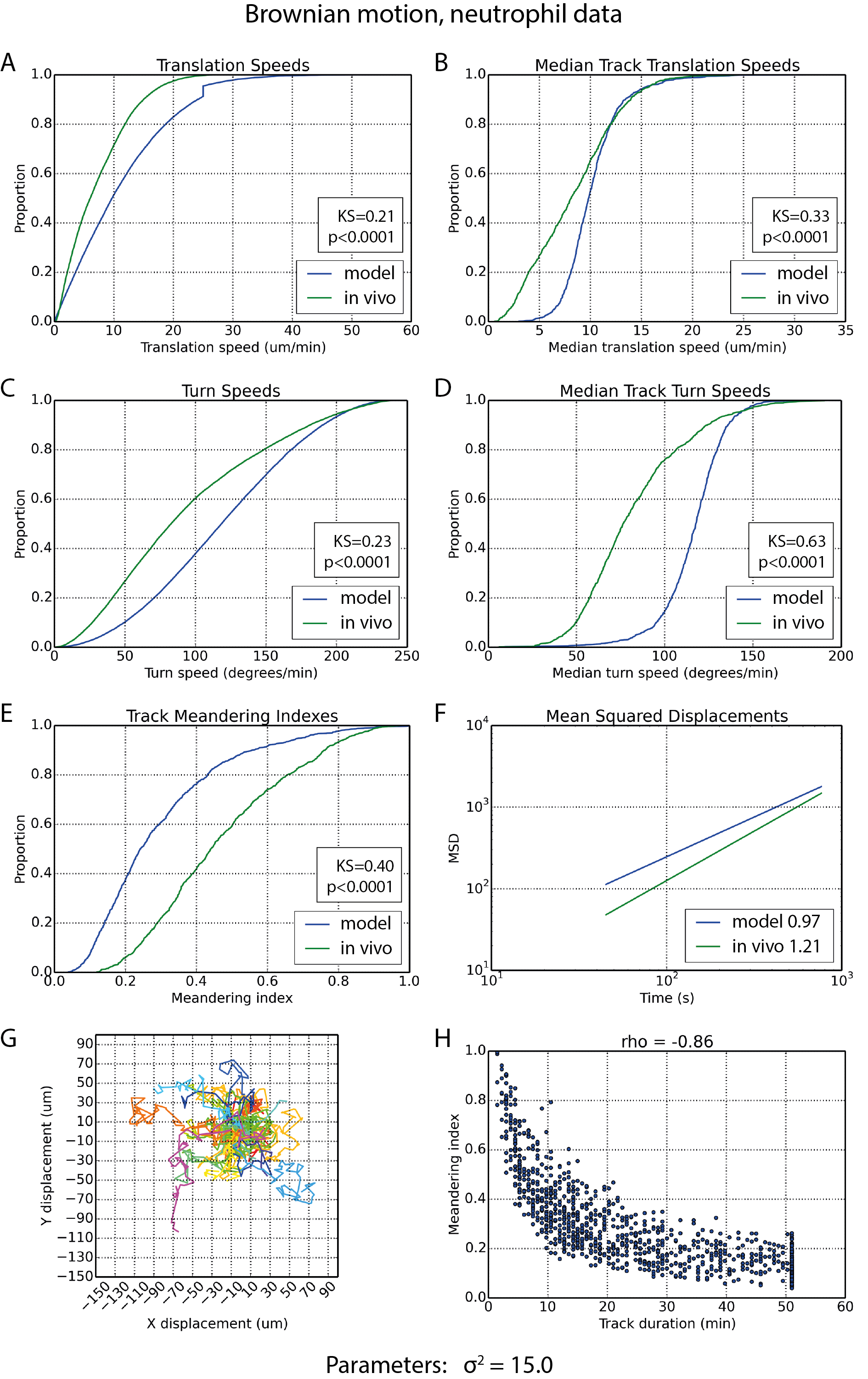

Supplement: S18 Fig — The best solution is that with the lowest Λ value. Pooled (A) and median track (B) translational speed distributions are shown as cumulative distribution plots. Similar plots, (C) and (D), depict turn speed data. (E) Cumulative distribution plot of track meandering index distributions. (F) Mean squared displacements for given durations (anywhere in the temporal domain, not from time zero only) plotted on log-log axes. The gradients of linear regression fitted models are given. (G) X and Y coordinates relative to starting positions of 40 tracks, selected to capture the entire range of net displacements. (H) Scatter plot of track meandering indices against duration, Spearman’s rank correlation coefficient is given. The model’s parameter values are given. We note that model calibration was performed using metrics of panels A, C and E only. (PNG) [file pcbi.1005082.s019.png]

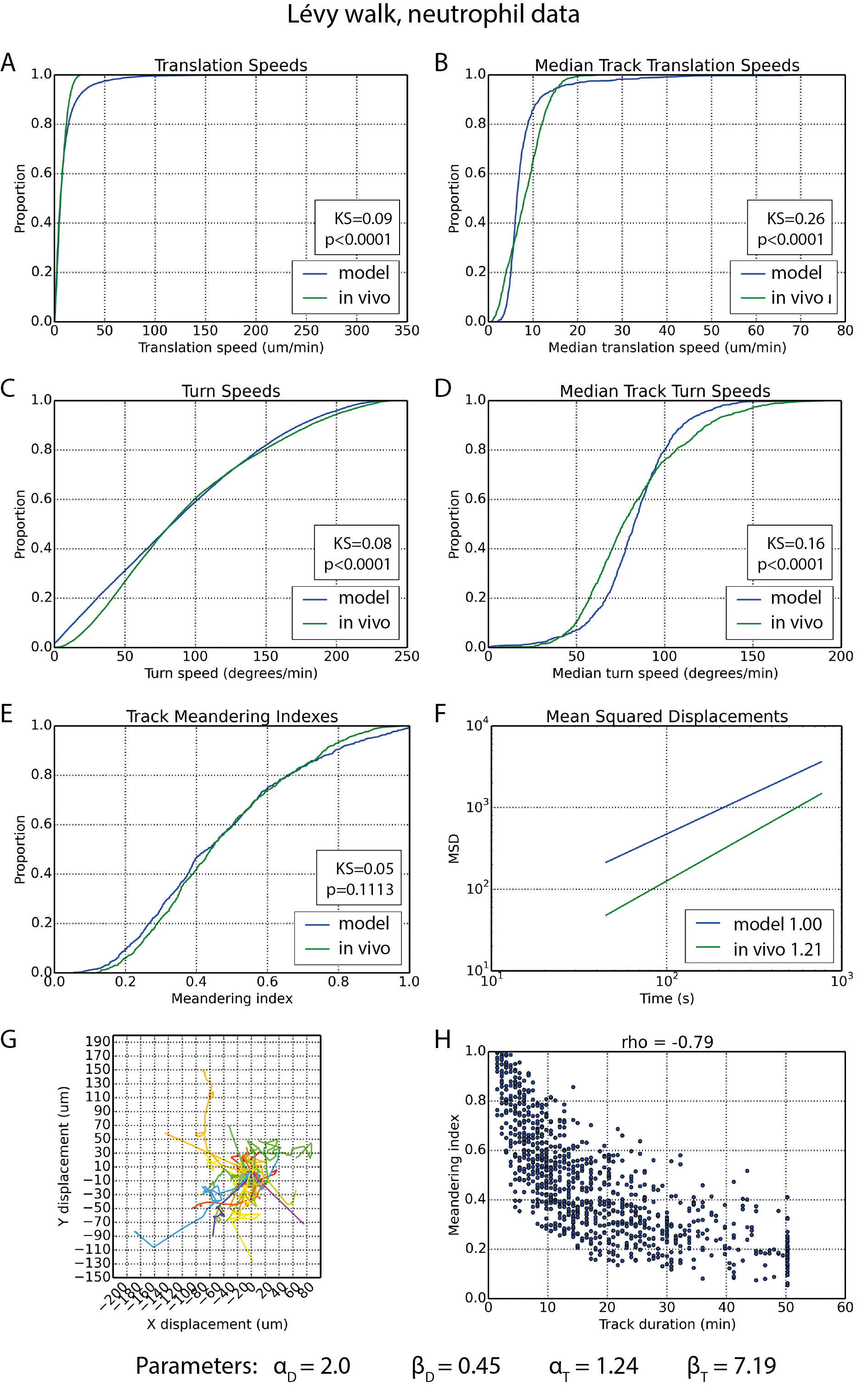

Supplement: S19 Fig — The best solution is that with the lowest Λ value. Pooled (A) and median track (B) translational speed distributions are shown as cumulative distribution plots. Similar plots, (C) and (D), depict turn speed data. (E) Cumulative distribution plot of track meandering index distributions. (F) Mean squared displacements for given durations (anywhere in the temporal domain, not from time zero only) plotted on log-log axes. The gradients of linear regression fitted models are given. (G) X and Y coordinates relative to starting positions of 40 tracks, selected to capture the entire range of net displacements. (H) Scatter plot of track meandering indices against duration, Spearman’s rank correlation coefficient is given. The model’s parameter values are given. We note that model calibration was performed using metrics of panels A, C and E only. (PNG) [file pcbi.1005082.s020.png]

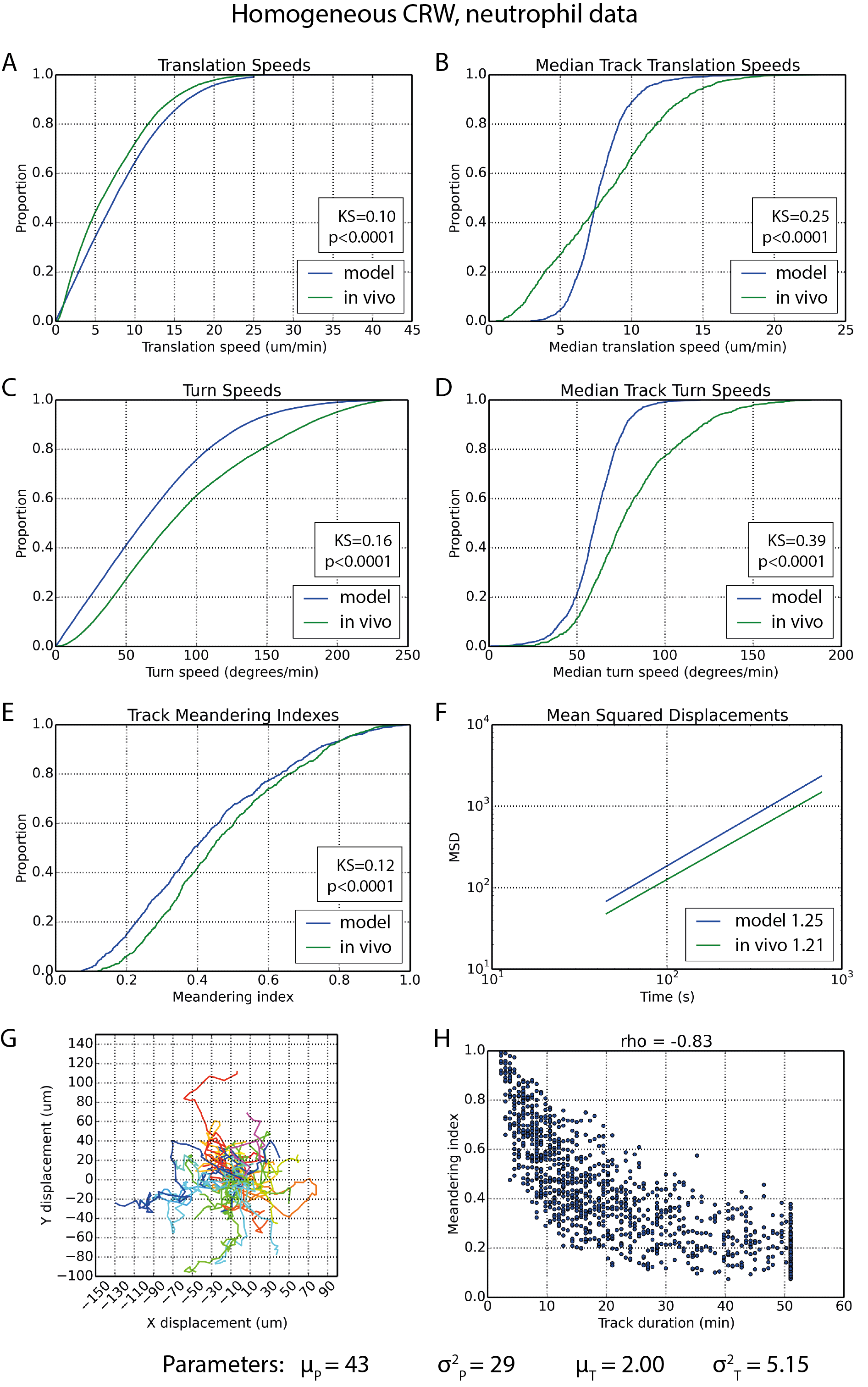

Supplement: S20 Fig — The best solution is that with the lowest Λ value. Pooled (A) and median track (B) translational speed distributions are shown as cumulative distribution plots. Similar plots, (C) and (D), depict turn speed data. (E) Cumulative distribution plot of track meandering index distributions. (F) Mean squared displacements for given durations (anywhere in the temporal domain, not from time zero only) plotted on log-log axes. The gradients of linear regression fitted models are given. (G) X and Y coordinates relative to starting positions of 40 tracks, selected to capture the entire range of net displacements. (H) Scatter plot of track meandering indices against duration, Spearman’s rank correlation coefficient is given. The model’s parameter values are given. We note that model calibration was performed using metrics of panels A, C and E only. (PNG) [file pcbi.1005082.s021.png]

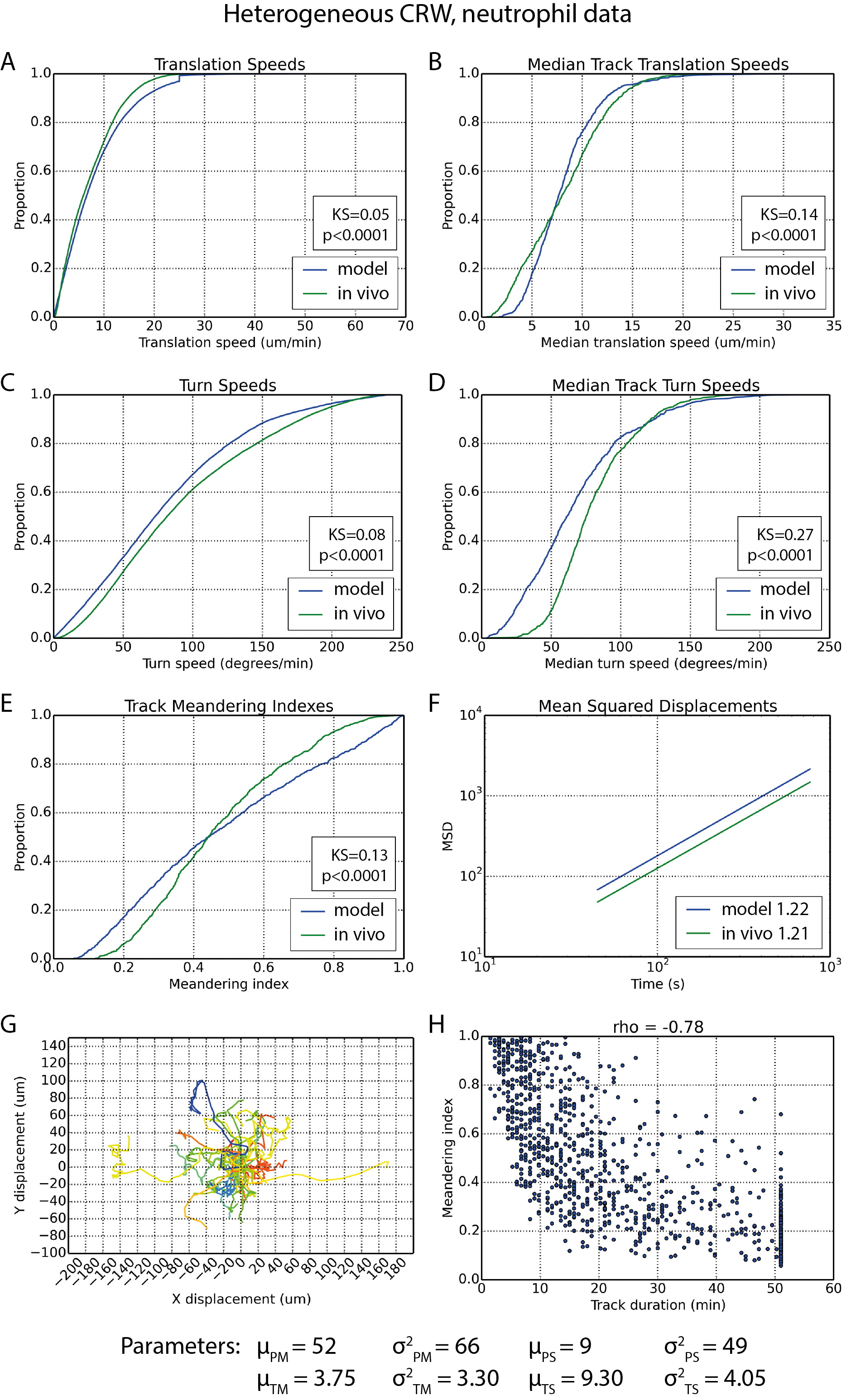

Supplement: S21 Fig — The best solution is that with the lowest Λ value. Pooled (A) and median track (B) translational speed distributions are shown as cumulative distribution plots. Similar plots, (C) and (D), depict turn speed data. (E) Cumulative distribution plot of track meandering index distributions. (F) Mean squared displacements for given durations (anywhere in the temporal domain, not from time zero only) plotted on log-log axes. The gradients of linear regression fitted models are given. (G) X and Y coordinates relative to starting positions of 40 tracks, selected to capture the entire range of net displacements. (H) Scatter plot of track meandering indices against duration, Spearman’s rank correlation coefficient is given. The model’s parameter values are given. We note that model calibration was performed using metrics of panels A, C and E only. (PNG) [file pcbi.1005082.s022.png]

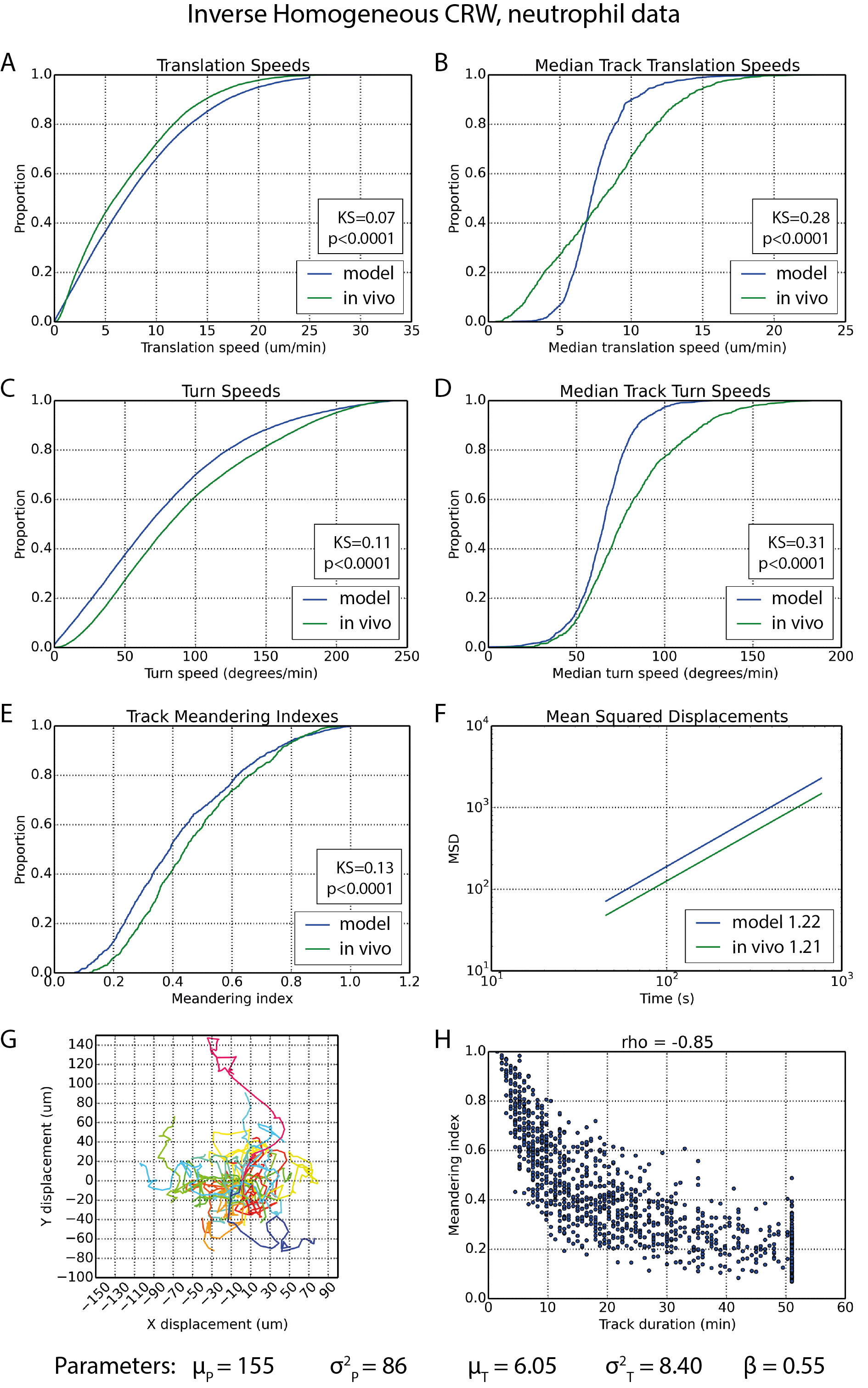

Supplement: S22 Fig — The best solution is that with the lowest Λ value. Pooled (A) and median track (B) translational speed distributions are shown as cumulative distribution plots. Similar plots, (C) and (D), depict turn speed data. (E) Cumulative distribution plot of track meandering index distributions. (F) Mean squared displacements for given durations (anywhere in the temporal domain, not from time zero only) plotted on log-log axes. The gradients of linear regression fitted models are given. (G) X and Y coordinates relative to starting positions of 40 tracks, selected to capture the entire range of net displacements. (H) Scatter plot of track meandering indices against duration, Spearman’s rank correlation coefficient is given. The model’s parameter values are given. We note that model calibration was performed using metrics of panels A, C and E only. (PNG) [file pcbi.1005082.s023.png]

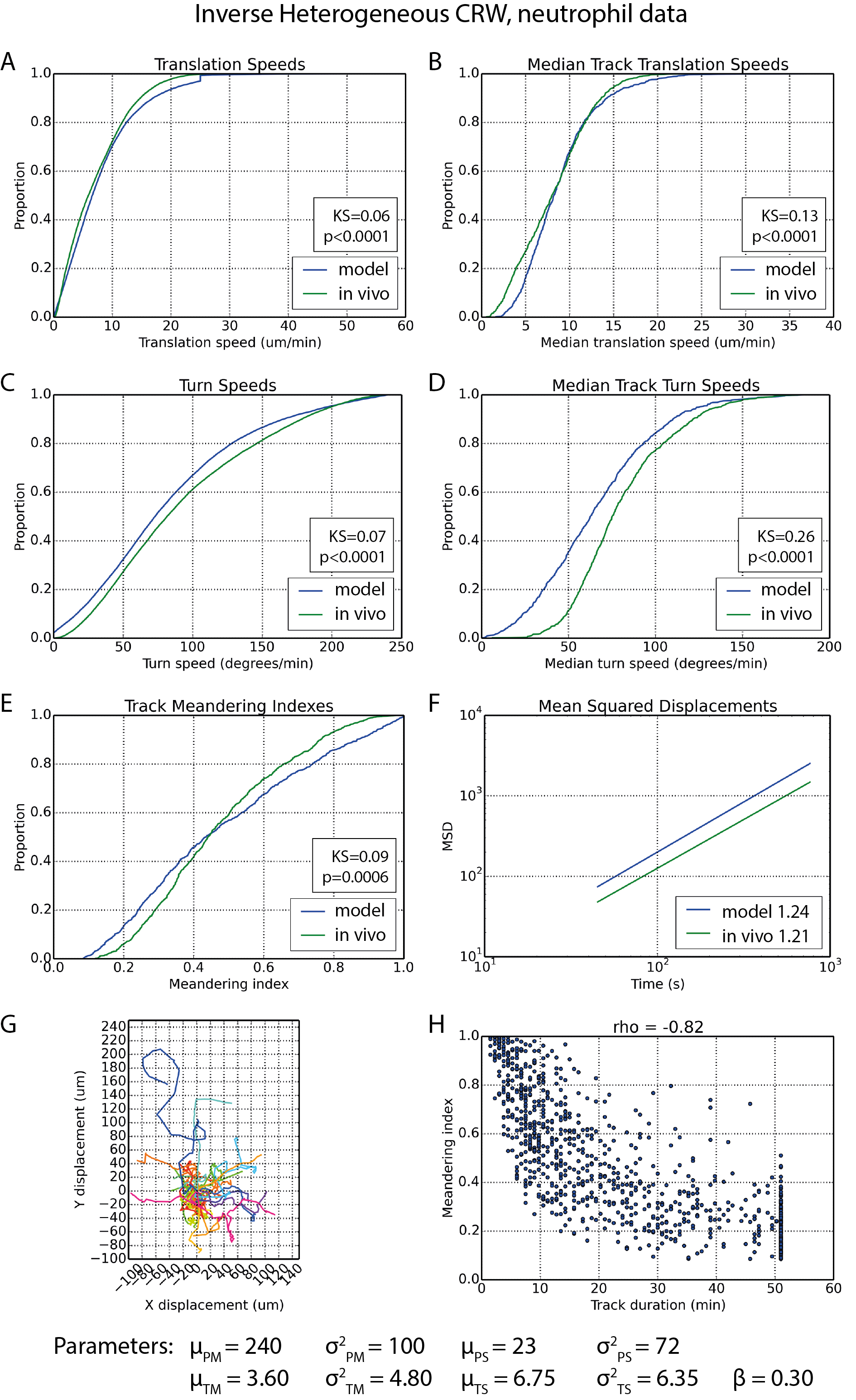

Supplement: S23 Fig — The best solution is that with the lowest Λ value. Pooled (A) and median track (B) translational speed distributions are shown as cumulative distribution plots. Similar plots, (C) and (D), depict turn speed data. (E) Cumulative distribution plot of track meandering index distributions. (F) Mean squared displacements for given durations (anywhere in the temporal domain, not from time zero only) plotted on log-log axes. The gradients of linear regression fitted models are given. (G) X and Y coordinates relative to starting positions of 40 tracks, selected to capture the entire range of net displacements. (H) Scatter plot of track meandering indices against duration, Spearman’s rank correlation coefficient is given. The model’s parameter values are given. We note that model calibration was performed using metrics of panels A, C and E only. (PNG) [file pcbi.1005082.s024.png]

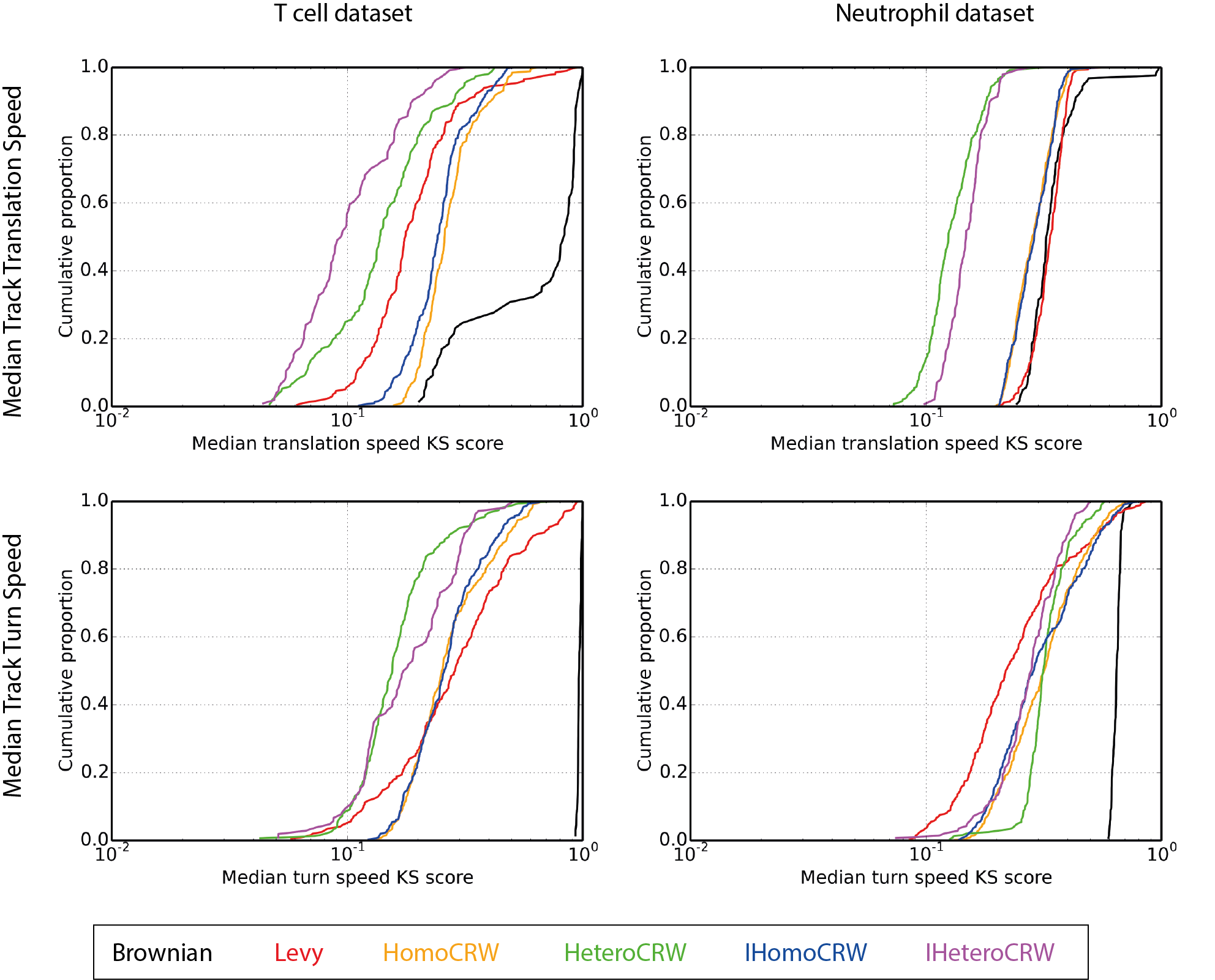

Supplement: S24 Fig — Each motility model was independently simulated and calibrated against both T cell and neutrophil data 3 times. Pareto fronts were compiled for each model from the solutions in all three calibrations. The alignment of each Pareto front solution median track translation/turn distribution with the corresponding in vivo data was assessed using the Kolmogorov-Smirnov (KS) statistic. Shown here are the distributions of KS values across all Pareto front solutions. These data broadly correspond with the independent statistical modeling of translation and turn speed dynamics, Fig 1. Heterogeneous CRW models better capture T cell and neutrophil dynamics than homogeneous CRW models, with the exception of neutrophil median track turn speeds, where no discernible difference is found. (PNG) [file pcbi.1005082.s025.png]

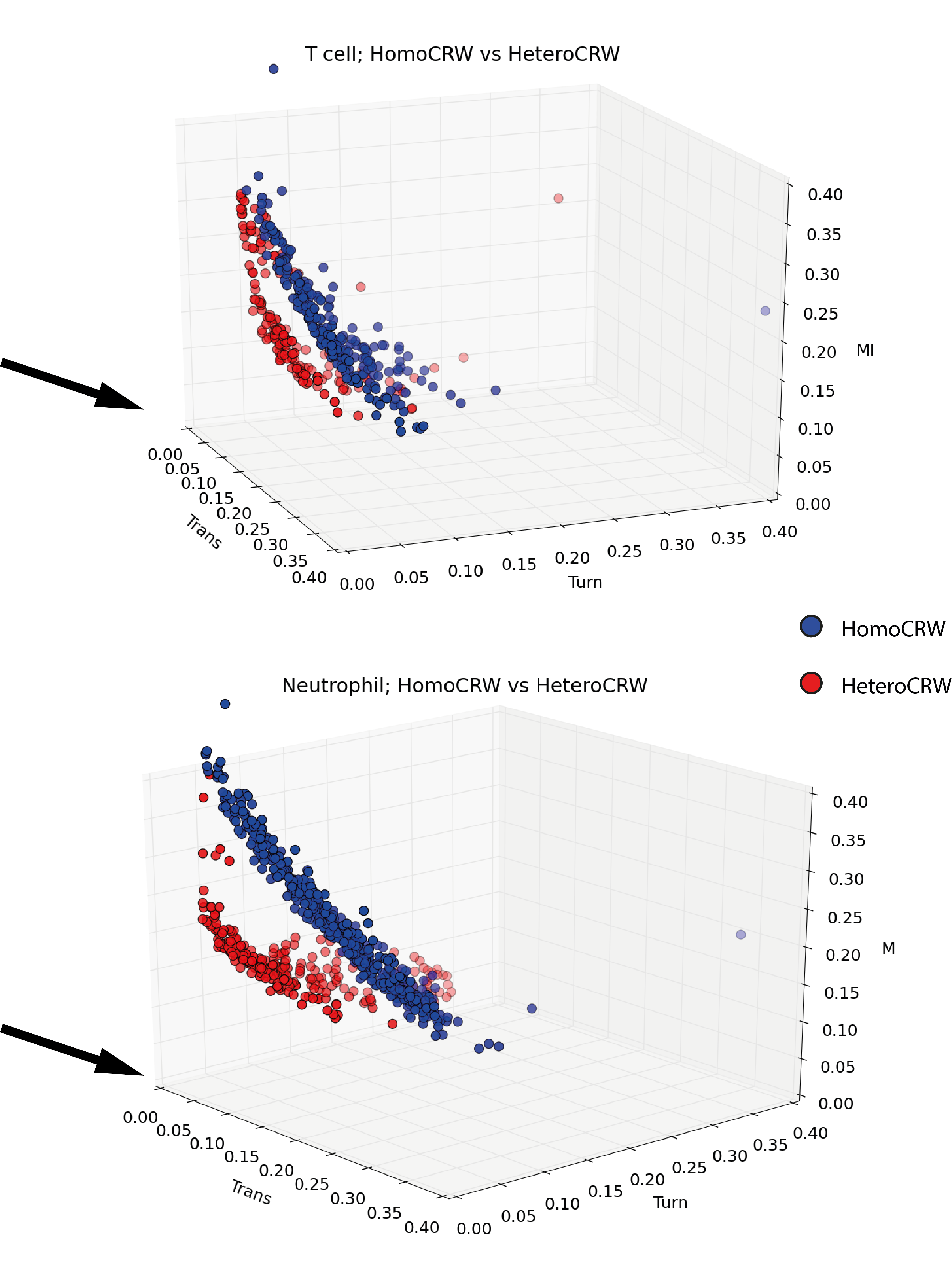

Supplement: S25 Fig — Calibration was against T cell data (top), or neutrophil data (bottom). ‘Trans’, translation speed KS values; ‘Turn’, turn speed KS values; ‘MI’, meandering index KS values. The large arrow identifies the origin, which represent a perfect reproduction of in vivo motility dynamics. Faded color dots lie further from the viewer. Plots have been rotated to emphasize the separation between the two Pareto fronts. (PNG) [file pcbi.1005082.s026.png]

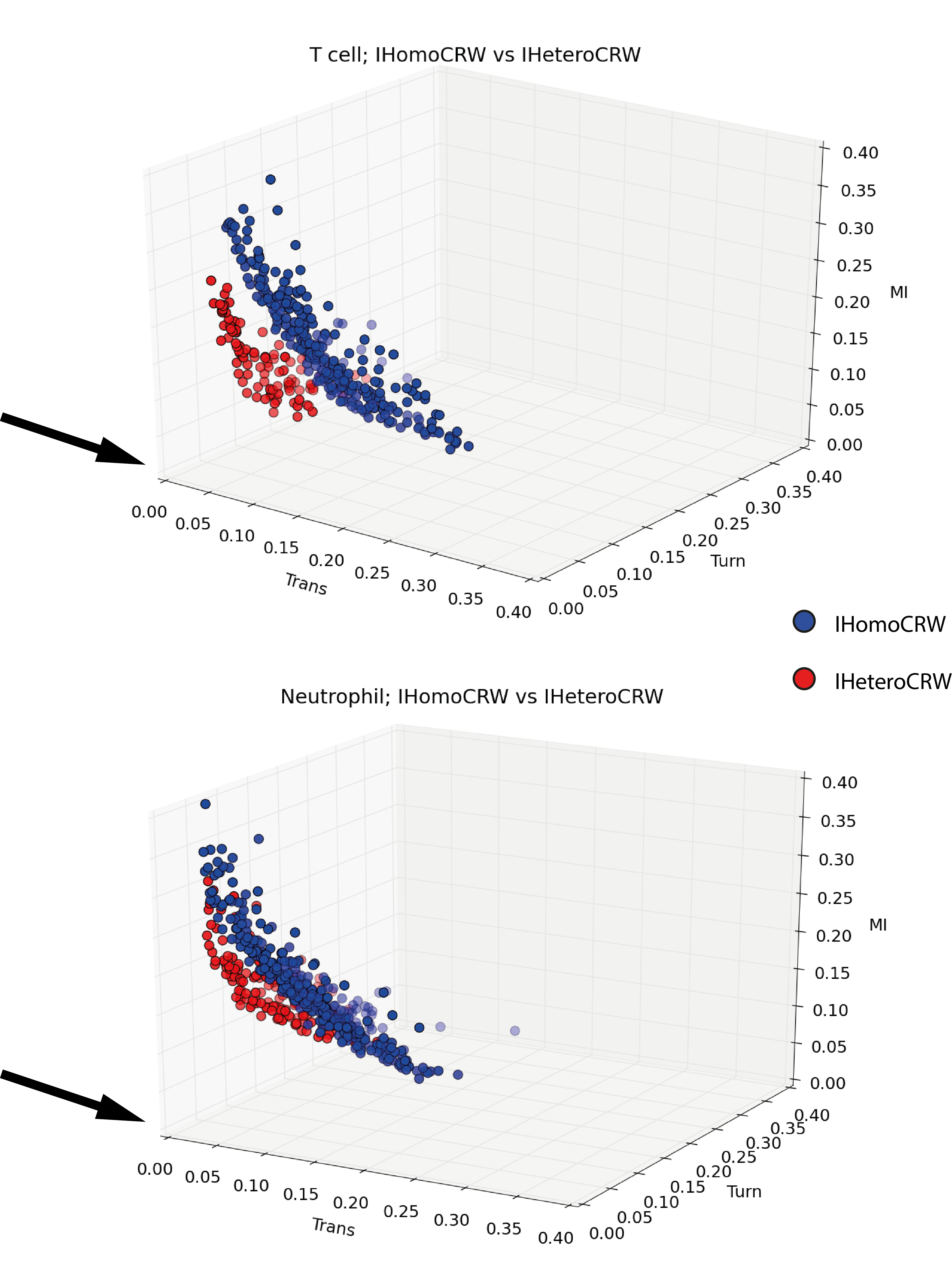

Supplement: S26 Fig — Calibration was against T cell data (top), or neutrophil data (bottom). ‘Trans’, translation speed KS values; ‘Turn’, turn speed KS values; ‘MI’, meandering index KS values. The large arrow identifies the origin, which represent a perfect reproduction of in vivo motility dynamics. Plots have been rotated to emphasize the separation between the two Pareto fronts. (PNG) [file pcbi.1005082.s027.png]

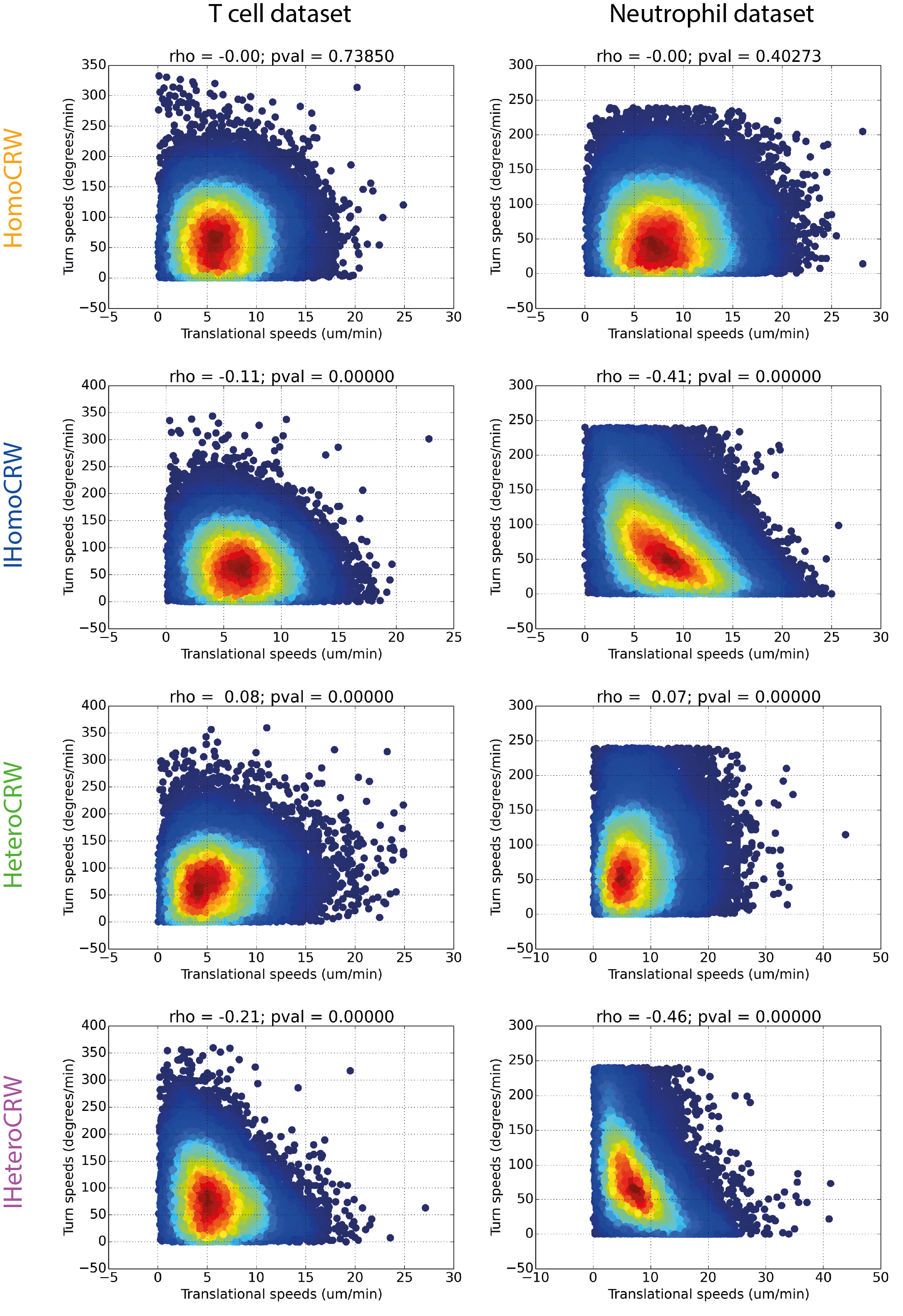

Supplement: S27 Fig — IHomoCRW and IHeteroCRW explicitly prescribe a negative correlation between translation and turn speeds, motivated by finding such a correlation in our in vivo data. Data shown represent the best solutions, as determined by lowest Λ value, for each model when calibrated against each dataset. (PNG) [file pcbi.1005082.s028.png]

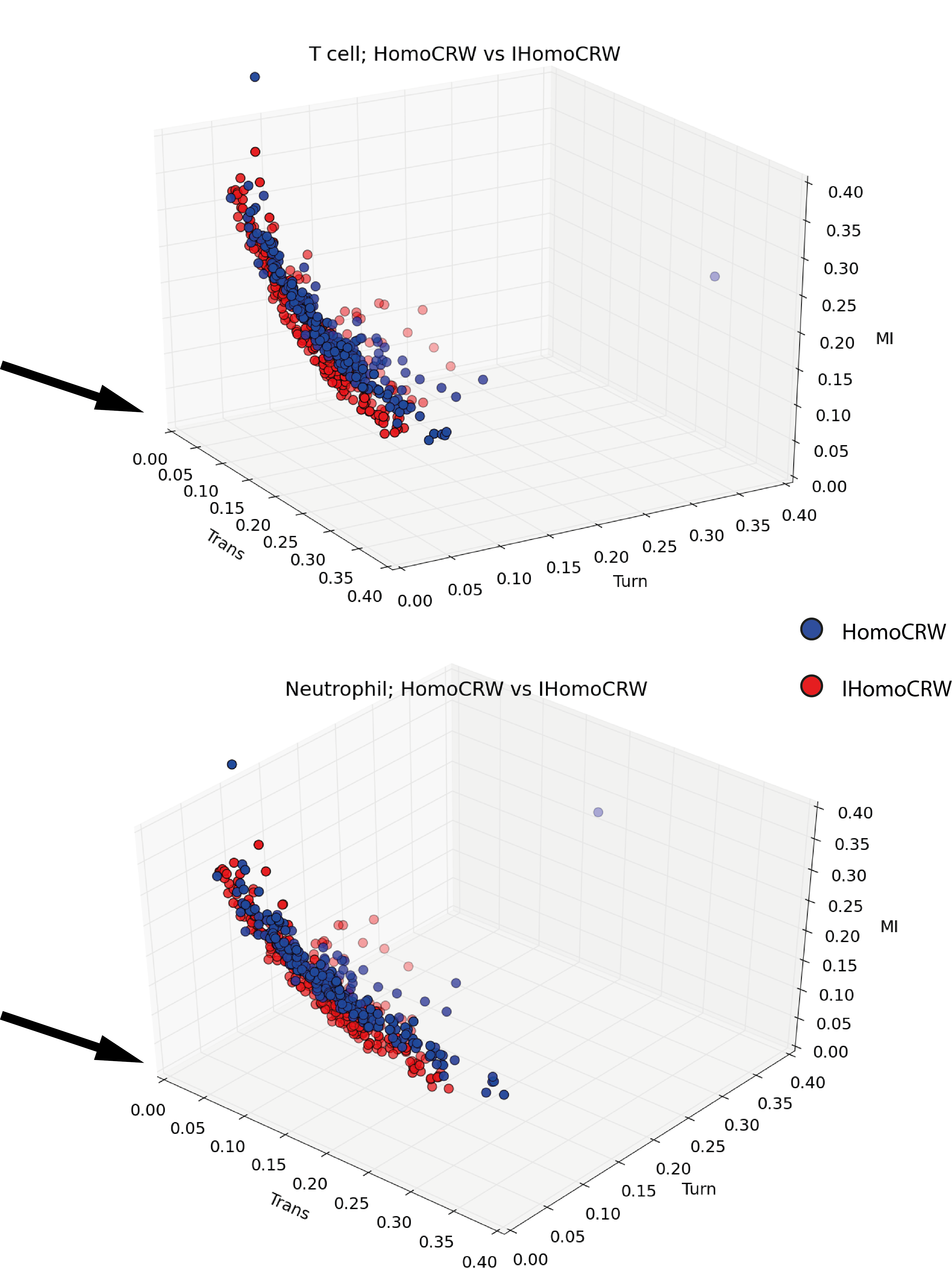

Supplement: S28 Fig — Calibration was against T cell data (top), or neutrophil data (bottom). ‘Trans’, translation speed KS values; ‘Turn’, turn speed KS values; ‘MI’, meandering index KS values. The large arrow identifies the origin, which represent a perfect reproduction of in vivo motility dynamics. Faded color dots lie further from the viewer. Plots have been rotated to emphasize the separation between the two Pareto fronts. (PNG) [file pcbi.1005082.s029.png]

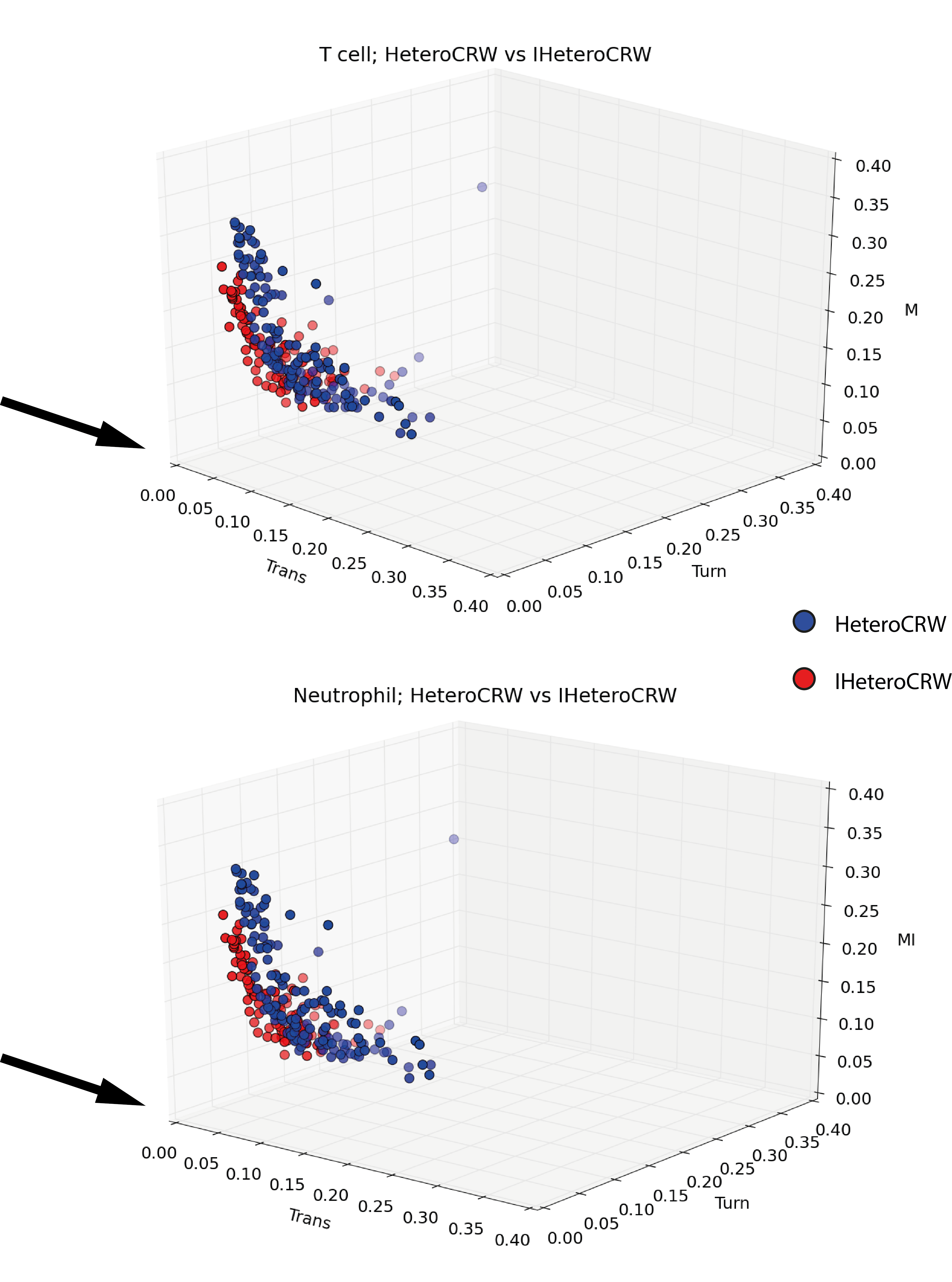

Supplement: S29 Fig — Calibration was against T cell data (top), or neutrophil data (bottom). ‘Trans’, translation speed KS values; ‘Turn’, turn speed KS values; ‘MI’, meandering index KS values. The large arrow identifies the origin, which represent a perfect reproduction of in vivo motility dynamics. Plots have been rotated to emphasize the separation between the two Pareto fronts. (PNG) [file pcbi.1005082.s030.png]

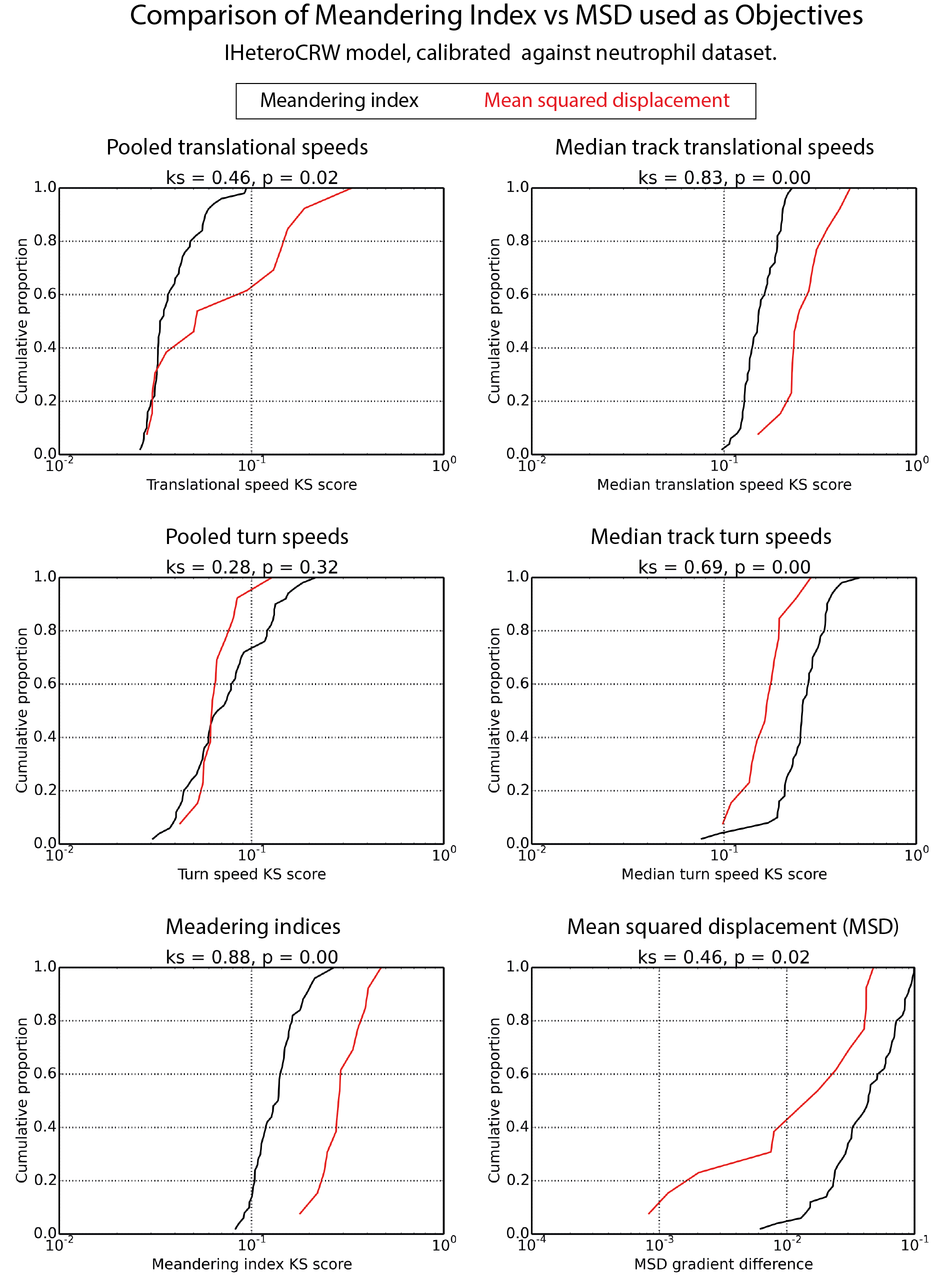

Supplement: S30 Fig — The IHeteroCRW model was calibrated against neutrophil data, using either the meandering index or the MSD as a calibration objective. The MSD objective comprises the absolute difference between linear-regression fitted gradients applied to neutrophil and IHeteroCRW candidate solution data. The pooled translational and turn speeds were used as the other two objectives. Calibration was performed three independent times in both cases, shown are the performances of Pareto front solutions of all three calibrations pooled together. (PNG) [file pcbi.1005082.s031.png]

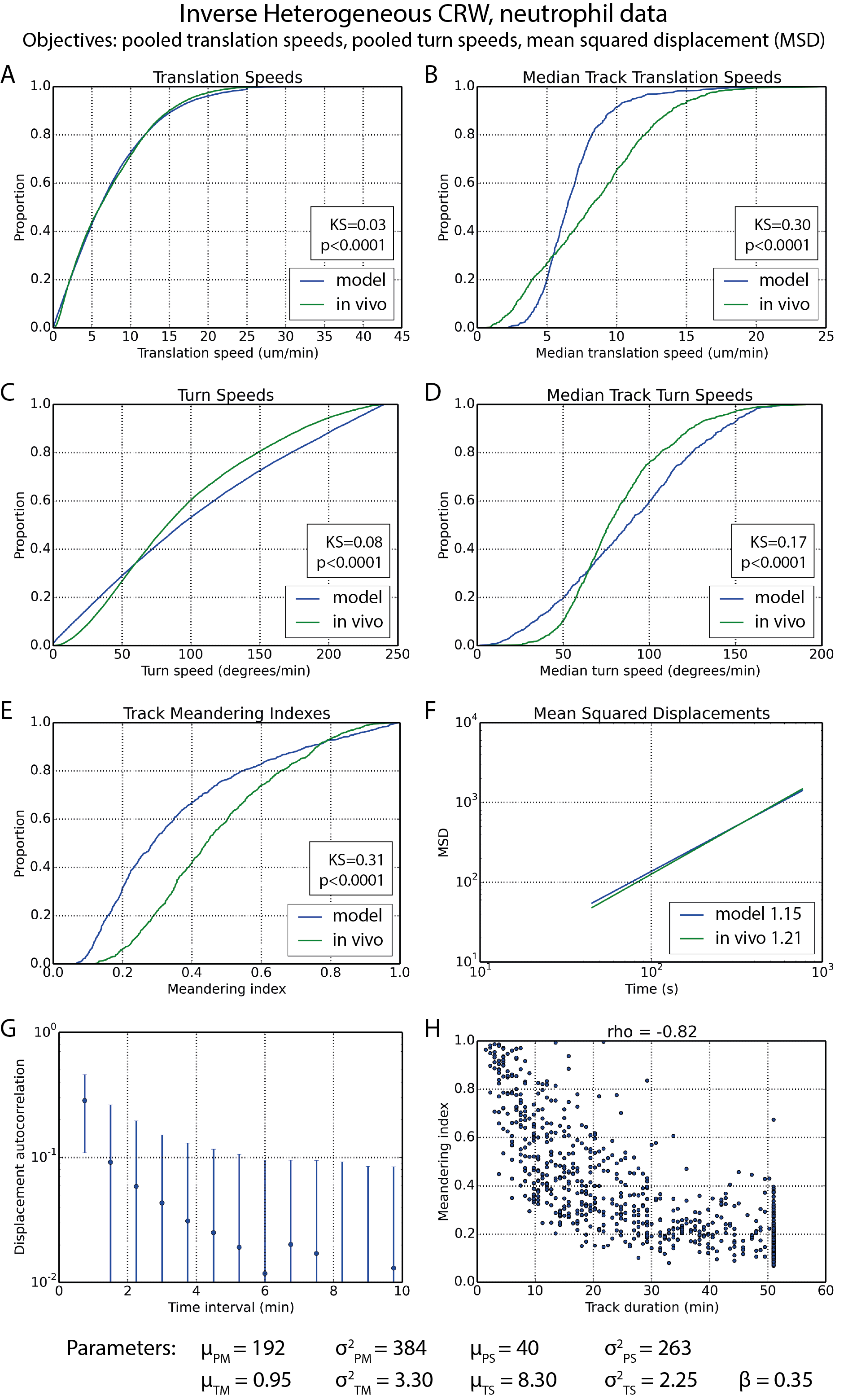

Supplement: S31 Fig — The best solution is that with the lowest Λ value. Pooled (A) and median track (B) translational speed distributions are shown as cumulative distribution plots. Similar plots, (C) and (D), depict turn speed data. (E) Cumulative distribution plot of track meandering index distributions. (F) Mean squared displacements for given durations (anywhere in the temporal domain, not from time zero only) plotted on log-log axes. The gradients of linear regression fitted models are given. (G) Displacement autocorrelations for the given time intervals; dots represent median values with error bars covering data lying within the interquartile range at each time interval. (H) Scatter plot of track meandering indices against duration, Spearman’s rank correlation coefficient is given. The model’s parameter values are given. Note that model calibration was performed using metrics of panels A, B and F only. See Methods for more details. (PNG) [file pcbi.1005082.s032.png]

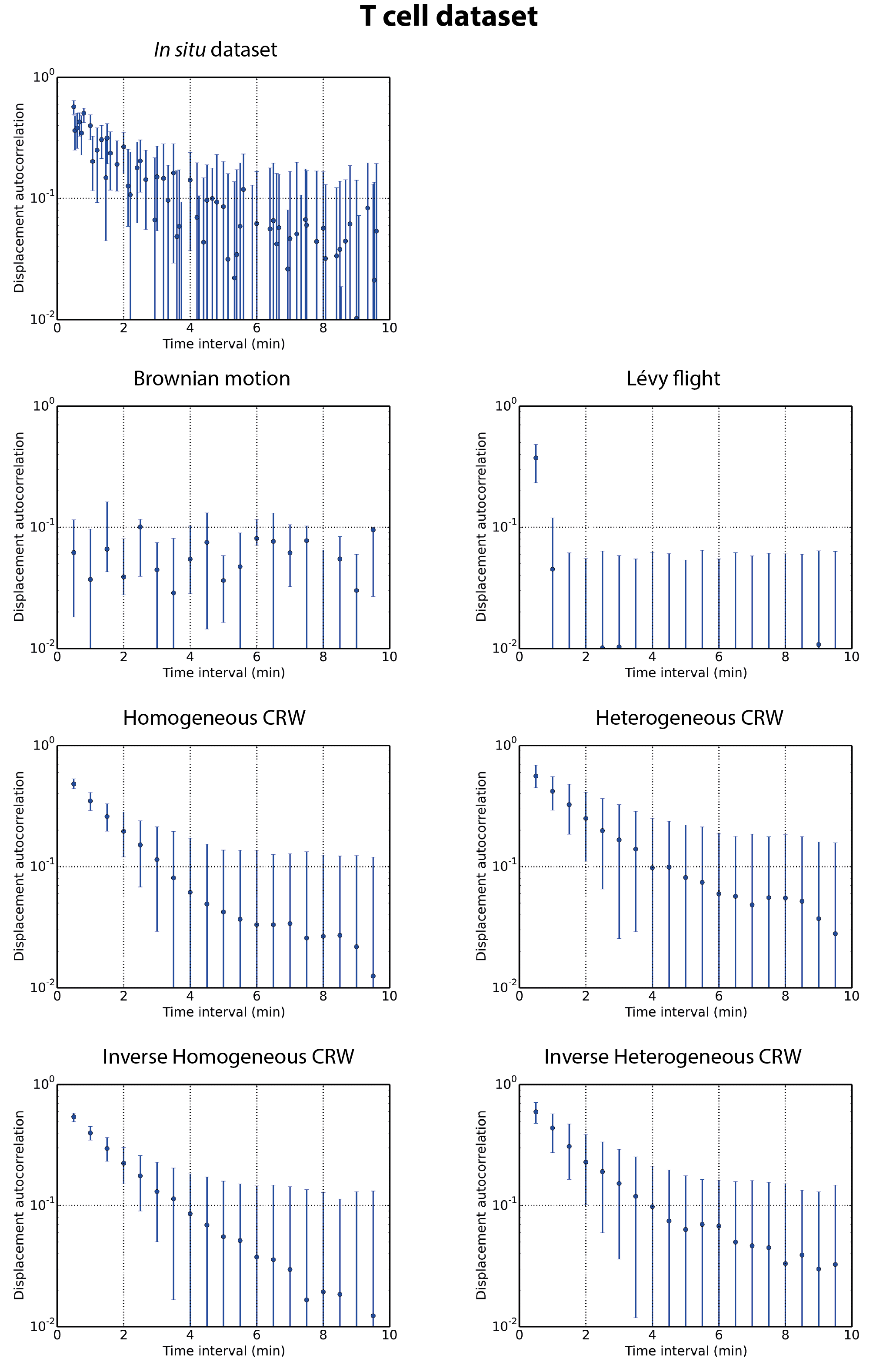

Supplement: S32 Fig — Dots represent median values, with error bars covering data lying within the interquartile range at each time interval. Irregular intervals for in vivo data reflect imaging experiments with differing time-steps. For methodological details see [21]. The CRW models best reflect the in vivo dynamics. (PNG) [file pcbi.1005082.s033.png]

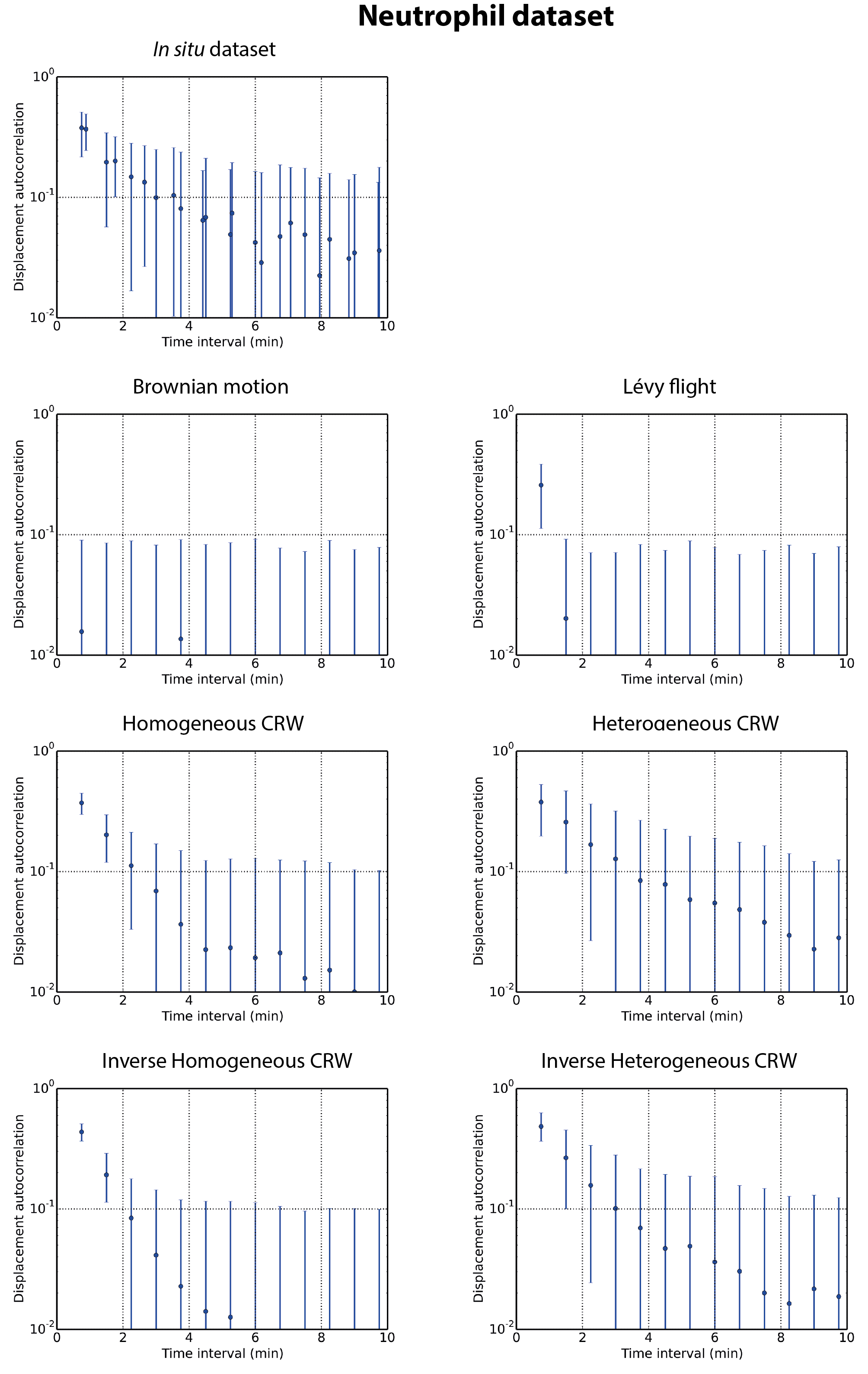

Supplement: S33 Fig — Dots represent median values, with error bars covering data lying within the interquartile range at each time interval. Irregular intervals for in vivo data reflect imaging experiments with differing time-steps. For methodological details see [21]. The CRW models best reflect the in vivo dynamics. (PNG) [file pcbi.1005082.s034.png]

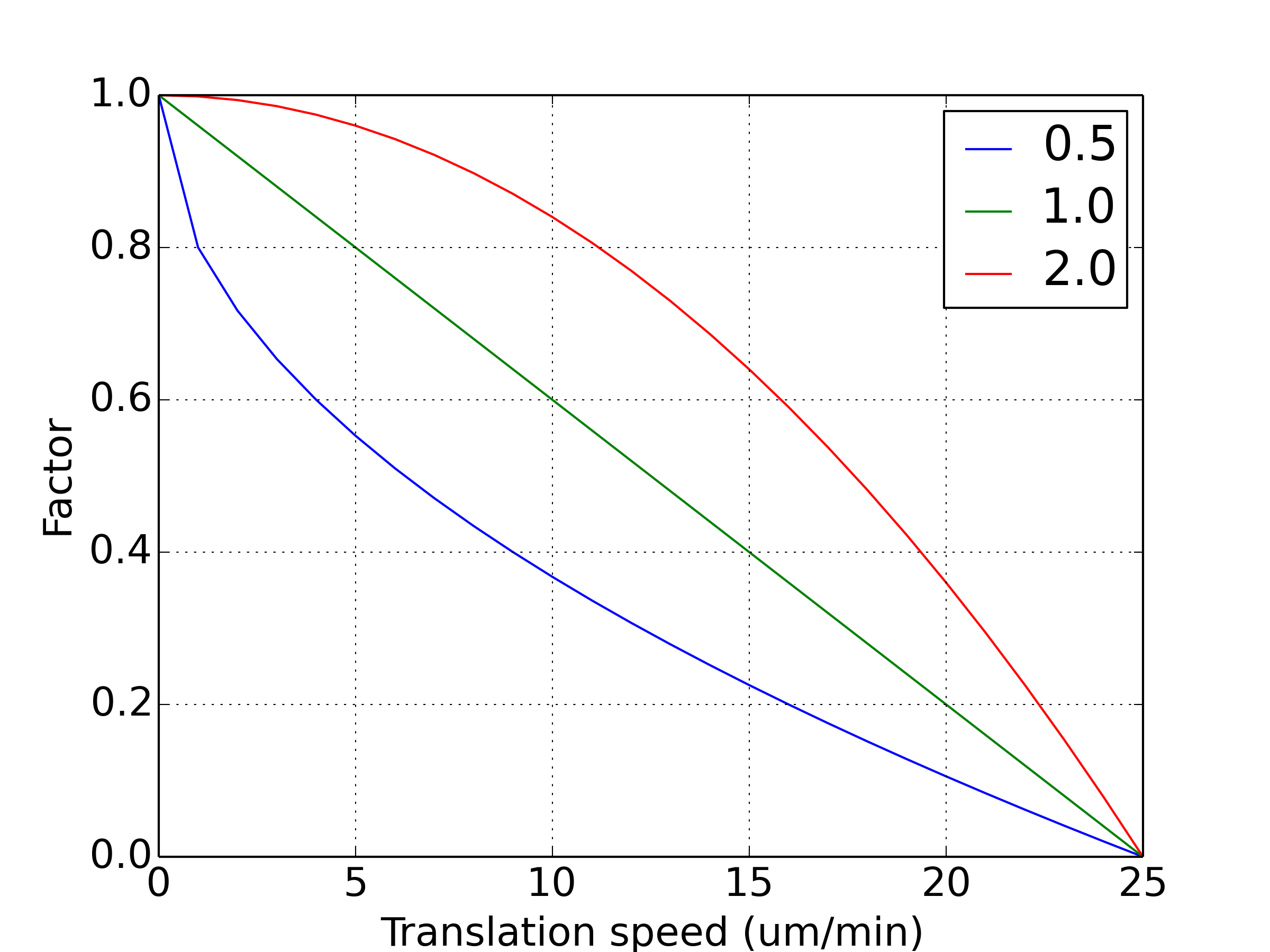

Supplement: S34 Fig — It is a parameter of the IHomoCRW and IHeteroCRW models. (PNG) [file pcbi.1005082.s035.png]

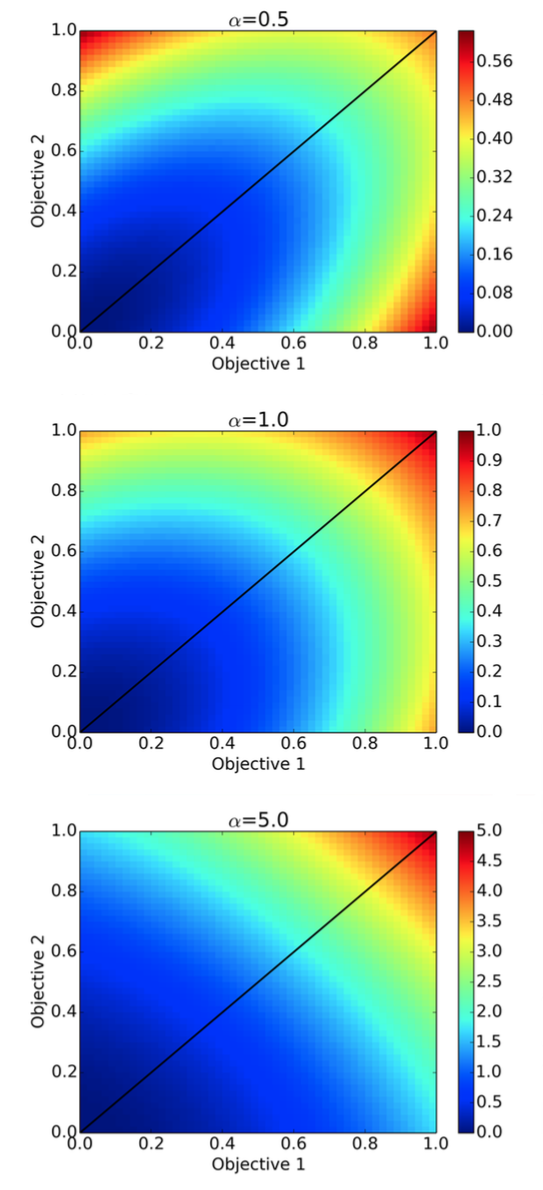

Supplement: S35 Fig — α values of (top) 0.5, (middle) 1.0 and (bottom) 5.0 are shown. The black line indicates y = x. Note that the range of Λ values changes with α, and hence comparisons between solution and Pareto front Λ values are valid only when generated using the same α value. (PNG) [file pcbi.1005082.s036.png]

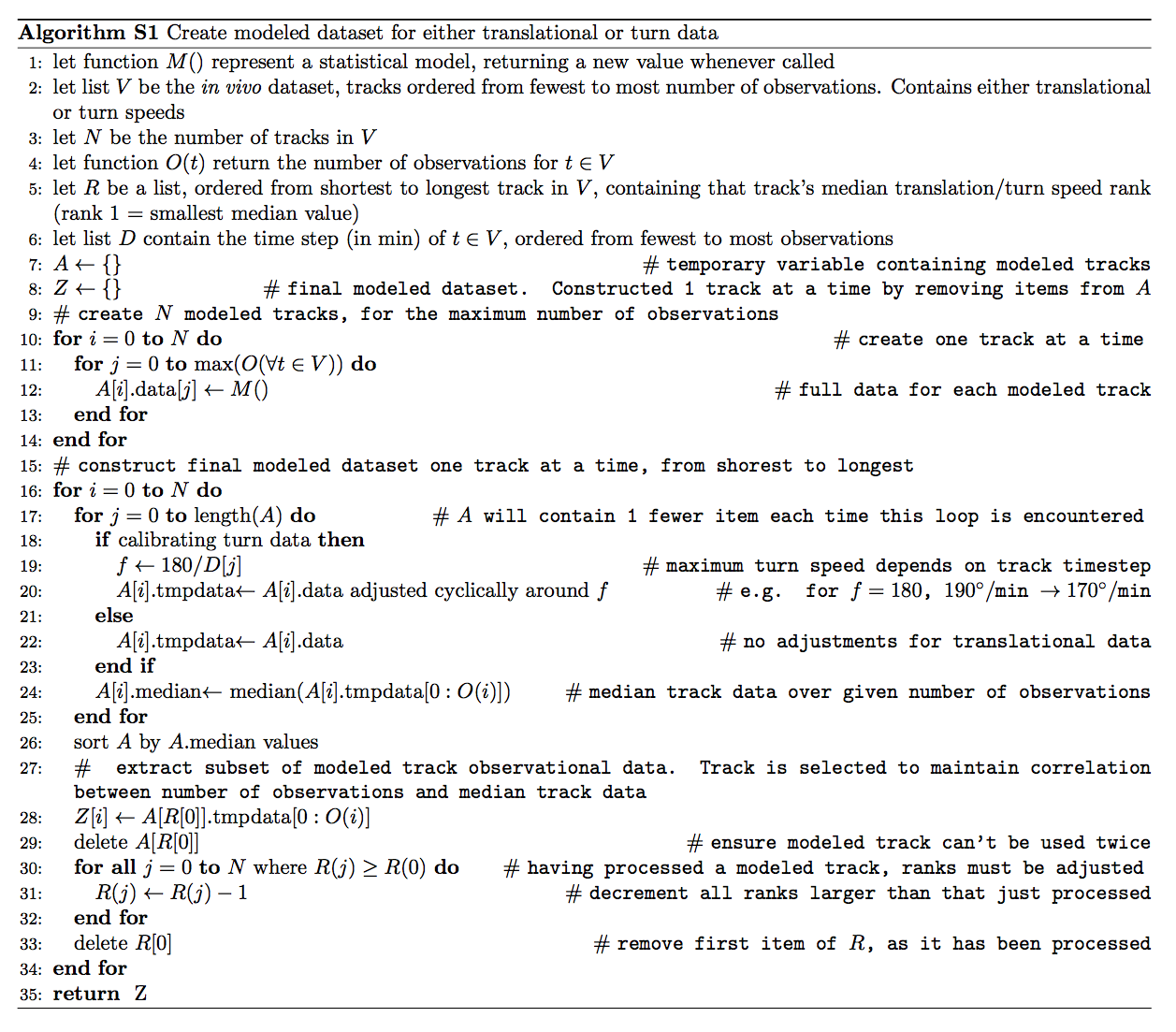

Supplement: S1 Algorithm — The dataset produced contains the same number of data items, spread across the same number of tracks each with the same number of observations as the in vivo dataset. The algorithm accounts for the time-step duration of each in vivo track (which differed between imaging experiments) when adjusting the maximum recognizable turn speed (does not apply for translational data). The negative correlation between track duration and median track translational speed, and the positive correlation between track duration and median track turn speed, as found in the in vivo datasets are maintained. (PNG) [file pcbi.1005082.s037.png]
